# ATRX/EZH2 complex epigenetically regulates FADD/PARP1 axis, contributing to TMZ resistance in glioma

**DOI:** 10.7150/thno.41219

**Published:** 2020-02-10

**Authors:** Bo Han, Xiangqi Meng, Pengfei Wu, Ziwei Li, Siyi Li, Yangong Zhang, Caijun Zha, Qile Ye, Chuanlu Jiang, Jinquan Cai, Tao Jiang

**Affiliations:** 1 Beijing Neurosurgical Institute, Capital Medical University, Beijing 100050, China; 2 Department of Neurosurgery, the Second Affiliated Hospital of Harbin Medical University, Neuroscience Institute, Heilongjiang Academy of Medical Sciences, Harbin 150086, China; 3 Department of Neurosurgery, Beijing Tiantan Hospital, Capital Medical University, Beijing, 100050, China; 4 Department of Mining and Materials Engineering, McGill University, Montreal, QC H2X1X8, Canada; 5 Department of Laboratory Diagnosis, the Second Affiliated Hospital of Harbin Medical University, Harbin 150086, China.

**Keywords:** glioma, ATRX, TMZ resistance, PARP1, EZH2

## Abstract

**Rationale**: Glioma is the most common primary malignant brain tumor in adults. Chemoresistance of temozolomide (TMZ), the first-line chemotherapeutic agent, is a major issue in the management of patients with glioma. Alterations of alpha thalassemia/mental retardation syndrome X-linked (ATRX) gene constitute one of the most prevalent genetic abnormalities in gliomas. Therefore, elucidation of the role of ATRX contributing to TMZ resistance in glioma is urgently needed.

**Methods**: We performed the bioinformatics analysis of gene expression, and DNA methylation profiling, as well as RNA and ChIP-seq data sets. CRISPR-Cas9 gene editing system was used to achieve the ATRX knockout in TMZ resistant cells. In vitro and in vivo experiments were carried out to investigate the role of ATRX contributing to TMZ resistance in glioma.

**Results**: We found that ATRX expression was upregulated via DNA demethylation mediated by STAT5b/TET2 complex and strengthened DNA damage repair by stabilizing PARP1 protein in TMZ resistant cells. ATRX elicited PARP1 stabilization by the down-regulating of FADD expression via the H3K27me3 enrichment, which was dependent on ATRX/EZH2 complex in TMZ resistant cells. Magnetic resonance imaging (MRI) revealed that the PARP inhibitor together with TMZ inhibited glioma growth in ATRX wild type TMZ resistant intracranial xenograft models.

**Conclusions**: The present study further illustrated the novel mechanism of the ATRX/PARP1 axis contributing to TMZ resistance. Our results provided substantial new evidence that PARP inhibitor might be a potential adjuvant agent in overcoming ATRX mediated TMZ resistance in glioma.

## Introduction

Glioma is the most common primary malignant brain tumor in adults 1. Despite the development of aggressive comprehensive treatments, the median overall survival (OS) of patients with glioblastoma (GBM), the most malignant type of glioma remains to be approximately 12 to 15 months 2, 3. Similar to other malignant tumors, GBM exhibits a distinct anti-DNA damage phenotype, which is responsible for chemoresistance 4. Although TMZ is the first-line treatment of patients with GBM, resistance to the drug is inevitable in an overwhelming majority of patients 5, 6. Thus, it is worthwhile exploring the underlying mechanism regulating DNA damage repair to overcome TMZ resistance in GBM treatment.

Alpha thalassemia/mental retardation syndrome X-linked (ATRX) is one of the SWI/SNF-like families for chromatin remodeling. Loss of ATRX constitutes one of the most prevalent genetic abnormalities in gliomas 7. ATRX plays a role in remodeling and stabilizing genome by medicating deposition of H3.3 at chromatin 8. ATRX could also recruit modification enzymes and facilitate the maintenance of H3K9me3 to regulate activation of the ataxia telangiectasia mutated (ATM) signaling pathway 9. Besides, loss of ATRX was associated with epigenetic alterations, including abnormal levels of DNA methylation in murine cells 10. In our previous study, CRISPR-Cas9 mediated genetic inactivation of *ATRX* was observed to inhibit cell growth and cause more DNA damage induced by TMZ in glioma 11. Therefore, it is imperative to elucidate further the role of ATRX contributing to TMZ resistance in glioma.

PARP1, the crucial member of poly (ADP-ribose) polymerase family, plays an important role in regulating biological behavior including DNA repair pathways 12. PARP1 catalyzes the binding of ADP-ribose in the process of regulating DNA repair. PARP cleavage, as one hallmark of apoptosis and caspase activation, is often associated with abrogated DNA damage response 13. Hence, inhibition of PARP1 induces DNA double strand breaks (DSBs) in tumors 14 and PARP inhibitors (PARPi) represent promising agents for cancers 15. ATRX deficiency was reported to impair DNA damage repair reduing non-homologous end joining (NHEJ) 16, which was regulated by PARP through the retention of NHEJ proteins at DSBs 17. However, the interactions between ATRX and PARP1 during DNA damage repair needs further investigation.

Fas-associated death domain (FADD) serves as a necroptosis factor regulating PARP1 cleavage 18, 19. FADD interacts with the death domain of receptors, leading to the activation of cysteine-aspartic acid specific protease-8 (CASP8), which subsequently activates several downstream caspases and finally induces apoptosis 20. Apoptosis appears to be inhibited in cells lacking functional FADD following treatment with cytotoxic agents 21, 22. Thus, we proposed that FADD/PARP1 axis might be implicated in ATRX mediated DNA damage repair during TMZ resistance.

In the present study, we investigated the role of ATRX in regulating TMZ resistance in glioma. Our results indicated that ATRX was upregulated via DNA demethylation mediated by STAT5b/TET2 complex in TMZ resistant glioma cells and that ATRX promoted PARP1 stabilization through down-regulating FADD by suppressing of H3K27me3 enrichment in *FADD* promoter region. PARPi together with TMZ inhibited glioma growth in TMZ resistant xenograft models, indicating that the PARP inhibitor might be a potential adjuvant agent in overcoming ATRX mediated TMZ resistance in gliomas.

## Methods

***Data collection and tissue specimens*:** In the present study, all datasets were obtained from the following public websites: The Cancer Genome Atlas (TCGA, https://portal.gdc.cancer.gov), Chinese Glioma Genome Atlas (CGGA, http://www.cgga.org.cn/), GSE16011 (https://www.ncbi.nlm.nih.gov) and the Repository for Molecular Brain Neoplasia Data (REMBRANDT, http://cabig.cancer.gov/solutions/conductresearch/rembrandt). The protein-protein interaction analysis was performed using the STRING website (https://string-db.org). The ChIP-seq data were obtained from the Gene Expression Omnibus (GEO, https://www.ncbi.nlm.nih.gov/geo/, GSM1577745, GSM1577750, GSM2274677, GSM3498421, GSM2573751, GSM2573755). Molecular Signature Database (MSigDB, http://software.broadinstitute.org) was used to perform gene family annotation. The patient samples used in the present study were confirmed by two independent pathologists. Informed consent was obtained from patients involved in this study and the study protocol was approved by the Clinical Research Ethics Committee of the Second Affiliated Hospital of Harbin Medical University.

***Cell culture and lentivirus infection*:** The human GBM cell line LN229 was obtained from the Institute of Biochemistry and Cell Biology, Chinese Academy of Science. Patient derived GBM cell lines HG7 and HG9 were obtained from two patients with primary GBM. The extraction procedure was described previously 23. Briefly, tumor tissue was washed, mechanically minced in MACS^TM^ C Tubes (Miltenyi Biotec, Germany) and digested with 0.1% trypsin (Invitrogen, USA) ,10 U /mL of DNase I (Promega, USA) at 37 °C for 45 minutes and neutralized by minimum essential medium (MEM-α) containing 10% fetal bovine serum (FBS). Red blood cells were lysed with ACK lysis buffer (Beyotime, Shanghai, China). The washed tissues were passed through a 100 μm cell strainer and cultured in Dulbecco's Modified Eagle Medium (DMEM/F12) supplemented with N2, B27 (Gibco), 20 ng/mL human fibroblast growth factor-basic (bFGF, Sino Biological). LN229 cells were cultured in DMEM/F12 1:1 medium (Corning) containing 10% FBS. Cells were maintained at 37 ℃ under a humidified atmosphere containing 5% CO_2_ and authenticated using STR assay (Genetic Testing Biotechnology, Jiangsu, China). LN229R cells were infected with lentiviruses carrying shRNA (sh-STAT5b or sh-TET2) or negative control (NC) to generate LN229R-shSTAT5b, LN229R-shTET2, or LN229R-NC cells, respectively. The details of the shRNA sequences were identical to the siRNA sequences shown in **Table S1**. TMZ (Selleck, Houston, TX, USA), the PARP1-inhibitor Olaparib (Selleck, Houston, TX, USA), or dimethylsulfoxide (Fisher Scientific, Waltham, MA, USA) were added to cell cultures as indicated.

***Establishment of TMZ-resistant cells*:** TMZ-resistant cells were established as described into our previous studies 23, 24. Briefly, LN229, HG7 and HG9 cells were seeded into 96-well plates, and IC50 (half maximal inhibitory concentration) of TMZ was determined by CCK-8 assay. TMZ was added from low concentration (LN229: 1 μM, HG7: 1.2 μM, HG9: 1.5 μM) to high concentration (LN229: 500 μM, HG7: 614.4 μM, HG9: 768 μM) for five months. When the cells grew stably, the drug dose was increased in multiples. Each dose was maintained for 15 days to the end of the fifth month. The induced TMZ-resistant cells were named as LN229R, HG7R and HG9R.

***Pyrosequencing*:** Genomic DNA was extracted from LN229, LN229R, LN229R-NC, LN229R-shTET2, and LN229R-shSTAT5b cells by using QIAamp DNA Mini Kit (Qiagen, China) and treated with bisulfite by using the EpiTect Bisulfite Kit (Qiagen, China). Pyrosequencing analysis was carried out by Oebiotech Company (Shanghai, China). Methylation values >10% in GBM cells were considered to be methylated.

***Immunoblotting*:** Western blot (WB), immunofluorescence (IF), and immunohistochemistry (IHC) assays were performed as previously described 25 and the antibodies are listed in **Table S2**. Quantitative evaluation was performed by examining each section using at least 10 different high-power fields with the most abundant stained cells as described previously 24, 26. The representative imaged fields were determined by the average method. Co-immunoprecipitation (co-IP) assays were performed according to the manufacturer's protocol for Protein A/G Magnetic Beads for IP (Bimake).

***CRISPR-Cas9 mediated gene editing and siRNA transfection*:** CRISPR-Cas9 based ATRX knockout was performed as described 11. Cells were infected with viruses containing the CAS9 gene and were selected with 10 µg/mL puromycin for 7 days. The cells were subsequently infected with virus carrying small guide RNAs (sgRNAs) designed for ATRX. The infection proceeded for 24 hours and the ATRX knockout was confirmed by WB and qRT-PCR. For siRNA transfection, cells were plated in 6-well plates (2000 μL medium/well) and cultured overnight followed by transfection with siRNA or control siRNA for another 48 hours. Knockdown efficiency was confirmed by WB. The details of the sgRNA or siRNA sequences are shown in **Table S1**.

***Microarray analysis*:** The RNA expression profiling was performed using Agilent custom human mRNA microarrays (SHBIO Biotechnology Corporation, Shanghai, China). The raw data were normalized using the quantile algorithm from the limma package in R. Heatmaps representing differentially regulated genes were generated using Cluster 3.0 and Gene Tree View. The microarray data were deposited in NCBI's GEO (Gene Expression Omnibus) database (www.ncbi.nlm.nih.gov/geo) under accession number GSE131781.

***Luciferase assay*:** Genomic DNA fragments of the human *ATRX* gene, spanning from +1 to -3000 relative to the transcription initiation site were generated by PCR and inserted into pGL3-Basic vectors (denoted as pGL3-*ATRX*). HG7R and HG9R cells were transfected with pGL3-*ATRX* for 24 hours. Transfection was performed with pRL construct containing Renilla reniformis luciferase gene, which was used as normalizing control. Luciferase assays were performed using Dual Luciferase Assay System (Promega). Relative luciferase activity was defined as the ratio of firefly luciferase activity to R. reniformis luciferase activity. Error bars represent standard deviation of the three experiments.

***F-actin staining assay and 5-ethynyl-20-deoxyuridine (EdU) labeling*:** F-actin staining was performed using TRITC-labeled Phalloidin (Yeasen). In brief, cells were seeded on tissue culture-treated glass coverslips. After treatment, cells were washed, fixed, permeabilized, blocked and incubated with 100 nM phalloidin at 37 ℃ followed by immunofluorescence staining. EdU labeling was performed according to the manufacturer's protocol (Ribobio, Guangzhou, China).

***CASP 3/7 activity assay, ATP measurement, and Annexin-V/PI cell apoptosis detection*:** For caspase 3/7 activity assay, 2.5×10^4^ cells were seeded in 96-well plates and treated as indicated. Caspase 3/7 activity was then measured using the Caspase-Glo 3/7 Assay Kit (Promega). For relative ATP measurement assays, cells were seeded into 24-well plates and treated as indicated. For analysis, plates were incubated for 30 minutes at room temperature, 200 µL of CellTiter-Glo reagent (Promega) was added to each well and plates were agitated on an orbital shaker for 12 minutes. Luminescence was quantified on a standard plate luminometer. The apoptosis detection was also performed using the APC Annexin V Apoptosis Detection Kit I (BD Biosciences). A total of 1×10^6^ cells were collected for each group and were stained with FITC and PI following the manufacturer's protocol. All experiments were performed in triplicate.

***Chromatin immunoprecipitation (ChIP) and quantitative real-time reverse transcription-polymerase chain reaction (qRT-PCR)*:** ChIP experiments were performed using the Chromatin immunoprecipitation (ChIP) Assay Kit (Millipore, 17-295) and the antibodies (**Table S2**) following the manufacturer's protocol. Briefly, 4 ×10^7^ cells were used to determine total DNA input and incubated overnight with specific antibodies or with normal rabbit immunoglobulin A/G. After the precipitated DNA was purified and rehydrated using the kit, the sample was analyzed by quantitative real-time PCR performed in triplicate using the CFX-96 PCR system (Bio-Rad, Berkeley, CA, USA). Precipitated DNA levels of ATRX and FADD promoter were determined with SYBR PrimeScript RT-PCR Kit (TaKaRa, Otsu, Japan) according to the manufacturer's protocol.

Total RNA was extracted using RNAiso Plus reagent (TaKaRa, Otsu, Japan). The cDNAs were synthesized with a PrimeScript RT reagents kit (TaKaRa) according to manufacturer's instructions. Endogenous mRNA levels were determined using the SYBR PrimeScript RT-PCR Kit (TaKaRa). The qRT-PCR data were analyzed using the 2^-ΔΔ^Ct method. PCR primers designed and synthesized by Invitrogen (Shanghai) were listed in **Table S3**.

***Vascular mimicry assay, cell viability analysis, soft agar colony formation, and colony formation assay*:** Vascular mimicry assay was performed as described 11. Treated cells were seeded into 48-well dishes pre-coated with Matrigel. Tube formation was assessed using a phase-contrast microscope 12 hours after seeding. CCK-8 (Dojindo, Kumamoto, Japan) was used to determine the number of viable cells according to the manufacturer's protocol. Cell viability was measured by optical absorbance on an Epoch plate reader (Biotek Instruments, Winooski, VT, USA). For colony formation assays, 3.5 cm culture dishes were seeded with 1500 cells and treated as indicated. The assays were stopped when the colonies could be observed clearly with the naked eye. Then, cells were fixed with methanol and stained with 0.1% crystal violet for recording colony numbers. For the soft agar assay, 24-well plates were pre-coated with 0.5 mL 1× concentration complete 1640 medium containing 0.6% agarose (Yeason, Shanghai, China) and 10% FBS. Tumor cells were suspended in 1 mL complete 1640 medium containing 0.35% agarose treated as indicated and incubated at 37 ℃ with 5% CO_2_ for 3 weeks. Cell colonies were stained with 0.1% crystal violet for 15 minutes and observed using a Bio-Rad ChemiDoc™ imaging system (Berkeley, CA, USA). All experiments were performed in triplicate.

***Transwell invasion and comet assay*:** The transwell invasion assay was performed with small modifications: in 24-well cell culture chambers with transwell inserts (Corning, 8-mm pore) precoated with Matrigel. In brief, treated cells were seeded at a density of 5 × 10^4^ cells per upper well in 200 µL culture containing 4% FBS and 500 μL medium containing 50% FBS was added to the lower chambers. After 24 hours, the upper surface was removed by scrubbing with a cotton-tipped stick, whereas the lower surface was fixed with methanol for 5 minutes, air-dried, and stained with hematoxylin and eosin (H&E). Cell numbers were captured in six different photographs.

For the comet assay, cells were seeded in 12-well plates overnight and treated with TMZ and/or olaparib at final concentrations of 200 μM and 1 μM respectively, for 12 hours and collected. Cells were resuspended in 0.75% agarose and placed on glass slides. Subsequently, the cells were lysed in lysis buffer following manufacturer's instructions (Trevigen, Gaithersburg, MD, USA) for 1 hour. The slides were then electrophoresed for 30 minutes at a voltage 21. The DNA was stained with 1×SYBRGreen (Sangon, Shanghai, China) and visualized under a confocal microscope. All experiments were performed in triplicate.

***Tumor xenograft study*:** Briefly, 3×10^5^ cells (LN229/LN229R or HG7R ATRX NC/KO) were injected into 5-week-old female BALB/c athymic nude mice. Seven mice per group were used in the experiment. The injection was performed as described in a previous publication 27. HG7R ATRX NC/KO mice were grouped into three groups that were treated with control, TMZ (60 mg/kg/day via intra-peritoneal injection) alone, and olaparib (50 mg/kg/day via intra-peritoneal injection) combined with TMZ for 7 days. Tumor volume was detected by BioSpec 94/20 USR (Bruker) nuclear magnetic resonance imaging (MRI) system.

***Statistical analysis*:** The Student *t*-test was used to determine the significance of differences between the two groups. A Chi-squared test was used to compare the binary and categorical patient characteristics between subgroups. Overall survival curves were plotted according to the Kaplan-Meier method and the log-rank test was applied for comparison. R packages, such as pheatmap, ggplot2 and corrplot were used to produce figures. Circos software (http://circos.ca) was used to visualize the differentially expressed genes between ATRX NC and ATRX KO cells. Bowtie2, Samtools, deeptools and MACS2 were used for ChIP-seq analysis. The Integrative Genomics Viewer (IGV, http://www.igv.org) was used to visualize ChIP-seq data. Gene ontology (GO) analysis was performed with the Gene Set Enrichment Analysis (GSEA, http://software.broadinstitute.org/gsea/index.jsp) software. AutoDock (http://autodock.scripps.edu) and PyMol (http://pymol.org) were used to calculate and visualize the combination of ATRX and EZH2 proteins. SPSS Graduate Pack 19.0 statistical software (SPSS, Chicago, IL, USA) and GraphPad Prime 7.0 (LaJolla, CA, USA) were used to calculate P value. P < 0.05 was considered statistically significant.

***Ethics approval and consent to participate*:** Human glioma tissues were obtained from glioma patients in the Second Affiliated Hospital of Harbin Medical University. Informed consent was obtained from patients enrolled in this study and the study protocol was approved by the Clinical Research Ethics Committee of the Second Affiliated Hospital of Harbin Medical University. BALB/c nude mice were purchased from the animal center of Beijing Vital River Laboratory Animal Technology and bred under special pathogen-free (SPF) conditions. This work followed protocols approved by the Institutional Committee on Animal Care of the Second Affiliated Hospital of Harbin Medical University.

***Data availability*:** Gene expression data reported in this study have been deposited with the Gene Expression Omnibus under the accession number GSE131781. All relative data supporting the finding of this study were available in the article or from the corresponding author upon a request.

## Results

### STAT5b/TET2 complex increases ATRX expression via DNA demethylation in TMZ resistant gliomas

**Figure 1:**
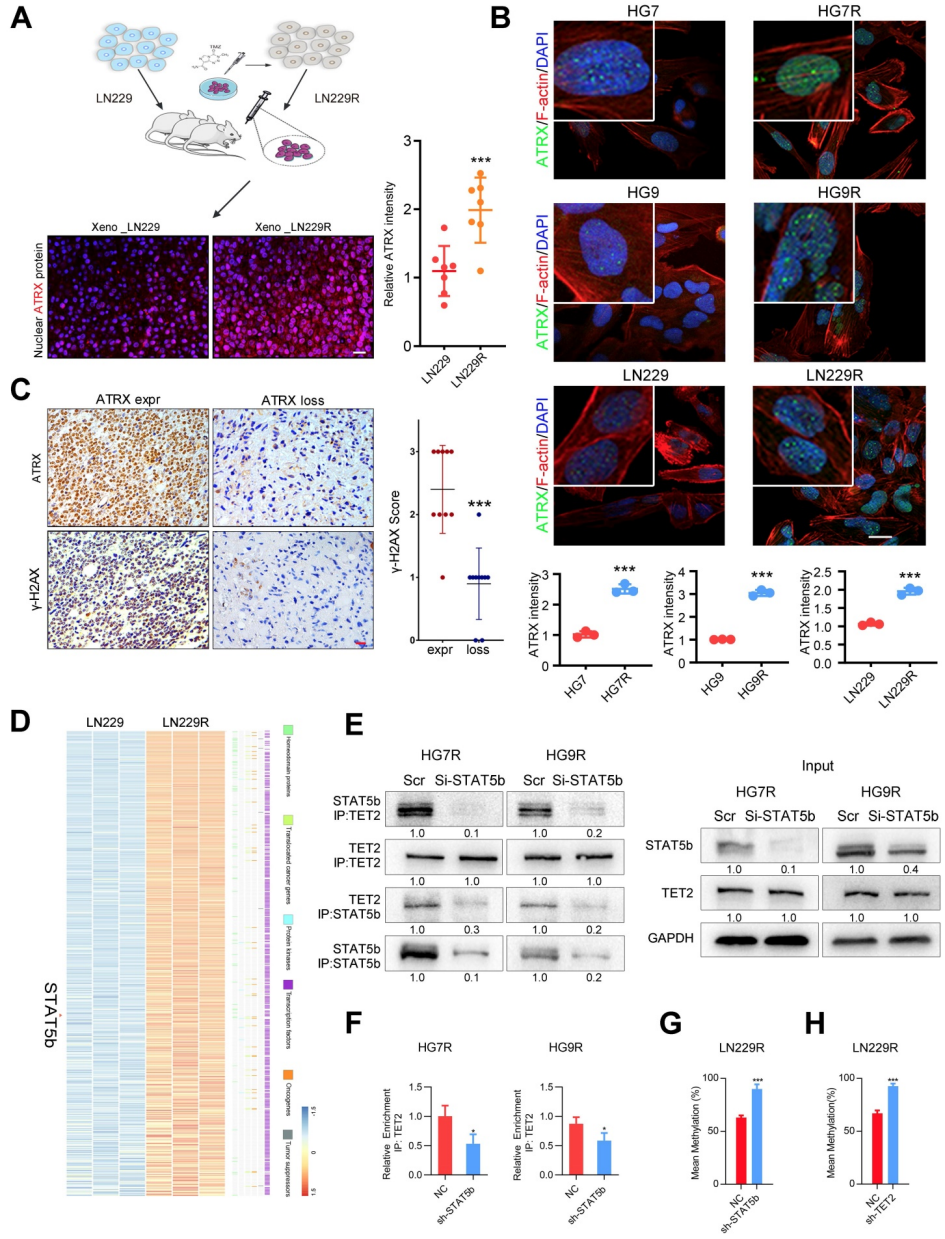
** ATRX expression is increased via DNA demethylation mediated by STAT5b/TET2 complex.** (A) ATRX expression of xenograft gliomas formed by LN229R and LN229 cells. Scale: 20 μm. Schematic used elements from Servier Medical Art: https://smart.servier.com. (B) ATRX expression in HG7, HG9, LN229, HG7R, HG9R and LN229R detected by IF. Scale: 20 μm. (C) Expression of γ-H2AX in ATRX express and ATRX loss glioma tissues. Scale: 20 μm. ***P < 0.001, Chi-squared test. (D) Heatmap with the differentially expressed genes annotated with six gene families. (E) Co-IP assay of STAT5b and TET2 in HG7R and HG9R cells treated with STAT5b knockdown. (F) ChIP-PCR assay showing the TET2 enrichment in ATRX promoter regions of HG7R and HG9R cells treated with STAT5b siRNAs. Error bars indicate mean ± SD. (G-H) The average levels of methylation in ATRX promoter between STAT5b/TET2 knockdown LN229R group and NC group. ***P < 0.001, Student's *t*-test.

We obtained the glioma xenografts 23 formed by TMZ-resistant LN229R cells and observed that ATRX expression increased in tumor tissues from LN229R cells compared with those from parental cells (**Figure 1A**). To further explore the effect of ATRX on TMZ resistance in glioma cells, we further established TMZ resistant GBM cells (HG7R and HG9R, **Figure S1A**) and confirmed that ATRX protein expression was elevated in HG7R and HG9R cells compared to parental cells using IF assay (**Figure 1B**). As detected by IHC assay in 30 GBM tissues from patients, γ-H2AX, an indicator of DNA damage repair 28, was reduced in ATRX-loss tissues compared with ATRX-expressive tissues (**Figure 1C**). To elucidate the mechanism underlying ATRX up-regulation, we conducted gene expression profiling analysis to compare mRNA expression profiles between LN229R and parental cells. The differentially expressed mRNAs (P < 0.05, **Table S4**) were annotated with six gene families obtained from the Molecular Signature Database. The expression of signal transducer and activator of transcription 5B (*STAT5b*), a transcription factor that promotes the malignant progression of gliomas 29, was abnormally upregulated in TMZ resistant cells (**Figure 1D**). STAT5b ChIP-seq analysis and ChIP-PCR assays revealed that STAT5b had wide-spread binding to the genome (**Figure S1B**) and was highly enriched in *ATRX* promoter region of HG7R and HG9R cells (**Figure S1C-D**). The knockdown of *STAT5b* abolished ATRX expression in HG7R and HG9R cells (**Figure S1E-G**). However, we observed no induction of luciferase activity in STAT5b binding fragment within *ATRX* promoter region between *STAT5b* knockdown and NC cells through luciferase reporter assay (**Figure S1H**).

Aberrant DNA methylation also plays a critical role in the chemoresistance of cancer 30. A previous study illustrated that STAT5b could recruit tet methylcytosine dioxygenase 2 (TET2), a methylcytosine dioxygenase that promoted DNA demethylation and upregulated gene expression 31. We found a significantly positive correlation between *STAT5b/TET2* and *ATRX* expression in public data sets (**Figure S2A**). DNA methylation microarray analysis demonstrated that LN229R cells had lower methylation levels in *ATRX* promoter region compared to parental cells (**Figure S2B-C**). The pyrosequencing assay further confirmed demethylation in *ATRX* promoter region in TMZ resistant cells compared to parental cells (**Figure S2D**) and knockdown of *TET2* also reduced ATRX expression in HG7R and HG9R cells (**Figure S3A-C**). ChIP-seq analysis and ChIP-PCR assays indicated that there was higher enrichment of TET2 in *ATRX* promoter region of HG7R and HG9R cells compared with parental cells (**Figure S3D-F**). The co-IP assay revealed that STAT5b knockdown reduced the immunoprecipitated TET2 compared to negative control group (**Figure 1E**). TET2 enrichment in *ATRX* promoter region was significantly reduced after *STAT5b* knockdown in HG7R and HG9R cells (**Figure 1F**). Furthermore, we found that *STAT5b* and* TET2* knockdown by lentivirus suppressed the TET2-mediated ATRX promoter demethylation in LN229R cells (**Figure 1G-H**, **Figure S2E-F**). These results indicated that the upregulation of ATRX was mediated via aberrant DNA methylation induced by STAT5b/TET2 complex in TMZ resistant cells.

### ATRX strengthens DNA damage repair by stabilizing PARP1 protein in TMZ resistant cells

**Figure 2:**
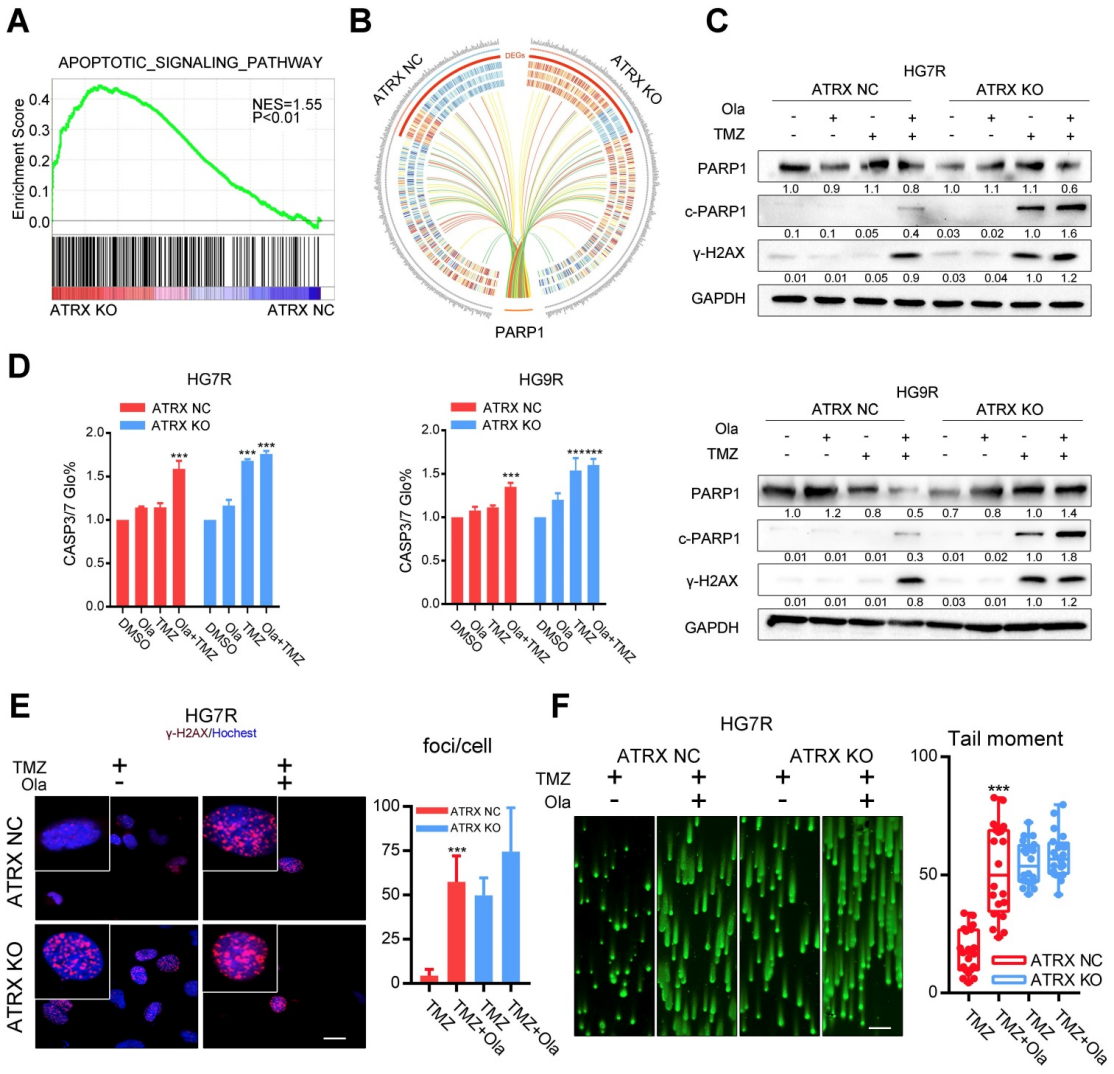
** Olaparib increases DNA damage induced by TMZ by inhibiting the ATRX mediated PARP1 stabilization.** (A) GSEA of the apoptotic signaling pathway was performed in *ATRX* NC and *ATRX* KO cells. (B) Differentially expressed genes of the apoptotic signaling pathway between *ATRX* NC and *ATRX* KO cells and their interaction with PARP1. (C) Western blotting of PARP1, c-PARP1 and γ-H2AX of *ATRX* NC and *ATRX* KO cells treated with 200 μM TMZ and/or 1 μM olaparib. TMZ and olaparib were added to culture medium for 72 hours. (D) CASP3/7 activity of *ATRX* NC and *ATRX* KO cells treated with TMZ and/or olaparib. TMZ and olaparib were added to culture medium for 72 hours. (E) TMZ and olaparib were added to culture medium at final concentrations of 200 μM and 1 μM, respectively, for 72 hours. γ-H2AX is shown in red and nucleus in blue. Bar plots showed the statistics of immunofluorescence assays in HG7R. Scales: 20 μm. (F) Comet assays measuring the DNA damage status in *ATRX* NC and *ATRX* KO cells treated with TMZ or combination of TMZ and olaparib in HG7R. Scales: 100 μm. Error bars indicate mean ± SD. *** P < 0.001, Student's *t*-test.

To evaluate the impact of ATRX contributing to TMZ resistance, *ATRX* knockout cells were established by CRISPR-Cas9 gene editing technology in TMZ resistant GBM cells (LN229R, HG7R and HG9R, **Figure S4A**). Gene expression profiling analysis was carried out to detect mRNA expression alterations between control and *ATRX* knockout cells. According to the GSEA, the upregulated genes were implicated in apoptotic signaling pathway (**Figure 2A**, NES = 1.55, P < 0.001) and (NAD ADP ribosyltransferase activity **Figure S4B**, NES = 1.57, P< 0.001). Functional protein association network analysis revealed that 13 proteins encoded by the differentially expressed genes (P < 0.05) between control and *ATRX* knockout cells had interaction with PARP1 (**Figure 2B**, **Table S2**), an enzyme involved in DNA repair 12. *ATRX* knockout cells showed more cleaved PARP1, an inactivated form of PARP1 32, and increased γ-H2AX level induced by TMZ compared with *ATRX* NC cells (**Figure 2C**, **E** and **Figure S4C**). Increased activity of CASP3/7, the protein markers of apoptosis, was induced by TMZ in *ATRX* knockout cells, and TMZ combined with olaparib could increase CASP3/7 activity in *ATRX* NC cells (**Figure 2D**). Western blotting confirmed that in HG7R and HG9R cells, *ATRX* knockout or olaparib increased the activation of CASP8 and CASP3, and degradation of BCL2 and BCL-XL induced by TMZ (**Figure S4E**). Furthermore, inhibition of PARP1 induced by olaparib restored the induction of DNA damage and inhibition of cell migration ability, vascular mimicry ability and cell growth caused by TMZ in *ATRX* NC cells. However, there was no significant increase of TMZ sensitivity in *ATRX* KO cells treated by olaparib compared with those treated with TMZ alone (**Figure 2F**, **Figure S4D** and **Figure S5**). These results indicate that ATRX strengthens DNA damage repair via stabilizing PARP1 protein to promote TMZ resistance in glioma cells.

### ATRX elicits PARP1 stabilization through down-regulating FADD expression in TMZ resistant cells

**Figure 3:**
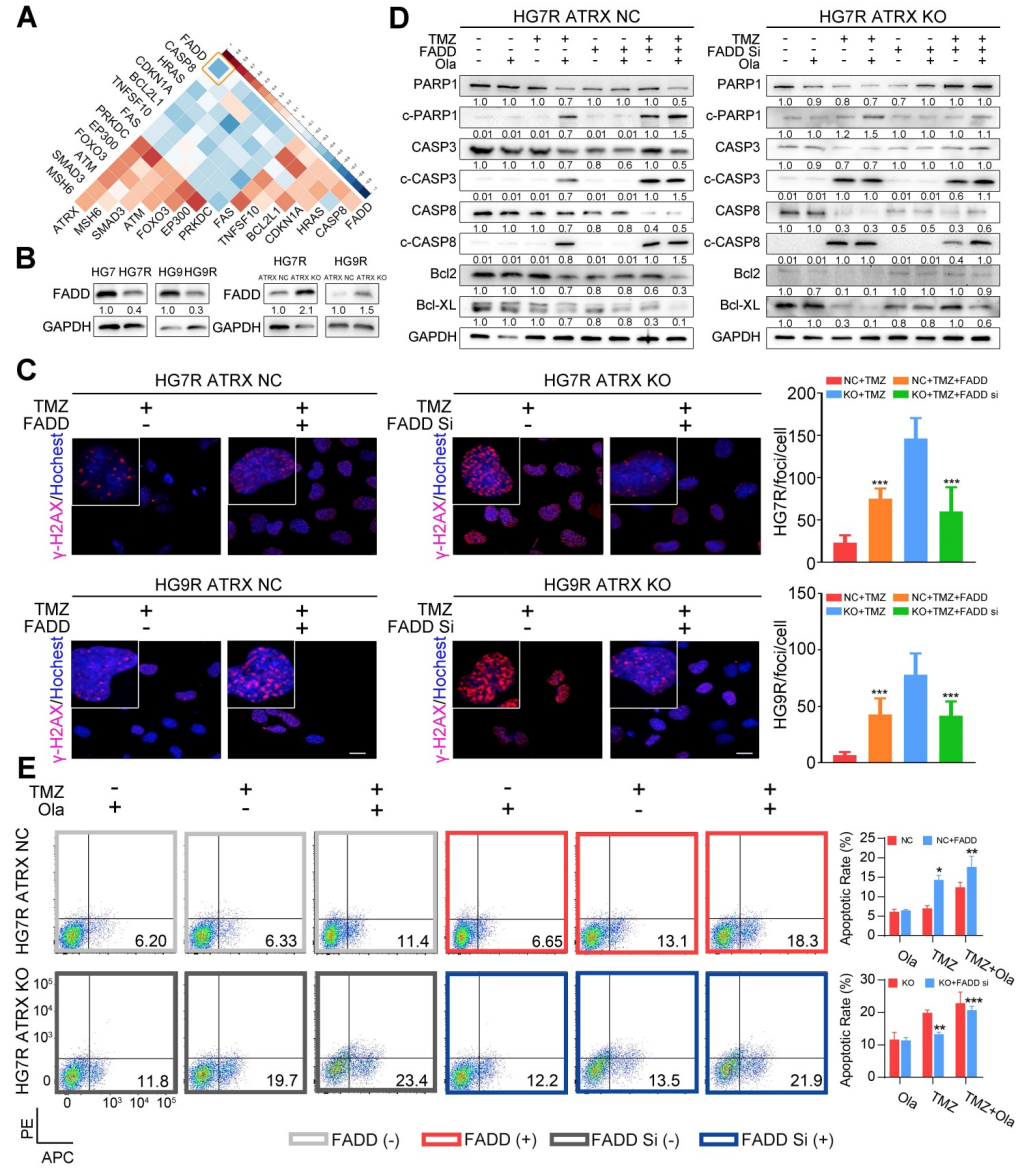
** FADD mediated by ATRX inhibits PARP1 stabilization and strengthen DNA damage and apoptosis induced by TMZ.** (A) Correlations among *ATRX* and thirteen apoptotic molecules interacting with PARP1 in TCGA dataset. (B) Expression level of FADD in HG7, HG7R, HG9, HG9R, *ATRX* NC and *ATRX* KO cells. (C) IF assays showing γ-H2AX levels (γ-H2AX in red and nucleus in blue) of *ATRX* NC and *ATRX* KO cells with FADD overexpression or knockdown. Scale: 20 μm. The statistic of IF assays are shown in bar plots. (D) Expression levels of PARP1, cPARP1, CASP8, cCASP8, CASP3, cCASP3, BCL-XL and BCL2 detected by Western blot in *ATRX* NC and *ATRX* KO cells with FADD overexpression or knockdown treated with 200 μM TMZ and/or 1 μM olaparib. (E) Apoptosis of HG7R NC and HG7R KO cells with FADD overexpression or knockdown treated with 200 μM TMZ and/or 1μM olaparib for 72 hours. Error bars indicated mean ± SD. *P < 0.05, **P < 0.01, ***P < 0.001, Student's t-test.

Based on the above results that 13 of the proteins encoded by the differentially expressed genes between control and ATRX knockout cells had interaction with PARP1, we sought to further investigate the mechanism of the observed ATRX knockout mediated suppression of PARP1 in TMZ resistant cells. In the TCGA dataset, *FADD* had the most significantly negative correlation with *ATRX* in GBM samples (R = -0.47, P < 0.0001, **Figure 3A**, **Table S6**). To evaluate whether ATRX mediated TMZ resistance was dependent on FADD expression, we overexpressed FADD in *ATRX* NC cells and knocked down FADD in *ATRX* KO cells (**Figure S6A**, **Figure S6B**). Decreased FADD was detected in HG7R and HG9R cells compared to parental cells, and we also observed up-regulated FADD expression in *ATRX* knockout cells (**Figure 3B**). Overexpressed FADD increased TMZ induced DNA damage in *ATRX* NC cells. Conversely, knockdown of FADD decreased TMZ induced DNA damage in *ATRX* knockout cells (**Figure 3C**).

**Figure 4:**
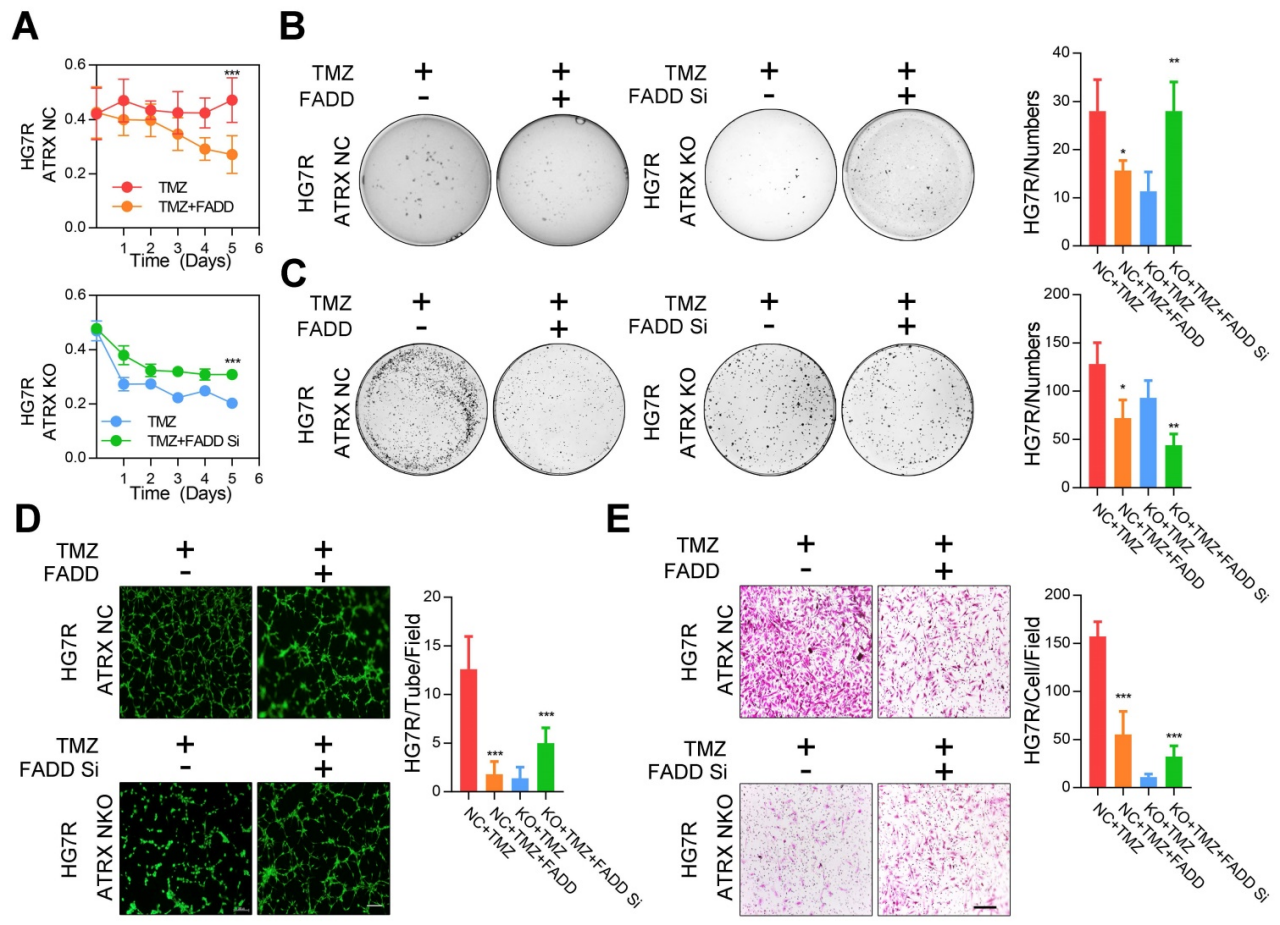
** The proliferative, invasive ability and vascular mimicry of *ATRX* NC and *ATRX* KO HG7R cells with *FADD* overexpression or knockdown treated with TMZ.** (A) CCK-8 assays in *ATRX* NC and *ATRX* KO HG7R cells with *FADD* overexpression or knockdown treated with 200μM TMZ. (B-C) Soft agar colony formation assays, colony formation assays in *ATRX* NC and *ATRX* KO HG7R cells with *FADD* overexpression or knockdown treated with 200μM TMZ. (D) Vascular mimicry assays in *ATRX* NC and *ATRX* KO HG7R cells with *FADD* overexpression or knockdown treated with 200μM TMZ. (E) Transwell assays of *ATRX* NC and *ATRX* KO HG7R cells with *FADD* overexpression or knockdown accompanied by TMZ. Cells were treated with TMZ at a final concentration of 200 μM. Scale: 200 μm. The statistics was showed in bar plots. Error bars indicated mean ± SD. * P < 0.05, ** P < 0.01, *** P < 0.001; Student's *t*-test.

Consistent with the effect of olaparib, overexpression of FADD, increased TMZ induced PARP1 cleavage, activation of CASP8 and CASP3, degradation of BCL2 and BCL-XL, and promoted cell apoptosis were observed in ATRX NC cells. Knockdown of FADD expression, on the other hand, decreased TMZ induced PARP1 cleavage, activation of CASP8 and CASP3, degradation of BCL2 and BCL-XL, and suppressed cell apoptosis in *ATRX* knockout cells whereas olaparib could restore TMZ induced effects after FADD knockdown in *ATRX* knockout cells (**Figure 3D-E** and **Figure S6C-D**). Overexpressed FADD increased TMZ induced inhibition of cell viability, invasiveness and vascular mimetic ability of *ATRX* NC cells, whereas knockdown of FADD had opposite effects on *ATRX* knockout cells (**Figure 4** and **Figure S7**). Taken together, these results indicate that the suppression of FADD is responsible for ATRX mediated PARP1 stabilization and TMZ resistance in glioma cells.

### FADD is suppressed by H3K27me3 enrichment depended on ATRX/EZH2 complex in TMZ resistant cells

**Figure 5:**
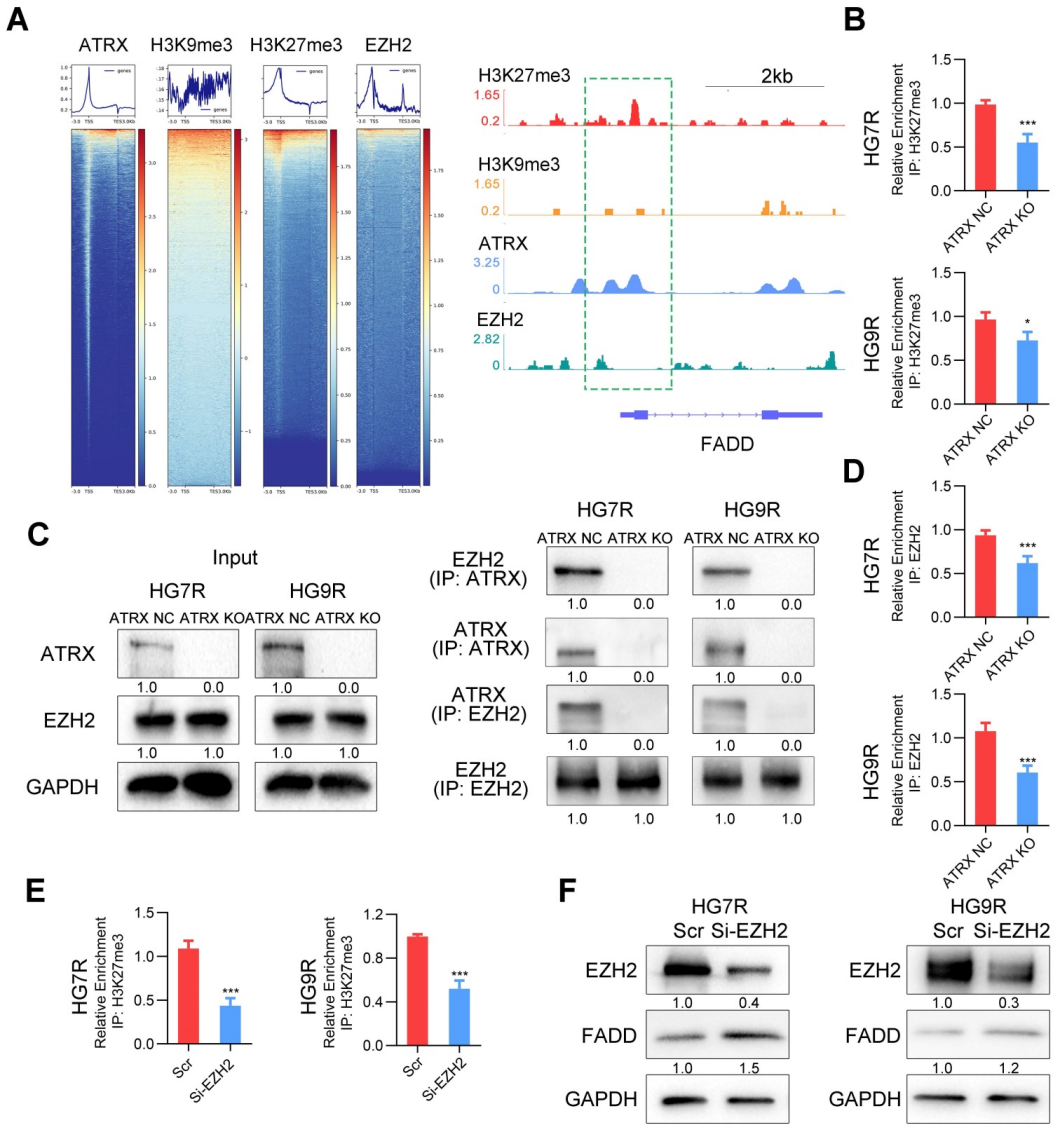
** FADD is suppressed by H3K27me3 ATRX/EZH2 complex mediated H3K27me3 enrichment in HG7R and HG9R cells.** (A) Heatmap showing the read counts within a region spanning ± 3 kb around TSS. The IGV browser image of H3K27me3, H3K9me3, ATRX and EZH2 enrichment in *FADD* promoter region. (B) ChIP-PCR analysis of *FADD* promoter regions with antibodies targeting H3K27me3 in *ATRX* NC and KO cells. (C) Co-IP assay of the interaction between ATRX and EZH2 in *ATRX* NC and KO cells. (D) ChIP-PCR analysis of *FADD* promoter regions with antibodies targeting EZH2 in *ATRX* NC and KO cells. (E) ChIP-PCR analysis of *FADD* promoter regions with antibodies targeting H3K27me3 in HG7R and HG9R cells with EZH2 knockdown (EZH2 si3 was used in the assay). (F) FADD expression was detected by Western blotting in HG7R and HG9R cells treated with EZH2 knockdown.

Our previous study demonstrated that ATRX was involved in the modification of histone H3 by recruiting histone methyltransferase 11. To confirm whether ATRX suppressed FADD expression by histone modification, we analyzed the enrichment of ATRX, and repressive histone modifications H3K9me3 and H3K27me3 in *FADD* promoter region by ChIP-seq analysis. According to IGV plots, ATRX could bind to *FADD* promoter region and there was more H3K27me3 enrichment in *FADD* promoter region than H3K9me3, indicating that H3K27me3 was the dominant histone modification in regulating *FADD* expression. ChIP-seq analysis of enhancer of zeste homolog 2 (EZH2), a methyltransferase of H3K27me3^33^, illustrated that there was an enrichment peak of EZH2 in the promoter region of FADD (**Figure 5A**). ChIP assays revealed that *ATRX* knockout suppressed the enrichment of H3K27me3 in *FADD* promoter region in HG7R and HG9R cells (**Figure 5B**), and that there was more enrichment of H3K27me3 in *FADD* promoter region of HG7R and HG9R cells than that of parental cells (**Figure S8A**). Protein binding prediction analysis suggested ATRX could potentially bind to EZH2 (**Figure S8B**). Co-IP assays showed ATRX binding EZH2 in* ATRX* NC cells while this interaction could not be detected in* ATRX* knockout cells (**Figure 5C**). ChIP assays illustrated that *ATRX* knockout suppressed the enrichment of EZH2 in *FADD* promoter region in HG7R and HG9R cells (**Figure 5D**), and that there was more enrichment of EZH2 in *FADD* promoter region of HG7R and HG9R cells than that of parental cells (**Figure S8C**). Knockdown of *EZH2* with specific siRNAs suppressed trimethylation of H3K27 enrichment on the *FADD* promoter region and released FADD expression in HG7R and HG9R cells (**Figure 5E-F** and **Figure S8D**), suggesting that ATRX/EZH2 complex promoted trimethylation of histone H3K27 in *FADD* promoter region and suppressed its expression in TMZ resistant cells.

### Concomitant PARPi with TMZ inhibits glioma growth in TMZ resistant xenograft models

**Figure 6:**
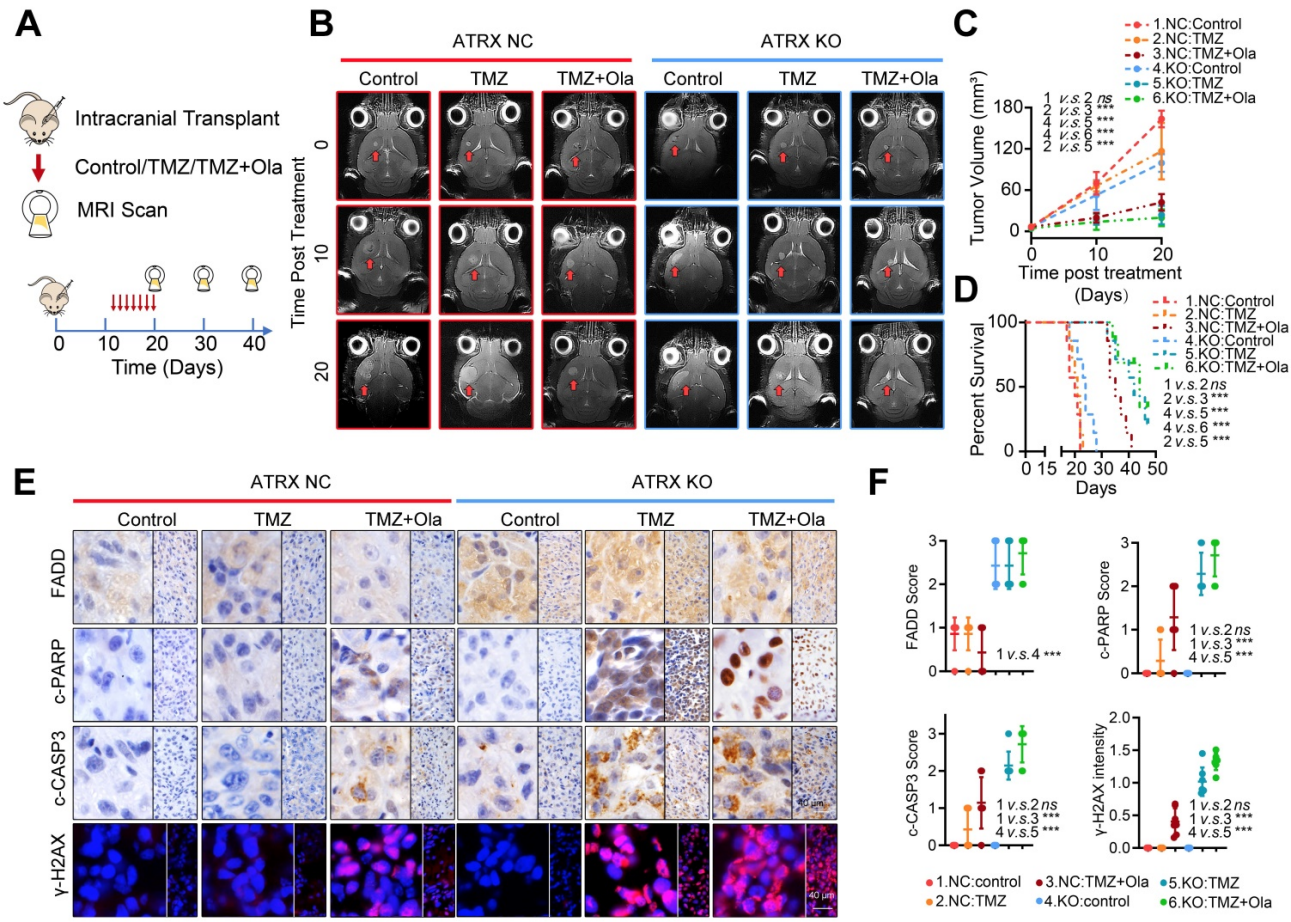
** Concomitant PARPi with TMZ inhibits glioma growth in TMZ resistant xenograft models.** (A-B) The MRI images of *ATRX* NC and *ATRX* KO xenograft gliomas treated with control, TMZ or a combination of TMZ and olaparib (*n* = 7 per group). (C-D) Tumor volumes and the survival curves of nude mice in different groups. *** P < 0.001 and “ns” means not significant; Student's t-test. (E) The FADD, c-CASP3 and c-PARP1 levels of xenograft gliomas in different groups detected by IHC assays. Scale: 40 μm. The γ-H2AX expression level detected by IF. Scale: 40 μm. (F) IHC staining quantification of FADD, c-CASP3, c-PARP and γ-H2AX. *** P < 0.001 “*ns*” not significant, Chi-squared test.

To further validate the fact that PARP inhibitor enhanced TMZ sensitivity by suppressing ATRX-mediated PARP1 stabilization in TMZ resistant gliomas, we established xenograft mouse models and treated them with TMZ and (or) olaparib. We found that TMZ had little effect on the suppression of tumor growth in the HG7R NC group, but TMZ combined with olaparib significantly inhibited the tumor growth in mice in the HG7R NC group, consistent with the enhanced TMZ sensitivity induced by ATRX knockout in HG7R KO group (**Figure 6A-B**). Compared with the mice in the HG7R NC group treated with TMZ alone, those treated with the combination of TMZ and olaparib had a prolonged survival time, similar to those in the HG7R KO group treated with TMZ and (or) olaparib (**Figure 6C-D**). The IHC staining of xenograft samples showed that there was a higher level of FADD expression in the HG7R KO group than in the HG7R NC group. Compared with the HG7R NC groups treated with TMZ alone, the group treated with TMZ combined with olaparib displayed increased γ-H2AX, activated CASP3 and cleaved PARP1, similar to the HG7R KO group treated with TMZ and (or) olaparib (**Figure 6E-F**). The results indicated that in TMZ-resistant gliomas, a combination of PARP inhibitor with TMZ inhibited tumor growth by suppressing ATRX mediated PARP1 stabilization.

## Discussion

Chemoresistance is a major issue in the management of patients with GBM. DNA damage repair mechanisms hamper the cytotoxic effect of the DNA damaging chemotherapeutic agent TMZ in GBM 34. Repair of TMZ induced DNA double-strand breaks (DSBs) can be achieved by two major pathways, homologous recombination (HR) and non-homologous end joining (NHEJ) DNA repair 35. DNA lesions are recognized and bound by poly (ADP-ribose) polymerase family members such as PARP1 and other scaffold proteins to inhibit TMZ induced cell cycle arrest and apoptosis in GBM cells 36. Considerable effort is being devoted to identifying the molecular basis of TMZ resistance in GBM and exploring novel therapeutic regimens that may improve overall survival 37-39.

Mutations of ATRX, accompanied by loss of the nuclear protein, constitute the most frequent genetic alterations in gliomas 16. Our previous work demonstrated that ATRX is involved in the DNA replication process, cell cycle regulation, and DNA damage repair in gliomas 11. ATRX depletion inhibits cell growth and invasion, reduced transcriptional activity, and increases sensitivity to TMZ in glioma cells 40. In the present study, we observed that there was a higher expression of ATRX in TMZ resistant glioma cells compared to parental cells, and that ATRX was positively correlated with DNA damage repair marker γ-H2AX in GBM specimens. Also, aberrant activation of receptor tyrosine kinase (RTK) pathways contributes to TMZ resistance via overexpression or activation of downstream transcription factors leading to the unfavorable outcome of patients with GBM 41, 42. We found that the expression of *STAT5b*, a transcription factor that could be phosphorylated in EGF or HGF dependent manner and promoted the malignant progression of gliomas 29, 43, was abnormally upregulated in TMZ resistant cells. High throughput DNA methylation microarray showed that *ATRX* promoter region had a lower methylation level in TMZ resistant cells compared to parental cells, suggesting that DNA demethylation might mediate ATRX expression in TMZ resistant glioma cells. TET2, as an interaction partner of the transcription factor EBF1, regulates DNA methylation in gliomas 44, STAT5b also could recruit TET2 in specific site to promote DNA demethylation 45. With a decreased expression of STAT5b or TET2, methylation in ATRX promoter region was upregulated. Knockdown of *STAT5b* reduced TET2 enrichment in *ATRX* promoter region in TMZ resistant cells, suggesting a novel specific mechanism for regulating ATRX expression via DNA demethylation induced by STAT5b/TET2 complex.

Differential gene expression analysis revealed that ATRX might be implicated in the apoptosis signaling pathway and NAD ADP ribosyltransferase (ADPRT, also named as PARP1) activity in glioma cells. Knockout of ATRX enhanced PARP1 cleavage and elevated TMZ sensitivity in TMZ resistant GBM cells. Recent evidence has implicated that PARP1 was involved in DNA repair pathways and maintenance of genomic stability by increasing the resistance of cells to DNA damaging agents, possibly by promoting DNA strand break rejoining 46. Olaparib, a PARP inhibitor, was able to penetrate both core and margins of recurrent glioblastoma and the combination with TMZ significantly increased progression-free survival rates 47. These results demonstrated that olaparib has a promising future in treating glioma patients. In our work, we found that TMZ combined with olaparib significantly restrained growth of ATRX NC TMZ resistant glioma cells in vivo. FADD, a pro-apoptosis factor 48 that participates in the negative control of PARP1^49^, is upregulated when ATRX is knocked out in TMZ resistant cells. Based on the TCGA dataset analysis, *ATRX* expression is negatively correlate with *FADD* expression. FADD is responsible for Caspase-8 activation and apical caspase processing is followed by activation of BID, processing of Caspase-9, -3 and -7, PARP cleavage and apoptotic cell death. Our results showed overexpression of FADD, which was consistent with the effect of PARP inhibitor olaparib, increased TMZ induced PARP1 cleavage, activation of CASP8 and CASP3, and degradation of BCL2 and BCL-XL in ATRX NC cells. Knockdown of FADD expression decreased TMZ induced PARP1 cleavage, activation of CASP8 and CASP3, and degradation of BCL2 and BCL-XL in ATRX knockout cells, indicating that suppression of FADD is responsible for ATRX mediated PARP1 stabilization and TMZ resistance in glioma cells.

ATRX can modify the histone variant composition of chromatin that might regulate genomic stability or gene expression changes by recruiting other partners 50. In our previous study, we found that ATRX could bind to modification enzymes and facilitated maintenance of H3K9me3 to regulate activation of the downstream signaling pathway 9. As repressive markers, H3K9me3 and H3K27me3 lead to the transcriptional suppression of target genes and their abundance in chromatin is frequently altered in cancer 51. We carried out the ChIP-seq analysis of H3K9me3 and H3K27me3 and observed enrichment of H3K27me3 in *FADD* promoter regions. Protein prediction analysis and co-IP assay verified that EZH2, the PRC2 subunit that catalyzes the trimethylation of H3K27, could bind to ATRX, consistent with the previous study 52. Loss of ATRX causes global shifts in PRC2 localization and function, its redistribution within coding genes and decreased selectivity of H3K27 methylation 53. Our results demonstrated that *ATRX* knockout suppressed the enrichment of EZH2 and H3K27me3 on *FADD* promoter region, and that knockdown of EZH2 inhibited the trimethylation of H3K27 enrichment of *FADD* promoter region and released FADD expression in TMZ resistant cells. The IHC staining of tumor slides from xenograft models also confirmed a higher level of FADD expression in ATRX KO group compared to the ATRX NC group. Also, TMZ combined with PARP1 inhibitor olaparib significantly inhibited tumor growth in mice in the HG7R NC group, which was consistent with the enhanced TMZ sensitivity induced by ATRX knockout in the HG7R KO group.

## Conclusions

**Figure 7:**
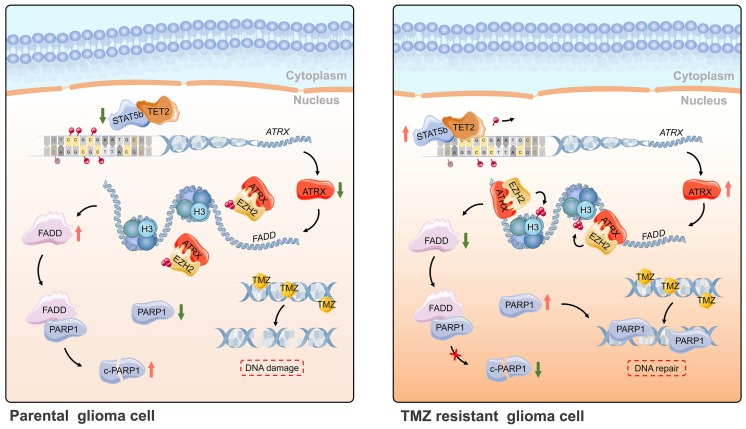
** Schematic of epigenetic modulation of ATRX by STAT5b/TET2 complex regulating the FADD/PARP1 axis and contributing to TMZ resistance in glioma.** Schematic used elements from Servier Medical Art: https://smart.servier.com.

In summary, ATRX was upregulated by DNA demethylation mediated by STAT5b/TET2 complex in TMZ resistant glioma cells. ATRX also promoted PARP1 stabilization through down-regulating FADD expression by the suppressing H3K27me3 enrichment in FADD promoter region. These results further illustrated the mechanism of the ATRX/PARP1 axis contributing to TMZ resistance (**Figure 7**) and provided substantial new evidence that PARP inhibitor might be a potential adjuvant agent in overcoming TMZ resistance in glioma.

## Supplementary Material

Supplementary figures and tables.

### Funding

This study was supported by 1. National Natural Science Foundation of China (No. 81702972, No. 81874204, No. 81903077, No. 81772666, No.81972817); 2. Excellent Young Talents Project of Central Government Supporting Local University Reform and Development Fund (0202-300011190006); 3. China Postdoctoral Science Foundation (2018M640305, 2019M660074); 4. Beijing Postdoctoral Research Foundation (ZZ2019-10); 5. Heilongjiang Postdoctoral Science Foundation (LBH-Z18103, LBH-Z19029); 6. Heilongjiang Health and Family Planning Commission Foundation (2017-201, 2019-102).

## Abbreviations

ATRX: Alpha Thalassemia/Mental Retardation Syndrome X-Linked
Cas9: CRISPR-associated protein 9
CASP3: Caspase 3
CASP7: Caspase 7
CASP8: Caspase 8
c-CASP3: cleaved Caspase 3
c-CASP8: cleaved Caspase 8
CCK-8: Cell Counting Kit-8
Co-IP: co-immunoprecipitation
c-PARP1: cleaved PARP1
CRISPR: Clustered Regularly Interspaced Short Palindromic Repeats
EdU: 5-Ethynyl-2´-deoxyuridine
EZH2: Enhancer of zeste homolog 2
FADD: Fas Associated via Death Domain
FBS: Fetal Bovine Serum
FDA: Food and Drug Administration
GBM: glioblastoma
GEO: the Gene Expression Omnibus
GO: Gene Ontology
GSEA: the Gene Set Enrichment Analysis
H&E: Hematoxylin and Eosin stain
IF: immunofluorescence
IGV: the Integrative Genomics Viewer
IHC: immunohistochemistry
MRI: Magnetic Resonance Imaging
MSigDB: the Molecular Signature Database
NHEJ: Non-Homologous End Joining
PARP1: Poly (ADP-ribose) polymerase 1
sgRNA: small guide RNA
siRNA: small interfering RNA
STAT5b: Signal Transducer and Activator of Transcription 5b
SWI/SNF: SWItch/Sucrose Non-Fermentable
TCGA: The Cancer Genome Atlas
TET2: Tet Methylcytosine Dioxygenase 2
TMZ: temozolomide
TRAIL: TNF-Related Apoptosis-Inducing Ligand
WB: Western blot
γ-H2AX: the phosphorylated form of H2A histone family member X
