# Supplementary material for: ATRX/EZH2 complex epigenetically regulates FADD/PARP1 axis, contributing to TMZ resistance in glioma
Source: Theranostics. 2020 Feb 10;10(7):3351–65. doi: 10.7150/thno.41219 (PMC7053195; doi:10.7150/thno.41219)
Supplement: Supplementary file 1 — Supplementary figures and tables. [file thnov10p3351s1.pdf]

Supplementary material

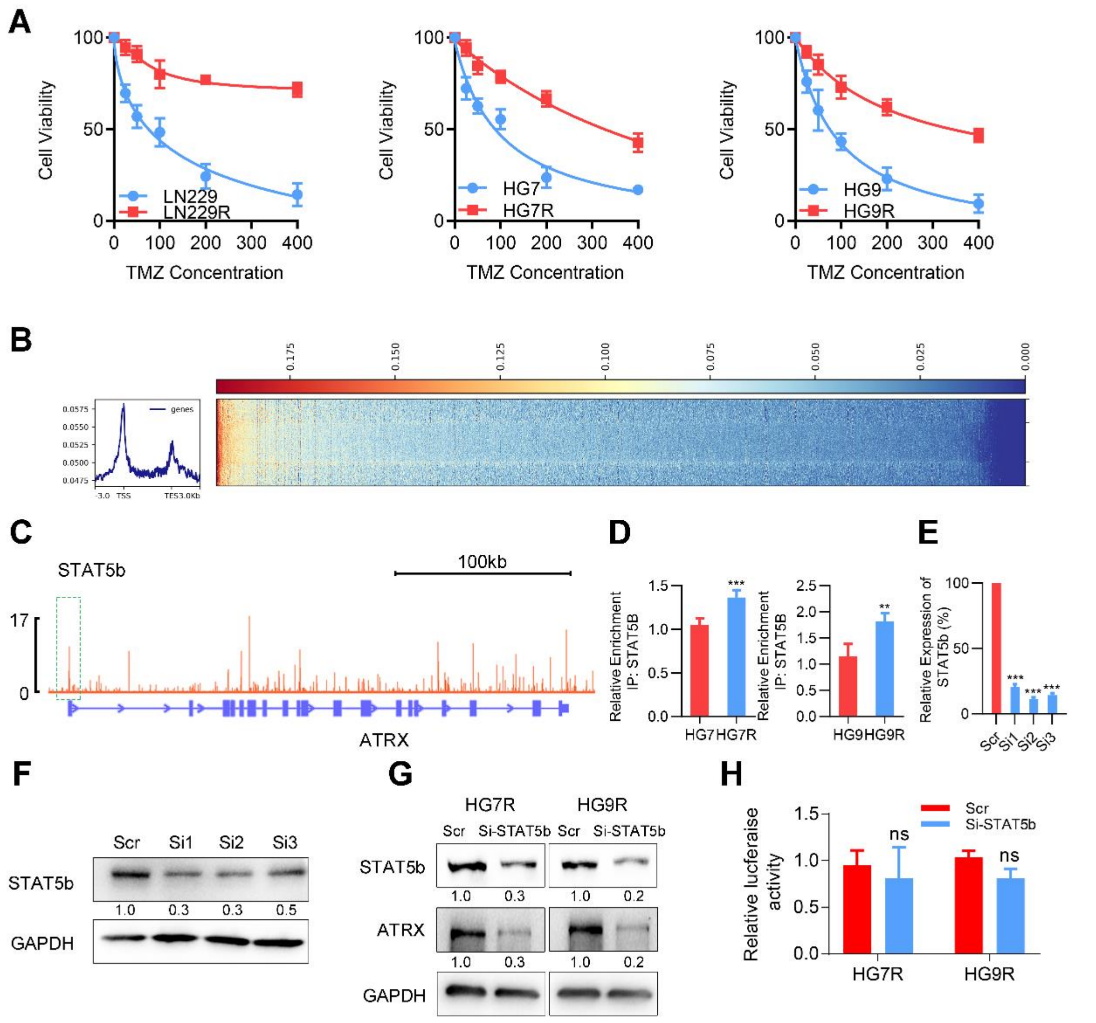

Figure S1. STAT5b is bound to the ATRX promoter region and is involved in ATRX expression.

(A) CCK-8 assays of TMZ-resistant and parental GBM cells upon TMZ treatment at the indicated concentrations for 72 hours (n=3). (B) Heatmap showing the read counts within a region spanning ± 3 kb around TSS in the whole genome. (C) IGV browser image showing STAT5b enrichment in *ATRX* promoter region. (D) ChIP-PCR analysis of *ATRX* promoter regions with STAT5b enrichment. (E-F) Validation of siRNAs targeting STAT5b performed by qRT-PCR and Western blot in HG7R cells. (G) Decreased ATRX protein expression STAT5b knockdown. (H) The transcription activity

of STAT5b in the promoter region of *ATRX* detected by luciferase reporter assay. Error bars indicated mean ± SD. “ns” means no significant, Student’s *t*-test.

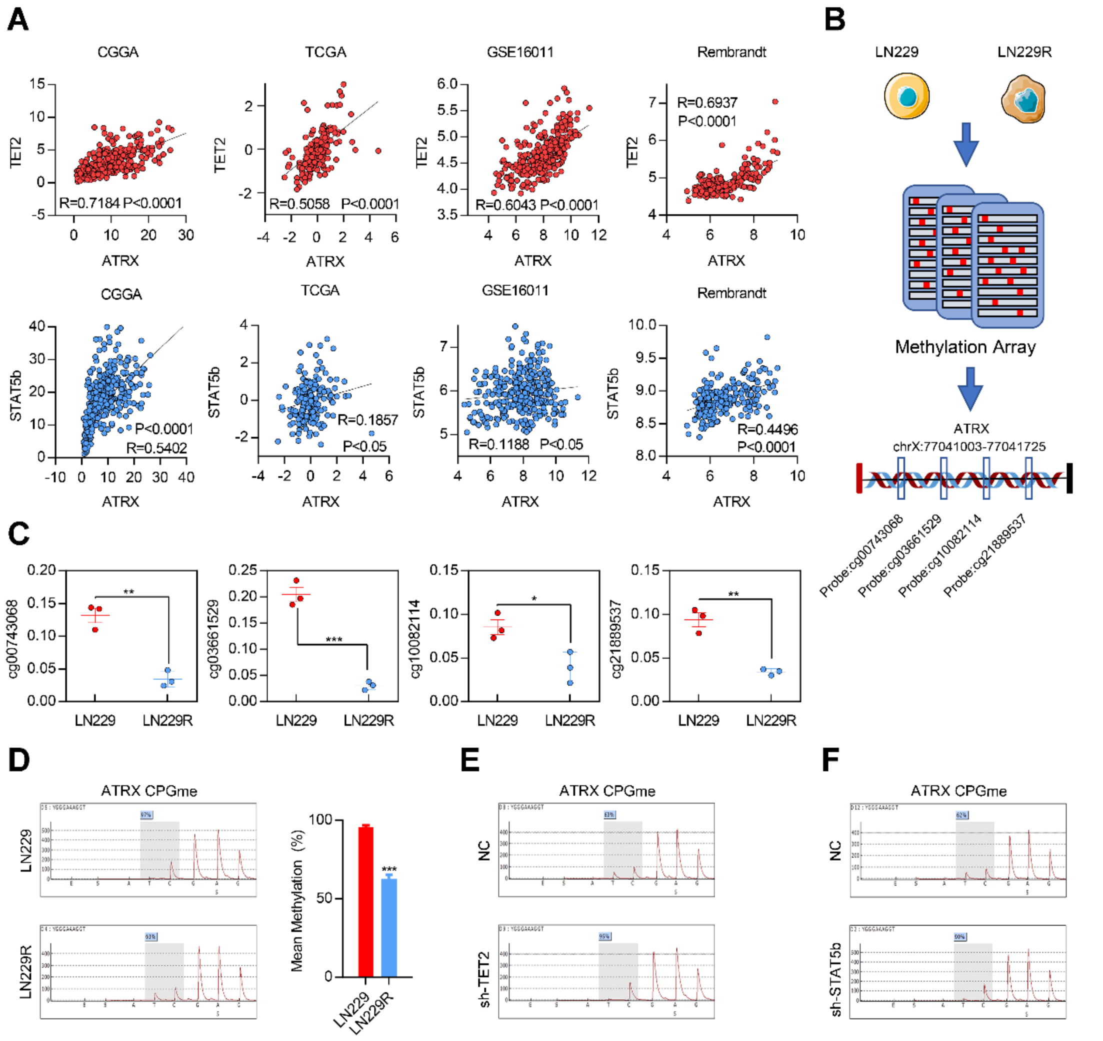

Figure S2. STAT5b/TET2 are response for ATRX hypomethylation

(A) Pearson correlation analysis between the *STAT5b*/*TET2* and ATRX levels in GBM samples (CGGA n=388, TCGA n=160, GSE16011 n=284, Rembrandt n=227). (B) Schematic of the DNA methylation microarray. Schematic used elements from Servier Medical Art: https://smart.servier.com. (C) DNA methylation microarray showing lower methylation in *ATRX* promoter region in LN229R cells compared with parental cells. Error bars indicate mean ± SD. * P < 0.05, ** P < 0.01, *** P < 0.001; Student's *t*-test. (D) Validation of ATRX methylation levels by pyrosequencing in LN229 and 229R cells. (E-F) Validation of ATRX methylation levels by

pyrosequencing in 229R after knocking down *TET2* or *STAT5b*. Error bars indicate mean ± SD. * P < 0.05, ** P < 0.01, *** P < 0.001; Student's *t*-test.

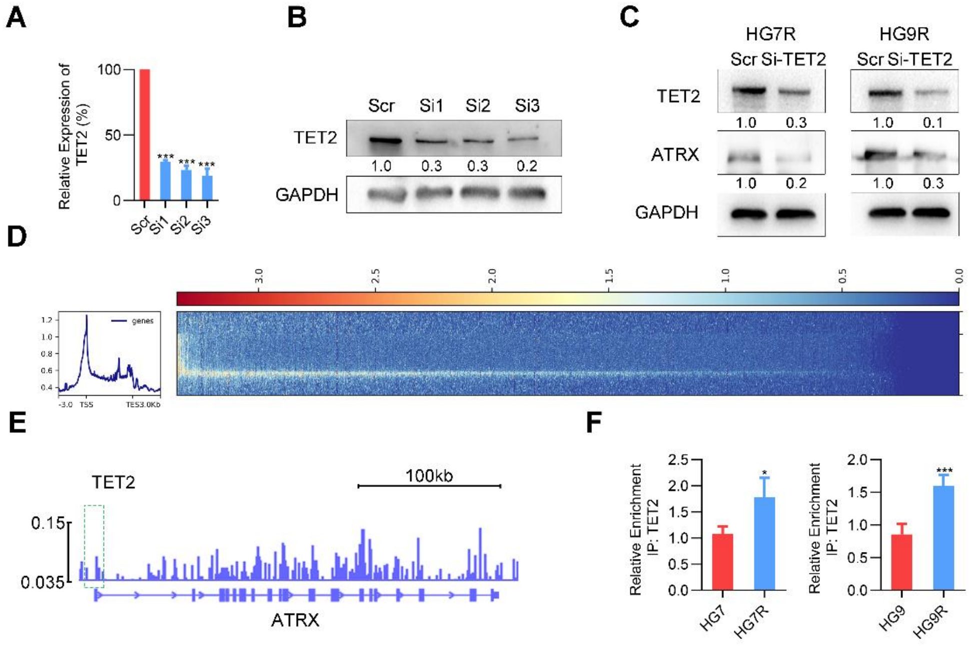

Figure S3. TET2 is enriched in *ATRX* promoter region and involved in ATRX expression. (A-B) Validation of siRNAs targeting TET2 by qRT-PCR and western blotting in HG7R cells. (C) Decreased ATRX protein expression with TET2 knockdown in HG7R and HG9R cells. (D) Heatmap showed the read counts within a region spanning ± 3 kb around TSS in the whole genome. (E) The IGV browser image showing TET2 enrichment in *ATRX* promoter region. (F) ChIP-qPCR analysis of ATRX promoter regions with TET2 enrichment.

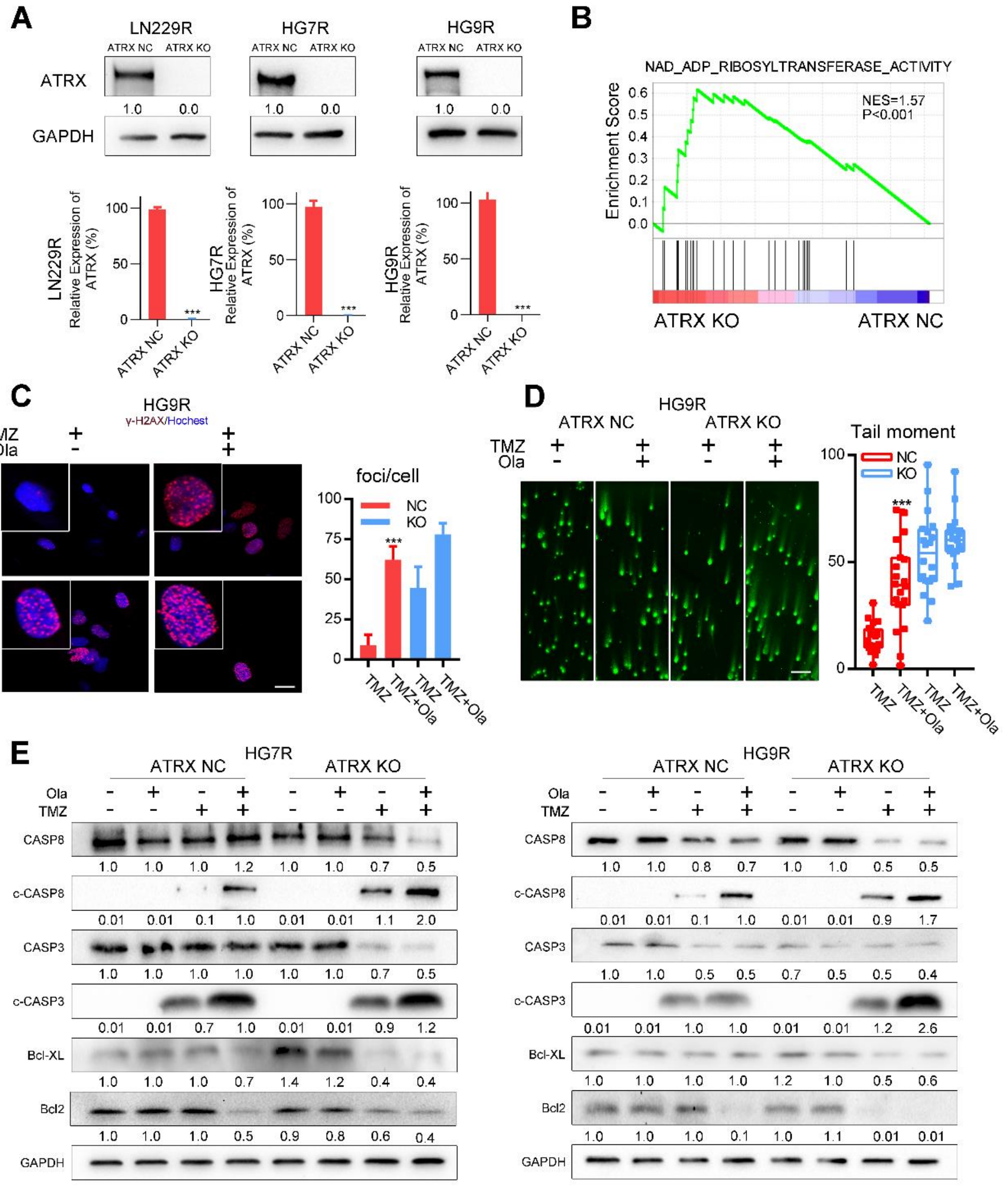

Figure S4. Activity and expression of apoptotic and DNA damage markers in *ATRX* NC and *ATRX* KO cells treated with TMZ and (or) olaparib.

(A) Validation of CRISPR-Cas9 mediated ATRX knocking out performed by qRT-PCR and western blotting in LN229R, HG9R, HG7R cells. (B) GSEA of NAD ADP ribosyltransferase activity pathway was performed between *ATRX* NC and *ATRX* KO cells. (C) γ-H2AX is shown in

red and nucleus in blue. Bar plots showing the statistics of immunofluorescence assays in HG9R. Scales: 20 μm. (D) Comet assays measuring the DNA damage status in *ATRX* NC and *ATRX* KO cells treated with TMZ or combination of TMZ and olaparib in HG9R. Scales: 100μm. Error bars indicate mean ± SD. *** $P < 0.001$, Student's *t*-test. (E) Levels of CASP 8, c-CASP 8, CASP3, c-CASP 3, BCL-XL and BCL 2 detected by western blotting in *ATRX* NC and *ATRX* KO cells treated with TMZ and (or) olaparib.

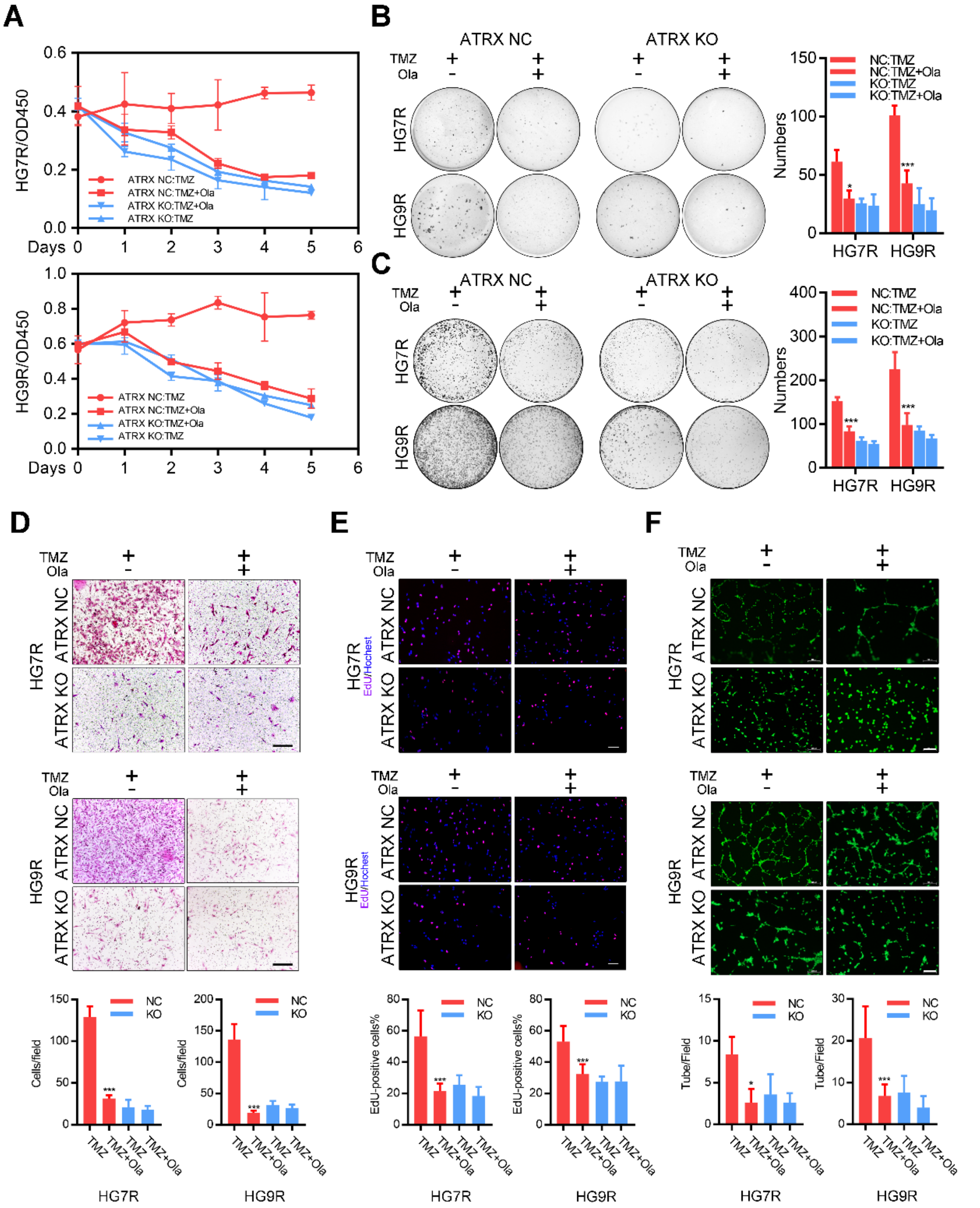

Figure S5. Proliferation, invasive ability and vascular mimicry of *ATRX* NC and *ATRX* KO cells treated with TMZ alone or a combination of TMZ and olaparib.

(A) CCK-8 assays showing the proliferation of *ATRX* NC and *ATRX* KO cells treated with TMZ or a combination of TMZ and olaparib. (B) Soft agar colony formation assay of *ATRX* NC and *ATRX* KO cells treated with TMZ or a combination of TMZ and olaparib. (C) Colony formation assay of *ATRX* NC and *ATRX* KO cells treated with TMZ or a combination of TMZ and olaparib. (D)

Transwell assays of *ATRX* NC and *ATRX* KO HG7R, HG9R cells treated with TMZ or combination of TMZ and olaparib. Scale: 200 µm. (E) EdU assays (EdU in red and nucleus in blue) of *ATRX* NC and *ATRX* KO HG7R, HG9R cells treated with TMZ or a combination of TMZ and olaparib. Scale: 100 µm. (F) Vascular mimicry assays of *ATRX* NC and *ATRX* KO HG7R, HG9R cells treated with TMZ or combination of TMZ and olaparib. Scale: 100 µm. Error bars indicate mean ± SD. * $P < 0.05$, ***$P < 0.001$; Student's *t*-test.

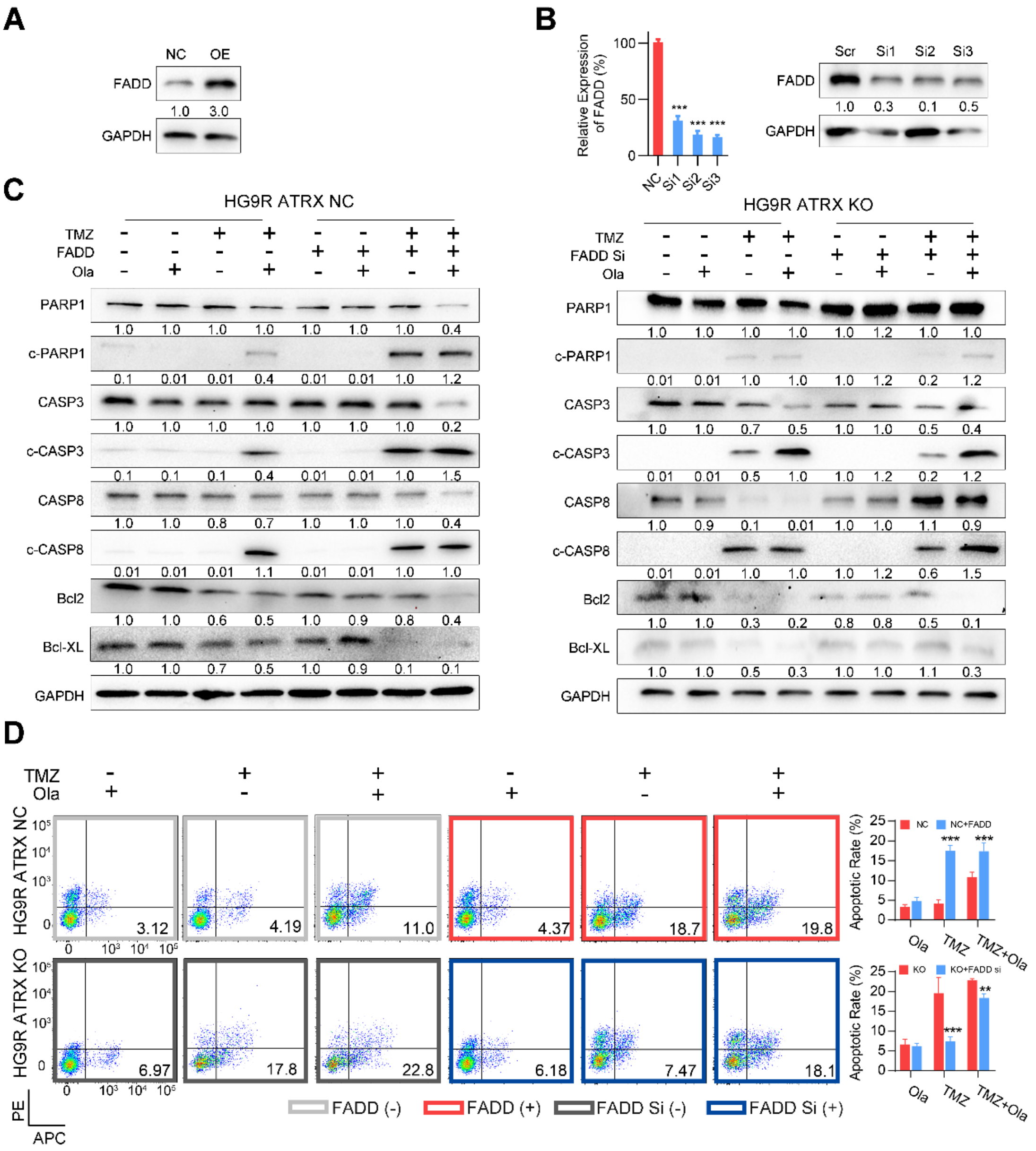

Figure S6. FADD inhibits the stabilization of PARP1 by ATRX and enhances TMZ induced apoptosis.

(A) Validation of FADD overexpression in HG7R cells by Western blotting. (B) Validation of FADD knockdown in HG7R cells. (C) Levels of PARP1, c-PARP1, CASP 8, c-CASP 8, CASP3, c-CASP 3, BCL-XL and BCL 2 detected by Western blotting in *ATRX* NC and *ATRX* KO cells with *FADD* overexpression or knockdown treated with TMZ and (or) olaparib. (D) Cell apoptosis analysis showing the apoptosis of HG9R NC and HG9R KO cells with FADD overexpression or knockdown treated with TMZ and (or) olaparib. **P < 0.01, ***P < 0.001, Student's *t*-test.

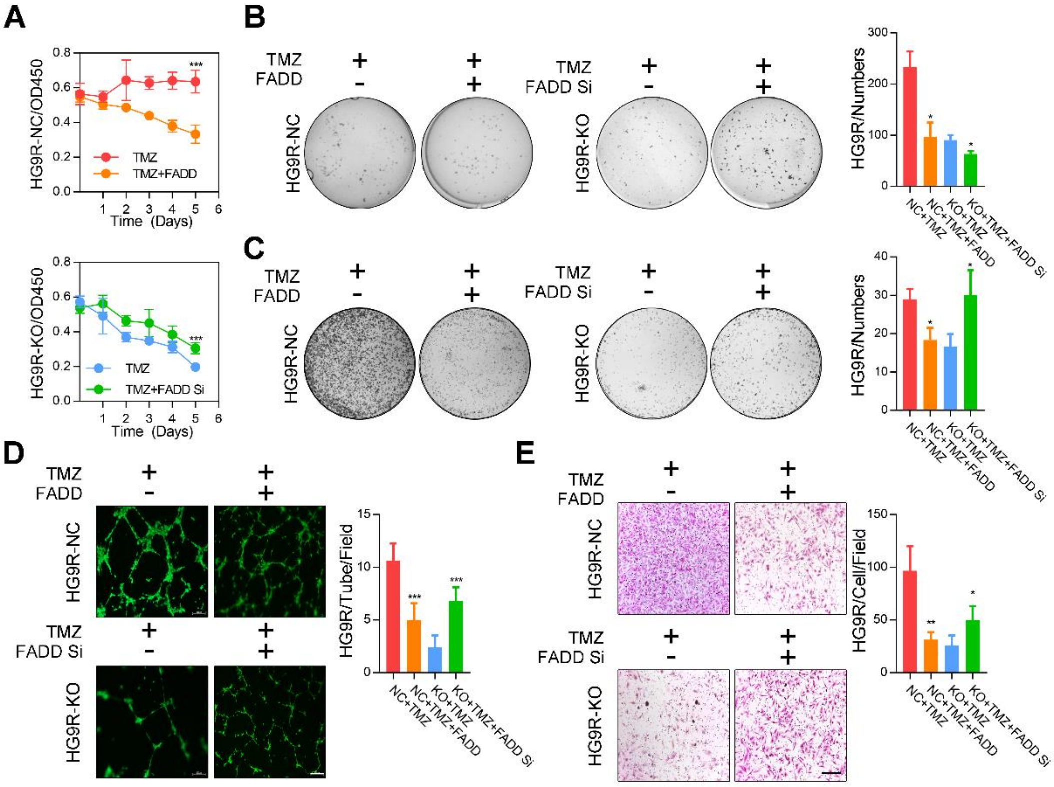

Figure S7. Proliferation, invasive ability and vascular mimicry of *ATRX* NC and *ATRX* KO HG9R cells with *FADD* overexpression or knockdown treated with TMZ.

(A) CCK-8 assays in *ATRX* NC and *ATRX* KO HG9R cells with FADD overexpression or knockdown treated with TMZ. (B-C) Soft agar colony and colony formation assays in *ATRX* NC and *ATRX* KO HG9R cells with *FADD* overexpression or knockdown treated with TMZ. (D) Vascular mimicry assays of *ATRX* NC and *ATRX* KO HG9R cells treated *FADD* overexpression or knockdown treated with TMZ. (E) Transwell assays of *ATRX* NC and *ATRX* KO HG9R cells with FADD overexpression or knockdown treated with TMZ. Scale: 200 μm. The statistics was showed in bar plots. Error bars indicated mean ± SD. * $P < 0.05$, ** $P < 0.01$, *** $P < 0.001$; Student's *t*-test.

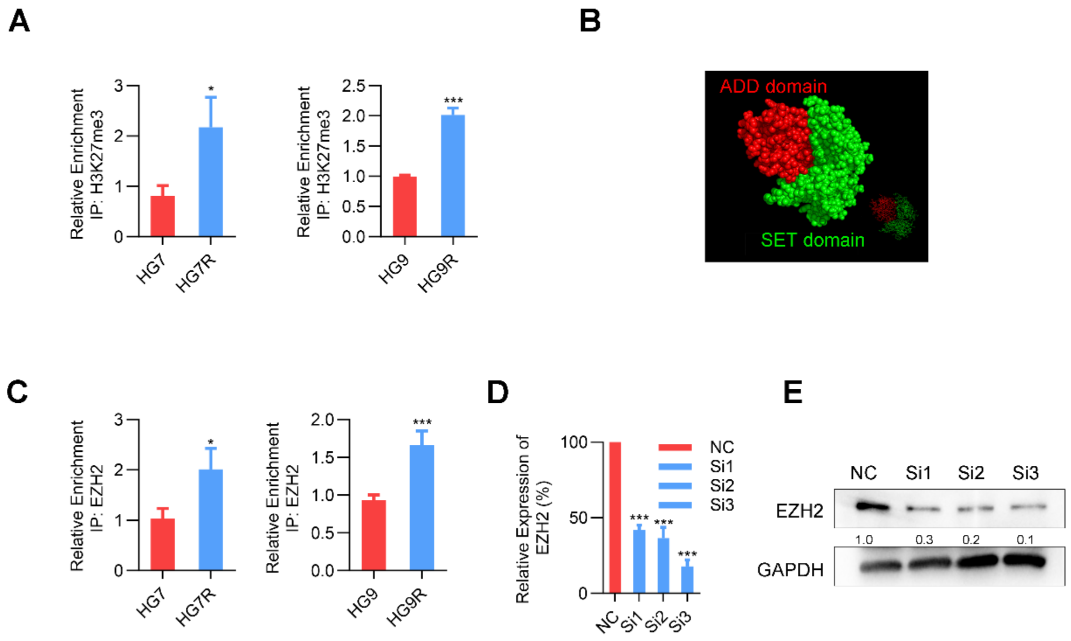

Figure S8. Enrichment of H3K27me3 and EHZ2 in *FADD* promoter region, and prediction of ATRX binding to EZH2.

(A) ChIP-PCR analyses of *FADD* promoter regions with antibodies targeting H3K27me3 in HG7R and HG9R cells and their parental cells. (B) Model of the binding regions of ATRX and EZH2. Cartoon representation of the ATRX- EZH2 crystal structure. ATRX is colored red. EZH2 is colored gray. (C) ChIP-PCR analyses of *FADD* promoter regions with antibodies targeting EZH2 in HG7R and HG9R cells and their parental cells. (D-E) Validation of EZH2 knockdown in HG7R cells by qRT-PCR and western blotting.

Supplementary Table 1. The sequence of the sgRNAs and siRNAs.

**CRISPR**	**Name**	**Sequence （5‘-3’）**	
CRISPR	ATRX-KO-sgRNA-1	TCGTGACGATCCTGAAGACT	
CRISPR	ATRX-KO-sgRNA-2	ACTATGCAGAGCTTGCCAAA	
CRISPR	ATRX-KO-sgRNA-3	ACATACTTTGTTACAATTGA	
**Assay**	**Name**	**Sequence sense（5‘-3’）**	**Sequence anti-sense（5‘-3’）**
Gene Silencing	si-STAT5b-1/sh-STAT5b-1	GCAGAGUUACAGUCACAAAGU	UUUGUGACUGUAACUCUGCAU
Gene Silencing	si-STAT5b-2/sh-STAT5b-2	GCGAGUCUGCUACUGCUAAAG	UUAGCAGUAGCAGACUCGCAG
Gene Silencing	si-STAT5b-3/sh-STAT5b-3	CGAGUCUGCUACUGCUAAAGC	UUUAGCAGUAGCAGACUCGCA
Gene Silencing	si-TET2-1/sh-TET2-1	CGAUUUGAGUGAUAAGAAAGA	UUUCUUAUCACUCAAAUCGGA
Gene Silencing	si-TET2-2/sh-TET2-2	GGUGCUUACUUCAAGCAAAGC	UUUGCUUGAAGUAAGCACCAU
Gene Silencing	si-TET2-3/sh-TET2-3	GGAGCAGAAGUCACAACAAGC	UUGUUGUGACUUCUGCUCCUG
Gene Silencing	si-FADD-1	GCAGUCCUCUUAUUCCUAA	UUAGGAAUAAGAGGACUGC
Gene Silencing	si-FADD-2	GCGAGCUGACCGAGCUCAAGU	UUGAGCUCGGUCAGCUCGCUG
Gene Silencing	si-FADD-3	CGAUGUCAUGGAACUCAGACG	UCUGAGUUCCAUGACAUCGGG
Gene Silencing	si-EZH2-1	GGAUGGUACUUUCAUUGAAGA	UUCAAUGAAAGUACCAUCCUG
Gene Silencing	si-EZH2-2	CCUCAAUGUUUCCAGAUAAGG	UUAUCUGGAAACAUUGAGGAA
Gene Silencing	si-EZH2-3	GCAAAGUACUGUAAGAAUAAU	UAUUCUUACAGUACUUUGCAA

Supplementary Table 2. The details of primary antibodies used for western blot, ChIP, immunoprecipitation, immunohistochemistry and immunofluorescence.

Gene	Manufactory	Catalog number	Application
ATRX	Cell Signaling Technology	#14820	Western blot/ChIP/Immunoprecipitation
ATRX	Boster	A00203-1	Immunohistochemistry/Immunofluorescence
γ-H2AX	Cell Signaling Technology	#9718	Western blot/Immunohistochemistry
STAT5B	Abcam	ab178941	Western blot/Immunoprecipitation
STAT5B	Abcam	ab235934	ChIP
TET2	Abcam	ab18950	Western blot/ChIP/Immunoprecipitation
PARP1	Abcam	ab191217	Western blot
c-PARP1	Abcam	ab32064	Western blot/Immunohistochemistry
FADD	Cell Signaling Technology	#2782	Western blot
FADD	Boster	A00237	Immunohistochemistry
CASP8	Cell Signaling Technology	#4790	Western blot
c-CASP8	Cell Signaling Technology	#9496	Western blot
CASP3	Cell Signaling Technology	#9662	Western blot
c-CASP3	Cell Signaling Technology	#9664	Western blot/Immunohistochemistry
Bcl-XL	Cell Signaling Technology	#2764	Western blot
Bcl2	Cell Signaling Technology	#15071	Western blot
H3K27me3	Cell Signaling Technology	#14820	ChIP
EZH2	Cell Signaling Technology	#5246	Western blot/ChIP/Immunoprecipitation
GAPDH	Zsbio	TA309157	Western blot

Supplementary Table 3. The primers sequences used for qRT-PCR,ChIP-qPCR and pyrosequencing assays.

	**qRT-PCR**			
	Forward 5'-3'	Reverse 5'-3'		
ATRX	ACGGCGTTAGTGGTTTGTCCTC	GCAGCATGTAGCTTCTCTCCTG		
STAT5b	GCCACTGTTCTCTGGGACAATG	ACACGAGGTTCTCCTTGGTCAG		
TET2	GCTTACCGAGACGCTGAGGAAA	AGAGAAGGAGGCACCACAGGTT		
FADD	CTCCTGCGCGAGCTGCTCGC	GCCTTCTCCAATCTTTCCCCAC		
EZH2	GACCTCTGTCTTACTTGTGGAGC	CGTCAGATGGTGCCAGCAATAG		
GAPDH	ACAACTTTGGTATCGTGGAAGG	GCCATCACGCCACAGTTTC		
	**ChIP-qPCR**			
	Forward 5'-3'	Reverse 5'-3'		
**ATRX promoter**	CACCGTCTGAGTCGGGTGTT	AGCGTCACCGTTTAGGGGAG		
**FADD promoter**	TGGAGCGTGTCGCTTTTGTA	TGCCCTCACATCTGTTGGTG		
	**pyrosequencing**			
	Forward 5'-3'	Reverse 5'-3'	Sequence to Analyze	Distance To TSS
**ATRX promoter**	TAGAGGATTTGTTTGTGATTAGTAGAG	TCAATTTAAATCTACTTCCTTCTCATTACA	[illegible]AAGGT ATTTATAGAT T[illegible]	-819

Supplementary Table 4. The differential gene expression in six gene families between LN229 and LN229R.

Gene	Fold Change	P	Gene Families	Gene Title
FOXF1	540.86	3.1E-06	Transcription Factors	forkhead box F1
ALX4	487.08	1.3E-07	Homeodomain Proteins	ALX homeobox 4
FOXL2	425.17	9.3E-09	Oncogenes	forkhead box L2
GATA4	332.35	6.6E-07	Transcription Factors	GATA binding protein 4
ZIC2	218.28	9.7E-06	Transcription Factors	Zic family member 2
ZIC5	206.18	1.5E-06	Transcription Factors	Zic family member 5
PRDM16	151.71	1.7E-06	Oncogenes / Translocated Cancer Genes	PR domain containing 16
EYA2	136.23	2.3E-05	Transcription Factors	EYA transcriptional coactivator and phosphatase 2
EPAS1	135.28	5.7E-08	Transcription Factors	endothelial PAS domain protein 1
PAX2	93.31	2.3E-07	Homeodomain Proteins	paired box 2
WT1	85.58	0.00208	Tumor Suppressors.	Wilms tumor 1
NKX2-1	83.85	1.5E-08	Homeodomain Proteins	NK2 homeobox 1
PITX1	80.15	1.4E-08	Homeodomain Proteins	paired-like homeodomain 1
ZNF239	71.52	0.00045	Transcription Factors	zinc finger protein 239
MSC	55.37	0.00013	Transcription Factors	musculin
HMX1	49.74	2.1E-07	Homeodomain Proteins	H6 family homeobox 1
TBX1	26.05	9.6E-08	Transcription Factors	T-box 1
MSX2	25.01	5.4E-06	Homeodomain Proteins	msh homeobox 2
SMAD6	21.08	1.7E-05	Transcription Factors	SMAD family member 6
TSHZ3	16.80	4.8E-07	Homeodomain Proteins	teashirt zinc finger homeobox 3
FOXP1	15.92	3.3E-06	Oncogenes / Translocated Cancer Genes	forkhead box P1
ZFHX2	15.75	5.3E-05	Homeodomain Proteins	zinc finger homeobox 2
GATA3	13.45	4E-07	Tumor Suppressors.	GATA binding protein 3
RUNX2	13.16	1.2E-05	Transcription Factors	runt-related transcription factor 2
MSX2P1	12.31	4.6E-06	Homeodomain Proteins	---
MSX1	12.25	4.9E-07	Homeodomain Proteins	msh homeobox 1
MNX1	12.11	3.7E-08	Oncogenes / Translocated Cancer Genes / Homeodomain Proteins	motor neuron and pancreas homeobox 1
CITED2	11.13	2.3E-08	Transcription Factors	Cbp/p300-interacting transactivator, with Glu/Asp rich carboxy-terminal domain, 2
AR	10.73	1.6E-08	Transcription Factors	androgen receptor
NR2F1	10.58	6.7E-06	Transcription Factors	nuclear receptor subfamily 2, group F, member 1
IRF1	10.41	3.7E-07	Transcription Factors	interferon regulatory factor 1
PAX3	10.26	2.8E-05	Oncogenes / Translocated Cancer Genes / Homeodomain Proteins	paired box 3
ZNF334	10.02	0.00046	Transcription Factors	zinc finger protein 334
PRDM12	9.91	4.3E-05	Transcription Factors	PR domain containing 12
SOX9	9.15	2.4E-06	Transcription Factors	SRY box 9
MAFB	9.13	0.02074	Oncogenes / Translocated Cancer Genes	v-maf avian musculoaponeurotic fibrosarcoma oncogene homolog B
FHL1	8.97	5.6E-07	Transcription Factors	four and a half LIM domains 1
FOXA1	8.93	1.2E-06	Transcription Factors	forkhead box A1
MID1	8.57	5.6E-06	Transcription Factors	midline 1
HOXB4	7.83	8.8E-05	Homeodomain Proteins	homeobox B4
ZHX1	7.67	1.4E-07	Homeodomain Proteins	zinc fingers and homeoboxes 1
HIC1	7.47	0.00017	Transcription Factors	hypermethylated in cancer 1
RNF141	7.33	3.7E-05	Transcription Factors	ring finger protein 141
CDX1	7.24	0.00206	Homeodomain Proteins	caudal type homeobox 1
IRF7	6.90	2.3E-06	Transcription Factors	interferon regulatory factor 7
ZNF319	6.76	8.1E-07	Transcription Factors	zinc finger protein 319
TCF19	6.53	1.2E-05	Transcription Factors	transcription factor 19
LHX4	6.48	1.2E-05	Homeodomain Proteins	LIM homeobox 4
GLIS2	6.38	2.6E-06	Transcription Factors	GLIS family zinc finger 2
CHD5	6.33	0.00096	Transcription Factors	chromodomain helicase DNA binding protein 5
TBX3	6.26	4.4E-08	Transcription Factors	T-box 3
FLI1	6.21	0.00025	Oncogenes / Translocated Cancer Genes	Fli-1 proto-oncogene, ETS transcription factor
HOMEZ	6.19	0.00023	Homeodomain Proteins	homeobox and leucine zipper encoding
PPARGC1	6.11	7.2E-06	Transcription Factors	peroxisome proliferator-activated receptor gamma, coactivator 1 alpha
NR3C1	5.99	9.5E-07	Transcription Factors	nuclear receptor subfamily 3, group C, member 1 (glucocorticoid receptor)
HOXC13	5.94	1.3E-05	Oncogenes / Translocated Cancer Genes / Homeodomain Proteins	homeobox C13
RASSF7	5.93	1.3E-06	Transcription Factors	Ras association (RalGDS/AF-6) domain family (N-terminal) member 7
CBX3	5.76	5.4E-07	Transcription Factors	chromobox homolog 3
PRDM1	5.74	2.6E-06	Transcription Factors	PR domain containing 1, with ZNF domain
ONECUT2	5.30	3.1E-07	Homeodomain Proteins	one cut homeobox 2
HSF2BP	5.24	1.1E-05	Transcription Factors	heat shock transcription factor 2 binding protein /// uncharacterized LOC105379490
NR4A3	5.03	7.4E-05	Oncogenes / Translocated Cancer Genes / Protein Kinases	nuclear receptor subfamily 4, group A, member 3
PLRG1	4.99	5.5E-07	Transcription Factors	pleiotropic regulator 1
ZSCAN2	4.99	7.8E-05	Transcription Factors	zinc finger and SCAN domain containing 2
SMAD9	4.98	2E-05	Transcription Factors	SMAD family member 9
TRIM22	4.83	2.3E-06	Transcription Factors	tripartite motif containing 22
CREB3L1	4.76	1.9E-05	Transcription Factors	cAMP responsive element binding protein 3-like 1
SUPT3H	4.76	0.00094	Transcription Factors	SPT3 homolog, SAGA and STAGA complex component
TAF1A	4.75	0.00014	Transcription Factors	TATA box binding protein (TBP)-associated factor, RNA polymerase I, A, 48kDa
OPTN	4.70	9.1E-07	Transcription Factors	optineurin
LHX3	4.69	0.00282	Homeodomain Proteins	LIM homeobox 3
ZNF423	4.57	0.00019	Transcription Factors	zinc finger protein 423
HCFC2	4.41	1.2E-05	Transcription Factors	host cell factor C2
HOXA13	4.39	5E-06	Oncogenes / Translocated Cancer Genes / Homeodomain Proteins	homeobox A13
LHX6	4.39	0.00034	Homeodomain Proteins	LIM homeobox 6
CIR1	4.33	2.3E-05	Transcription Factors	corepressor interacting with RBPJ, 1
IRX2	4.19	1E-05	Homeodomain Proteins	iroquois homeobox 2
CEBPD	4.17	2.1E-06	Transcription Factors	CCAAT/enhancer binding protein (C/EBP), delta
ZKSCAN5	4.10	4.3E-07	Transcription Factors	zinc finger with KRAB and SCAN domains 5
NFIL3	4.08	8.3E-07	Transcription Factors	nuclear factor, interleukin 3 regulated
FOXG1	3.96	0.00029	Transcription Factors	forkhead box G1
SNAPC3	3.95	9.5E-06	Transcription Factors	small nuclear RNA activating complex polypeptide 3
SIM2	3.93	0.0011	Transcription Factors	single-minded family bHLH transcription factor 2
CECR6	3.87	0.00011	Transcription Factors	cat eye syndrome chromosome region, candidate 6
NR1H3	3.83	1.4E-06	Transcription Factors	nuclear receptor subfamily 1, group H, member 3
HES4	3.82	7E-06	Transcription Factors	hes family bHLH transcription factor 4
NFE2L2	3.80	5.5E-06	Transcription Factors	nuclear factor, erythroid 2-like 2
EYA4	3.76	7.6E-06	Transcription Factors	EYA transcriptional coactivator and phosphatase 4
ATF3	3.75	1.2E-05	Transcription Factors	activating transcription factor 3
HIVEP2	3.72	9.9E-06	Transcription Factors	human immunodeficiency virus type I enhancer binding protein 2
NCOA1	3.72	0.00396	Oncogenes / Translocated Cancer Genes	nuclear receptor coactivator 1
SCML2	3.68	3.7E-07	Transcription Factors	sex comb on midleg-like 2 (Drosophila)
ZNF132	3.68	6.6E-05	Transcription Factors	zinc finger protein 132
RNF112	3.68	0.00012	Transcription Factors	ring finger protein 112
IRX5	3.62	1.9E-06	Homeodomain Proteins	iroquois homeobox 5
TFAP2A	3.60	4.4E-06	Transcription Factors	transcription factor AP-2 alpha (activating enhancer binding protein 2 alpha)
CREB3	3.58	4.1E-05	Transcription Factors	cAMP responsive element binding protein 3
PPARA	3.53	0.00013	Transcription Factors	peroxisome proliferator-activated receptor alpha
GTF3C3	3.51	5E-05	Transcription Factors	general transcription factor IIIC subunit 3
ZNF823	3.51	7.7E-06	Transcription Factors	zinc finger protein 823
IRF9	3.49	5.8E-05	Transcription Factors	interferon regulatory factor 9
KDM4D	3.48	4E-07	Transcription Factors	lysine (K)-specific demethylase 4D
ONECUT3	3.47	0.00046	Homeodomain Proteins	one cut homeobox 3
ERCC2	3.39	2.7E-05	Tumor Suppressors.	excision repair cross-complementation group 2
NR1D2	3.38	0.00048	Transcription Factors	nuclear receptor subfamily 1, group D, member 2
SNAI2	3.36	0.00046	Transcription Factors	snail family zinc finger 2
GSC	3.36	4.5E-05	Homeodomain Proteins	goosecoid homeobox
MED17	3.35	6.2E-06	Transcription Factors	mediator complex subunit 17
FOSL2	3.35	1.5E-06	Transcription Factors	FOS-like antigen 2
FOXJ2	3.34	8.9E-06	Transcription Factors	forkhead box J2
KLF2	3.33	7E-08	Transcription Factors	Kruppel-like factor 2
CASZ1	3.33	2.1E-05	Transcription Factors	castor zinc finger 1
ILF2	3.31	1.9E-05	Transcription Factors	interleukin enhancer binding factor 2
MYT1L	3.31	0.00404	Transcription Factors	myelin transcription factor 1-like
DPF2	3.31	2.8E-06	Transcription Factors	D4, zinc and double PHD fingers family 2
ZNF250	3.29	9.8E-05	Transcription Factors	zinc finger protein 250
SOX18	3.24	1.5E-06	Transcription Factors	SRY box 18
STAT1	3.23	0.00027	Transcription Factors	signal transducer and activator of transcription 1
FHL2	3.15	8.2E-07	Transcription Factors	four and a half LIM domains 2
MITF	3.15	1.3E-06	Oncogenes	microphthalmia-associated transcription factor
MED21	3.10	0.00012	Transcription Factors	mediator complex subunit 21
TFEC	3.10	0.0441	Transcription Factors	transcription factor EC
ADPGK	3.08	1.3E-05	Transcription Factors	ADP-dependent glucokinase
MYC	3.07	1.7E-05	Oncogenes / Translocated Cancer Genes	v-myc avian myelocytomatosis viral oncogene homolog
TSC22D2	3.05	1.7E-06	Transcription Factors	TSC22 domain family, member 2
IRX1	3.03	6.2E-06	Homeodomain Proteins	iroquois homeobox 1
MTF2	3.02	4.3E-05	Transcription Factors	metal response element binding transcription factor 2
ZSCAN12	3.02	3.7E-05	Transcription Factors	zinc finger and SCAN domain containing 12
MYB	2.98	0.013	Oncogenes / Translocated Cancer Genes	v-myb avian myeloblastosis viral oncogene homolog
RBL1	2.97	2E-06	Transcription Factors	retinoblastoma-like 1
MXD1	2.93	2.6E-07	Transcription Factors	MAX dimerization protein 1
CEBPG	2.93	1.8E-05	Transcription Factors	CCAAT/enhancer binding protein (C/EBP), gamma
HLTF	2.93	1.9E-06	Transcription Factors	helicase-like transcription factor
FOXD4	2.91	9.6E-05	Transcription Factors	forkhead box D4
NKX3-1	2.89	4.5E-05	Homeodomain Proteins	NK3 homeobox 1
ZNF85	2.86	0.00074	Transcription Factors	zinc finger protein 85
SOX30	2.84	0.00078	Transcription Factors	SRY box 30
SRA1	2.83	1.3E-06	Transcription Factors	steroid receptor RNA activator 1
TRIM26	2.77	1.4E-05	Transcription Factors	tripartite motif containing 26
SP3	2.77	9.8E-07	Transcription Factors	Sp3 transcription factor
ARID5B	2.76	3.4E-05	Transcription Factors	AT rich interactive domain 5B (MRF1-like)
TEAD4	2.76	4.6E-05	Transcription Factors	TEA domain family member 4
ZNF205	2.75	2.1E-05	Transcription Factors	zinc finger protein 205
HOXC10	2.74	0.00011	Homeodomain Proteins	homeobox C10
TBX15	2.74	8.7E-06	Transcription Factors	T-box 15
ZNF691	2.72	7.2E-06	Transcription Factors	zinc finger protein 691
TGIF2	2.72	4.8E-06	Homeodomain Proteins	TGFB-induced factor homeobox 2
DEAF1	2.70	1.1E-07	Transcription Factors	DEAF1 transcription factor
POU5F1	2.70	0.01727	Oncogenes / Translocated Cancer Genes / Homeodomain Proteins	POU class 5 homeobox 1 /// POU class 5 homeobox 1B /// POU class 5 homeobox 1 pseudogene 3 /// POU class 5 homeobox 1 pseudogene 4
MED23	2.69	0.00156	Transcription Factors	mediator complex subunit 23
EYA1	2.69	0.00184	Transcription Factors	EYA transcriptional coactivator and phosphatase 1
ZNF107	2.66	0.00022	Transcription Factors	zinc finger protein 107
PML	2.66	0.00013	Oncogenes / Translocated Cancer Genes	promyelocytic leukemia
RUNX3	2.66	0.00141	Transcription Factors	runt-related transcription factor 3
DDIT3	2.63	5.6E-05	Oncogenes / Translocated Cancer Genes	DNA-damage-inducible transcript 3
ZBTB43	2.63	1.5E-05	Transcription Factors	zinc finger and BTB domain containing 43
ZNF175	2.57	3.3E-05	Transcription Factors	zinc finger protein 175
HDAC2	2.57	3.4E-05	Transcription Factors	histone deacetylase 2
SIX1	2.56	0.0001	Homeodomain Proteins	SIX homeobox 1
ZNF273	2.53	0.00039	Transcription Factors	zinc finger protein 273
ZNF174	2.52	4E-05	Transcription Factors	zinc finger protein 174
ZXDB	2.52	5.4E-06	Transcription Factors	zinc finger, X-linked, duplicated B
ZNF629	2.51	2E-05	Transcription Factors	zinc finger protein 629
ZFP28	2.51	0.00031	Transcription Factors	ZFP28 zinc finger protein
MECOM	2.50	0.00015	Oncogenes / Translocated Cancer Genes	MDS1 and EVI1 complex locus
SOX12	2.50	1.2E-05	Transcription Factors	SRY box 12
MYBL1	2.49	8.9E-07	Transcription Factors	v-myb avian myeloblastosis viral oncogene homolog-like 1
ZFPM2	2.49	0.00015	Transcription Factors	zinc finger protein, FOG family member 2
RNF41	2.48	2.3E-05	Transcription Factors	ring finger protein 41, E3 ubiquitin protein ligase
ZNF14	2.47	1.2E-06	Transcription Factors	zinc finger protein 14
NUFIP2	2.46	1.3E-05	Transcription Factors	nuclear fragile X mental retardation protein interacting protein 2
CREBZF	2.45	1.8E-05	Transcription Factors	CREB/ATF bZIP transcription factor
RAX	2.45	0.0148	Homeodomain Proteins	retina and anterior neural fold homeobox
SUB1	2.44	8E-05	Transcription Factors	SUB1 homolog, transcriptional regulator
RFX2	2.44	0.00022	Transcription Factors	regulatory factor X, 2 (influences HLA class II expression)
CDR2	2.43	2.8E-05	Transcription Factors	cerebellar degeneration related protein 2 /// cerebellar degeneration-related protein 2
ESRRA	2.43	3.5E-05	Transcription Factors	estrogen-related receptor alpha
NR5A1	2.42	0.02993	Transcription Factors	nuclear receptor subfamily 5, group A, member 1
ZFP36	2.42	7.7E-06	Transcription Factors	ZFP36 ring finger protein
ZNF160	2.41	4.7E-05	Transcription Factors	zinc finger protein 160
REPIN1	2.41	3.7E-06	Transcription Factors	replication initiator 1
MAPK8IP1	2.39	8.6E-05	Transcription Factors	mitogen-activated protein kinase 8 interacting protein 1
ATF2	2.38	0.00015	Transcription Factors	activating transcription factor 2
LARP1	2.37	0.00053	Transcription Factors	La ribonucleoprotein domain family, member 1
ZKSCAN3	2.35	2.8E-07	Transcription Factors	zinc finger with KRAB and SCAN domains 3
DPF1	2.34	2.2E-07	Transcription Factors	D4, zinc and double PHD fingers family 1
IRX3	2.33	2E-06	Homeodomain Proteins	iroquois homeobox 3
IRX6	2.33	0.0465	Homeodomain Proteins	---
ZNF195	2.32	0.00017	Transcription Factors	zinc finger protein 195
TFDP2	2.31	0.00012	Transcription Factors	transcription factor Dp-2 (E2F dimerization partner 2)
ZNF267	2.31	0.00085	Transcription Factors	zinc finger protein 267
BRD7	2.28	0.00029	Transcription Factors	bromodomain containing 7
ZNF19	2.26	0.00044	Transcription Factors	zinc finger protein 19
ZNF23	2.25	1.2E-05	Transcription Factors	zinc finger protein 23
GTF2E1	2.25	0.00011	Transcription Factors	general transcription factor IIE subunit 1
MED7	2.25	2.7E-06	Transcription Factors	mediator complex subunit 7
PRICKLE3	2.23	0.00039	Transcription Factors	prickle homolog 3
NR2C1	2.22	5.9E-07	Transcription Factors	nuclear receptor subfamily 2, group C, member 1
BRPF3	2.22	2E-05	Transcription Factors	bromodomain and PHD finger containing 3
ZNF282	2.22	0.00053	Transcription Factors	zinc finger protein 282
AHR	2.21	7.9E-05	Transcription Factors	aryl hydrocarbon receptor
TRIM25	2.20	4.6E-05	Transcription Factors	tripartite motif containing 25
ZNF576	2.20	0.00958	Transcription Factors	zinc finger protein 576
SEBOX	2.18	0.00344	Homeodomain Proteins	SEBOX homeobox
ZNF304	2.17	0.0001	Transcription Factors	zinc finger protein 304
RNF4	2.17	0.00016	Transcription Factors	ring finger protein 4
ETS1	2.15	9.4E-05	Transcription Factors	v-ets avian erythroblastosis virus E26 oncogene homolog 1
UBTF	2.14	8.6E-06	Transcription Factors	upstream binding transcription factor, RNA polymerase I
CORO1A	2.13	2.3E-05	Transcription Factors	coronin, actin binding protein, 1A
RNF24	2.13	8.1E-06	Transcription Factors	ring finger protein 24
ATRX	2.13	0.00093	Transcription Factors	alpha thalassemia/mental retardation syndrome X-linked
TCF7	2.13	8.2E-06	Transcription Factors	transcription factor 7 (T-cell specific, HMG-box)
GTF2B	2.12	0.00183	Transcription Factors	general transcription factor IIB
MED24	2.12	0.00043	Transcription Factors	mediator complex subunit 24
SPIB	2.12	0.0418	Transcription Factors	Spi-B transcription factor (Spi-1/PU.1 related)
ZBTB11	2.11	1.2E-06	Transcription Factors	zinc finger and BTB domain containing 11
ZNF217	2.10	4.3E-05	Transcription Factors	zinc finger protein 217
ZNF92	2.10	3E-05	Transcription Factors	zinc finger protein 92
TRIP6	2.09	0.00011	Transcription Factors	thyroid hormone receptor interactor 6
ZNF20	2.08	0.00231	Transcription Factors	zinc finger protein 20 /// ZNF625-ZNF20 readthrough (NMD candidate)
ZNF30	2.07	0.01104	Transcription Factors	zinc finger protein 30
RRN3	2.06	0.00071	Transcription Factors	RRN3 homolog, RNA polymerase I transcription factor /// RRN3 homolog, RNA polymerase I transcription factor pseudogene 1 /// RRN3 homolog, RNA polymerase I transcription factor pseudogene 2
MBD2	2.06	0.00011	Transcription Factors	methyl-CpG binding domain protein 2
ZNF552	2.04	6.5E-05	Transcription Factors	zinc finger protein 552
TBPL1	2.04	7.1E-07	Transcription Factors	TBP-like 1
TP53	2.03	0.00042	Tumor Suppressors.	tumor protein p53
LAS1L	2.02	0.00064	Transcription Factors	LAS1-like, ribosome biogenesis factor
IRF3	2.02	9.9E-05	Transcription Factors	interferon regulatory factor 3
MBD4	2.01	0.00018	Transcription Factors	methyl-CpG binding domain 4 DNA glycosylase
STAT5A	2.00	0.00948	Transcription Factors	signal transducer and activator of transcription 5A
ATF7IP	2.00	0.00023	Transcription Factors	activating transcription factor 7 interacting protein
STAT3	1.98	8.3E-07	Transcription Factors	signal transducer and activator of transcription 3 (acute-phase response factor)
ETV2	1.98	0.00029	Transcription Factors	ets variant 2
ZBTB24	1.98	4.6E-05	Transcription Factors	zinc finger and BTB domain containing 24
CNOT4	1.97	0.00414	Transcription Factors	CCR4-NOT transcription complex subunit 4
SHOX2	1.96	4E-05	Homeodomain Proteins	short stature homeobox 2
ATF6	1.96	8E-05	Transcription Factors	activating transcription factor 6
MXD4	1.96	0.00019	Transcription Factors	MAX dimerization protein 4
ZNF746	1.96	6.5E-06	Transcription Factors	zinc finger protein 746
TULP3	1.95	2.6E-05	Transcription Factors	tubby like protein 3
SAMD4B	1.94	0.00011	Transcription Factors	sterile alpha motif domain containing 4B
NMI	1.94	3.8E-06	Transcription Factors	N-myc (and STAT) interactor

MTA2	1.94	0.00026	Transcription Factors	metastasis associated 1 family member 2
NEUROD(	1.92	0.00862	Transcription Factors	neuronal differentiation 6
ZNF639	1.92	0.00059	Transcription Factors	zinc finger protein 639
ZNF696	1.91	5.9E-05	Transcription Factors	zinc finger protein 696
E2F1	1.90	8.1E-05	Transcription Factors	E2F transcription factor 1
KLF10	1.90	7.4E-05	Transcription Factors	Kruppel-like factor 10
TRIM62	1.90	0.00015	Transcription Factors	tripartite motif containing 62
ELK4	1.89	0.00032	Oncogenes / Translocated Cancer Genes	ELK4, ETS-domain protein (SRF accessory protein 1)
ZNF254	1.89	0.00095	Transcription Factors	zinc finger protein 254
SRF	1.89	0.00043	Transcription Factors	serum response factor
E2F4	1.89	0.00121	Transcription Factors	E2F transcription factor 4, p107/p130-binding
NR1D1	1.88	2.6E-05	Transcription Factors	nuclear receptor subfamily 1, group D, member 1 /// thyroid hormone receptor, alpha
TSC22D3	1.87	1.2E-05	Transcription Factors	TSC22 domain family, member 3
HIVEP1	1.87	3.3E-05	Transcription Factors	human immunodeficiency virus type I enhancer binding protein 1
GBX2	1.87	0.00013	Homeodomain Proteins	gastrulation brain homeobox 2
ZFHX3	1.87	5.2E-05	Homeodomain Proteins	zinc finger homeobox 3
MSRB2	1.87	3.9E-06	Transcription Factors	methionine sulfoxide reductase B2
STAT5B	1.86	0.0002	Transcription Factors	signal transducer and activator of transcription 5B
GRHL1	1.86	5.3E-05	Transcription Factors	grainyhead-like transcription factor 1
ZNF91	1.85	0.00335	Transcription Factors	zinc finger protein 91
FOXF2	1.85	1.7E-05	Transcription Factors	forkhead box F2
FOSL1	1.85	2.2E-05	Transcription Factors	FOS-like antigen 1
ZNF275	1.84	1.3E-05	Transcription Factors	zinc finger protein 275
PKNOX1	1.84	0.00078	Homeodomain Proteins	PBX/knotted 1 homeobox 1
PLAGL2	1.84	3E-05	Transcription Factors	pleiomorphic adenoma gene-like 2
TCEAL1	1.83	5.7E-05	Transcription Factors	transcription elongation factor A (SII)-like 1
BRD3	1.82	8.5E-05	Oncogenes / Translocated Cancer Genes / Protein Kinases	bromodomain containing 3
LMX1B	1.81	0.00498	Homeodomain Proteins	LIM homeobox transcription factor 1, beta
CNOT8	1.80	1.7E-06	Transcription Factors	CCR4-NOT transcription complex subunit 8
KLF4	1.80	0.00014	Transcription Factors	Kruppel-like factor 4 (gut)
ATF5	1.79	0.00022	Transcription Factors	activating transcription factor 5
RXRA	1.77	6.5E-05	Transcription Factors	retinoid X receptor alpha
TMF1	1.77	0.00111	Transcription Factors	TATA element modulatory factor 1
NCOA2	1.77	0.00513	Oncogenes / Translocated Cancer Genes	nuclear receptor coactivator 2
NKX1-1	1.77	0.00032	Transcription Factors	NK1 homeobox 1
FOXH1	1.76	2.2E-05	Transcription Factors	forkhead box H1
ZNF251	1.76	4.5E-05	Transcription Factors	zinc finger protein 251
RNF103	1.74	0.00015	Transcription Factors	ring finger protein 103
ZNF264	1.74	3.4E-05	Transcription Factors	zinc finger protein 264
SMARCC2	1.74	0.00178	Transcription Factors	SWI/SNF related, matrix associated, actin dependent regulator of chromatin, subfamily c, member 2
ZNF430	1.74	0.00013	Transcription Factors	zinc finger protein 430
PBX2	1.73	0.00037	Homeodomain Proteins	pre-B-cell leukemia homeobox 2
KLF13	1.73	5.4E-06	Transcription Factors	Kruppel-like factor 13 /// proline-rich protein HaeIII subfamily 1-like
ZNF410	1.73	0.00031	Transcription Factors	zinc finger protein 410
TNRC6A	1.72	0.00035	Transcription Factors	trinucleotide repeat containing 6A
CREG1	1.72	8.8E-05	Transcription Factors	cellular repressor of E1A-stimulated genes 1
TGFB1I1	1.71	8E-05	Transcription Factors	transforming growth factor beta 1 induced transcript 1
SERTAD1	1.71	3.5E-05	Transcription Factors	SERTA domain containing 1
ZSCAN20	1.71	0.00054	Transcription Factors	zinc finger and SCAN domain containing 20
KAT2A	1.71	5.8E-05	Transcription Factors	K(lysine) acetyltransferase 2A
MED14	1.70	6.4E-05	Transcription Factors	mediator complex subunit 14
GTF2H1	1.70	1.9E-06	Transcription Factors	general transcription factor IIH subunit 1
MIS18BP1	1.70	0.00018	Transcription Factors	MIS18 binding protein 1
MEIS1	1.70	0.00025	Homeodomain Proteins	Meis homeobox 1
NFYC	1.69	5.9E-06	Transcription Factors	nuclear transcription factor Y subunit gamma
SIX4	1.69	0.00806	Homeodomain Proteins	SIX homeobox 4
GABPB1	1.69	4.5E-05	Transcription Factors	---
NFATC3	1.68	0.0001	Transcription Factors	nuclear factor of activated T-cells, cytoplasmic, calcineurin-dependent 3
ATF7	1.68	0.0002	Transcription Factors	activating transcription factor 7
NRF1	1.68	0.00593	Transcription Factors	nuclear respiratory factor 1
SIN3B	1.68	7.1E-05	Transcription Factors	SIN3 transcription regulator family member B
ELF3	1.68	0.03219	Transcription Factors	E74-like factor 3 (ets domain transcription factor, epithelial-specific)
ZNF148	1.67	0.00011	Transcription Factors	zinc finger protein 148
ARNT2	1.66	6.4E-05	Transcription Factors	aryl-hydrocarbon receptor nuclear translocator 2
THRA	1.66	0.00146	Transcription Factors	thyroid hormone receptor, alpha
GTPBP1	1.66	2.8E-05	Transcription Factors	GTP binding protein 1
ZNF142	1.66	0.00016	Transcription Factors	zinc finger protein 142
NCOR1	1.65	2.7E-05	Transcription Factors	nuclear receptor corepressor 1
ZMYM3	1.65	0.0057	Transcription Factors	zinc finger, MYM-type 3
TAF4	1.64	0.00067	Transcription Factors	TAF4 RNA polymerase II, TATA box binding protein (TBP)-associated factor, 135kDa
PRDM6	1.64	0.00037	Transcription Factors	PR domain containing 6
RNF10	1.63	0.00237	Transcription Factors	ring finger protein 10
ELF4	1.63	5.8E-06	Oncogenes / Translocated Cancer Genes	E74-like factor 4 (ets domain transcription factor)
TBC1D10B	1.63	0.00011	Transcription Factors	TBC1 domain family, member 10B
TUB	1.63	0.0002	Transcription Factors	tubby bipartite transcription factor
ERGIC2	1.62	4.6E-06	Transcription Factors	ERGIC and golgi 2
ZHX3	1.62	5.2E-05	Homeodomain Proteins	zinc fingers and homeoboxes 3
LRRC14	1.62	0.0033	Transcription Factors	leucine rich repeat containing 14
TRIM15	1.61	0.00256	Transcription Factors	tripartite motif containing 15
TFE3	1.61	0.00045	Oncogenes / Translocated Cancer Genes	transcription factor binding to IGHM enhancer 3
SLC25A4(	1.61	0.00046	Transcription Factors	solute carrier family 25, member 40
ID3	1.61	3.1E-05	Transcription Factors	inhibitor of DNA binding 3, dominant negative helix-loop-helix protein
TEF	1.60	0.00075	Transcription Factors	thyrotrophic embryonic factor
PHF11	1.60	0.00047	Transcription Factors	PHD finger protein 11
MED27	1.60	0.00029	Transcription Factors	mediator complex subunit 27
C14orf16(	1.60	0.00226	Transcription Factors	chromosome 14 open reading frame 166
CDK7	1.60	0.00272	Protein Kinases	cyclin-dependent kinase 7
ZMYM6	1.60	0.00137	Transcription Factors	zinc finger, MYM-type 6
SIAH2	1.59	0.00012	Transcription Factors	siah E3 ubiquitin protein ligase 2
BLZF1	1.59	0.0003	Transcription Factors	basic leucine zipper nuclear factor 1
ZNF708	1.59	0.00102	Transcription Factors	zinc finger protein 708
JUN	1.59	0.00042	Oncogenes	jun proto-oncogene
MAFG	1.59	0.00057	Transcription Factors	v-maf avian musculoaponeurotic fibrosarcoma oncogene homolog G
CNBP	1.58	0.00068	Oncogenes / Translocated Cancer Genes	CCHC-type zinc finger, nucleic acid binding protein
ZNF138	1.58	0.04787	Transcription Factors	zinc finger protein 138
ZXDC	1.58	0.0009	Transcription Factors	ZXD family zinc finger C
SNAPC1	1.58	0.00102	Transcription Factors	small nuclear RNA activating complex polypeptide 1
SMARCE1	1.58	9.4E-05	Transcription Factors	SWI/SNF related, matrix associated, actin dependent regulator of chromatin, subfamily e, member 1
HSF1	1.58	0.00134	Transcription Factors	heat shock transcription factor 1
MED1	1.58	0.01356	Transcription Factors	mediator complex subunit 1
DTX2	1.57	5.2E-06	Transcription Factors	deltex 2, E3 ubiquitin ligase
SMAD3	1.57	0.00035	Transcription Factors	SMAD family member 3
GTF2A1	1.56	0.00018	Transcription Factors	general transcription factor IIA 1
ZNF544	1.56	0.00051	Transcription Factors	zinc finger protein 544
NKX6-1	1.56	0.00033	Homeodomain Proteins	NK6 homeobox 1
CCT4	1.55	0.00028	Transcription Factors	chaperonin containing TCP1, subunit 4 (delta)
PDLIM5	1.55	5.6E-05	Transcription Factors	PDZ and LIM domain 5
TAF6	1.55	0.00062	Transcription Factors	TAF6 RNA polymerase II, TATA box binding protein (TBP)-associated factor, 80kDa
KEAP1	1.55	0.00016	Transcription Factors	kelch-like ECH-associated protein 1
ELF1	1.55	0.00015	Transcription Factors	E74-like factor 1 (ets domain transcription factor)
PROX2	1.54	0.02632	Homeodomain Proteins	prospero homeobox 2
PBX4	1.54	6.3E-05	Homeodomain Proteins	pre-B-cell leukemia homeobox 4
WBP5	1.53	0.00052	Transcription Factors	WW domain binding protein 5
SOX15	1.53	0.01953	Transcription Factors	SRY box 15
MED6	1.52	0.00191	Transcription Factors	mediator complex subunit 6
LMX1A	1.52	0.00137	Homeodomain Proteins	LIM homeobox transcription factor 1, alpha
BAZ1B	1.52	0.00036	Transcription Factors	bromodomain adjacent to zinc finger domain 1B
ZNF613	1.51	0.00018	Transcription Factors	zinc finger protein 613
ZNF318	1.51	0.00018	Transcription Factors	zinc finger protein 318
GTF2H3	1.51	0.00017	Transcription Factors	general transcription factor IIH subunit 3
PAX5	1.51	0.00295	Oncogenes / Translocated Cancer Genes / Homeodomain Proteins	paired box 5
CRX	1.51	0.01437	Homeodomain Proteins	cone-rod homeobox
TRIM33	1.51	0.00014	Oncogenes / Translocated Cancer Genes / Protein Kinases	tripartite motif containing 33
MEF2D	1.51	0.01064	Transcription Factors	myocyte enhancer factor 2D
RBBP5	1.50	0.0001	Transcription Factors	retinoblastoma binding protein 5
ZBTB33	1.50	0.00414	Transcription Factors	zinc finger and BTB domain containing 33
ZNF248	1.49	0.00763	Transcription Factors	zinc finger protein 248
OVOL3	1.49	0.00106	Transcription Factors	ovo-like zinc finger 3
ZBTB7B	1.49	0.01738	Transcription Factors	zinc finger and BTB domain containing 7B
EGLN2	1.49	1.6E-05	Transcription Factors	egl-9 family hypoxia-inducible factor 2 /// RAB4B-EGLN2 readthrough (NMD candidate)
TSC22D4	1.48	7E-05	Transcription Factors	TSC22 domain family, member 4
MLLT4	1.48	0.0001	Oncogenes / Translocated Cancer Genes	myeloid/lymphoid or mixed-lineage leukemia; translocated to, 4
ARIH2	1.48	3.6E-05	Transcription Factors	ariadne RBR E3 ubiquitin protein ligase 2
TRIM28	1.47	1.2E-05	Protein Kinases	tripartite motif containing 28
AKAP17A	1.47	0.00084	Transcription Factors	A kinase (PRKA) anchor protein 17A
ZNF414	1.47	0.00415	Transcription Factors	zinc finger protein 414
ZBTB17	1.47	0.00921	Transcription Factors	zinc finger and BTB domain containing 17
ATG4B	1.47	0.00011	Transcription Factors	autophagy related 4B, cysteine peptidase
TAF5	1.47	0.00042	Transcription Factors	TAF5 RNA polymerase II, TATA box binding protein (TBP)-associated factor, 100kDa
TONSL	1.47	0.00086	Transcription Factors	tonsoku-like, DNA repair protein
TBP	1.46	0.00033	Transcription Factors	TATA box binding protein
CIAO1	1.46	0.00454	Transcription Factors	cytosolic iron-sulfur assembly component 1
IRF2	1.46	0.0002	Transcription Factors	interferon regulatory factor 2
ZNF286A	1.46	0.00163	Transcription Factors	zinc finger protein 286A
ZNF362	1.45	0.00029	Transcription Factors	zinc finger protein 362
PPARD	1.45	0.03362	Transcription Factors	peroxisome proliferator-activated receptor delta
BAZ2B	1.45	0.00927	Transcription Factors	bromodomain adjacent to zinc finger domain 2B
ZFP64	1.45	0.0004	Transcription Factors	ZFP64 zinc finger protein
SUPT5H	1.45	4.2E-05	Transcription Factors	SPT5 homolog, DSIF elongation factor subunit
TAF1	1.44	0.0003	Protein Kinases	TAF1 RNA polymerase II, TATA box binding protein (TBP)-associated factor, 250kDa /// TAF1 RNA polymerase II, TATA box binding protein (TBP)-associated factor, 210kDa-like
PREB	1.44	0.04542	Transcription Factors	prolactin regulatory element binding
DPF3	1.44	0.03157	Transcription Factors	D4, zinc and double PHD fingers, family 3
MECP2	1.44	0.00088	Transcription Factors	methyl-CpG binding protein 2
NFKBIL1	1.44	0.00221	Transcription Factors	nuclear factor of kappa light polypeptide gene enhancer in B-cells inhibitor-like 1
ZNF182	1.43	0.01692	Transcription Factors	zinc finger protein 182
RARB	1.43	0.00017	Transcription Factors	retinoic acid receptor, beta
PRDM11	1.43	0.00286	Transcription Factors	PR domain containing 11
CHD2	1.43	0.00016	Transcription Factors	chromodomain helicase DNA binding protein 2
CREBBP	1.43	0.0001	Oncogenes / Translocated Cancer Genes	CREB binding protein
FOXM1	1.43	0.00206	Transcription Factors	forkhead box M1
TAF2	1.41	0.00075	Transcription Factors	TAF2 RNA polymerase II, TATA box binding protein (TBP)-associated factor, 150kDa
EZH2	1.40	2.3E-06	Transcription Factors	enhancer of zeste 2 polycomb repressive complex 2 subunit
FOXK2	1.40	0.00078	Transcription Factors	forkhead box K2
RNF2	1.40	0.00993	Transcription Factors	ring finger protein 2
CNOT3	1.40	0.00025	Transcription Factors	CCR4-NOT transcription complex subunit 3
ZNF646	1.40	0.00255	Transcription Factors	zinc finger protein 646
MAFK	1.40	0.00058	Transcription Factors	v-maf avian musculoaponeurotic fibrosarcoma oncogene homolog K
ARID3A	1.40	0.00017	Transcription Factors	AT rich interactive domain 3A (BRIGHT-like)
TRIP13	1.39	0.00299	Transcription Factors	thyroid hormone receptor interactor 13
NCOA4	1.39	0.0016	Oncogenes / Translocated Cancer Genes	nuclear receptor coactivator 4
AFF4	1.39	0.00191	Oncogenes / Translocated Cancer Genes	AF4/FMR2 family, member 4
BMI1	1.39	0.00089	Transcription Factors	BMI1 proto-oncogene, polycomb ring finger
DRAP1	1.39	0.00036	Transcription Factors	DR1-associated protein 1 (negative cofactor 2 alpha)
SIAH1	1.39	3.6E-05	Transcription Factors	siah E3 ubiquitin protein ligase 1
CLOCK	1.39	0.04799	Transcription Factors	clock circadian regulator
SMAD7	1.39	0.00253	Transcription Factors	SMAD family member 7
HES1	1.39	0.00024	Transcription Factors	hes family bHLH transcription factor 1
SMYD5	1.38	0.01548	Transcription Factors	SMYD family member 5
KDM3B	1.38	0.00023	Transcription Factors	lysine (K)-specific demethylase 3B
CHD6	1.38	0.00016	Transcription Factors	chromodomain helicase DNA binding protein 6
ZFR	1.38	0.00013	Transcription Factors	zinc finger RNA binding protein
XBP1	1.38	0.01423	Transcription Factors	X-box binding protein 1
ZFHX4	1.37	0.00091	Homeodomain Proteins	zinc finger homeobox 4
ZNF131	1.37	0.00034	Transcription Factors	zinc finger protein 131
ZNF79	1.37	0.00047	Transcription Factors	zinc finger protein 79
GFI1	1.37	0.00712	Transcription Factors	growth factor independent 1 transcription repressor
LDOC1	1.37	0.00019	Transcription Factors	leucine zipper, down-regulated in cancer 1
RUNX1T1	1.36	0.00492	Oncogenes / Translocated Cancer Genes	runt-related transcription factor 1; translocated to, 1 (cyclin D-related)
NR1I3	1.36	0.00153	Transcription Factors	nuclear receptor subfamily 1, group I, member 3
SND1	1.36	8.8E-06	Transcription Factors	staphylococcal nuclease and tudor domain containing 1
ZNF135	1.36	0.00261	Transcription Factors	zinc finger protein 135
RNF113A	1.36	0.00031	Transcription Factors	ring finger protein 113A
FOXE3	1.36	0.00869	Transcription Factors	forkhead box E3
NFRKB	1.36	0.00082	Transcription Factors	nuclear factor related to kappaB binding protein
CREB1	1.36	0.00433	Oncogenes / Translocated Cancer Genes	cAMP responsive element binding protein 1
FOXN3	1.35	0.04893	Transcription Factors	forkhead box N3
MBD1	1.35	0.00078	Transcription Factors	methyl-CpG binding domain protein 1
NFKBIB	1.35	0.00246	Transcription Factors	nuclear factor of kappa light polypeptide gene enhancer in B-cells inhibitor, beta
TEAD3	1.35	0.00166	Transcription Factors	TEA domain family member 3
ESR1	1.35	0.00259	Transcription Factors	estrogen receptor 1
ZNF17	1.35	0.00756	Transcription Factors	zinc finger protein 17
ZNF3	1.35	0.00112	Transcription Factors	zinc finger protein 3
LHX1	1.34	0.0464	Homeodomain Proteins	LIM homeobox 1
ZFAND3	1.34	0.00051	Transcription Factors	zinc finger, AN1-type domain 3
RNF114	1.34	0.00106	Transcription Factors	ring finger protein 114
ZNF197	1.34	0.01714	Transcription Factors	zinc finger protein 197
ZBTB25	1.34	0.00794	Transcription Factors	zinc finger and BTB domain containing 25
CREBL2	1.33	0.0002	Transcription Factors	cAMP responsive element binding protein-like 2
PTTG1IP	1.33	0.00258	Transcription Factors	pituitary tumor-transforming 1 interacting protein
RBL2	1.33	0.00117	Transcription Factors	retinoblastoma-like 2
ZNF473	1.33	0.00029	Transcription Factors	zinc finger protein 473
ZMYND1:	1.32	6E-05	Transcription Factors	zinc finger, MYND-type containing 11
TCF3	1.32	0.0082	Oncogenes / Translocated Cancer Genes	transcription factor 3
ZNF274	1.32	0.00062	Transcription Factors	zinc finger protein 274
CBX7	1.32	0.00012	Transcription Factors	chromobox homolog 7
NFX1	1.32	0.00099	Transcription Factors	nuclear transcription factor, X-box binding 1
TRIM24	1.32	0.01491	Oncogenes / Translocated Cancer Genes / Protein Kinases	tripartite motif containing 24
SMAD5	1.32	0.00476	Transcription Factors	SMAD family member 5
TECPR1	1.32	0.00659	Transcription Factors	tectonin beta-propeller repeat containing 1
RLIM	1.31	0.00078	Transcription Factors	ring finger protein, LIM domain interacting
JMJD4	1.31	2.9E-05	Transcription Factors	jumonji domain containing 4
TRIP4	1.31	0.00141	Transcription Factors	thyroid hormone receptor interactor 4
ZBTB20	1.31	0.00216	Transcription Factors	zinc finger and BTB domain containing 20
ETV5	1.30	0.02811	Oncogenes / Translocated Cancer Genes	ets variant 5
SNAPC2	1.30	0.00148	Transcription Factors	small nuclear RNA activating complex polypeptide 2
SAP30BP	1.30	0.00721	Transcription Factors	SAP30 binding protein
ZNF37A	1.30	0.0021	Transcription Factors	zinc finger protein 37A
ARNTL	1.30	0.00181	Transcription Factors	aryl hydrocarbon receptor nuclear translocator-like
RCOR1	1.29	0.00019	Transcription Factors	REST corepressor 1
NR2F6	1.29	0.01281	Transcription Factors	nuclear receptor subfamily 2, group F, member 6
PRDM4	1.29	2.2E-06	Transcription Factors	PR domain containing 4
E4F1	1.29	0.00178	Transcription Factors	E4F transcription factor 1
ARID1B	1.29	0.01847	Transcription Factors	AT rich interactive domain 1B (SWI1-like)
GTF2E2	1.29	0.00128	Transcription Factors	general transcription factor IIE subunit 2
TCERG1	1.28	0.0052	Transcription Factors	transcription elongation regulator 1
TRMT1	1.28	0.00105	Transcription Factors	tRNA methyltransferase 1
PSIP1	1.28	0.00893	Oncogenes / Translocated Cancer Genes	PC4 and SFRS1 interacting protein 1
TAL1	1.28	0.00368	Oncogenes / Translocated Cancer Genes	T-cell acute lymphocytic leukemia 1
TRIP10	1.28	0.01654	Transcription Factors	thyroid hormone receptor interactor 10
TAF13	1.28	0.01347	Transcription Factors	TAF13 RNA polymerase II, TATA box binding protein (TBP)-associated factor, 18kDa
YBX1	1.28	0.00183	Transcription Factors	Y box binding protein 1
MAX	1.28	0.00627	Transcription Factors	MYC associated factor X
TAF11	1.27	0.0002	Transcription Factors	TAF11 RNA polymerase II, TATA box binding protein (TBP)-associated factor, 28kDa
ZNF32	1.27	0.00372	Transcription Factors	zinc finger protein 32

ILF3	1.27	0.00035	Transcription Factors	interleukin enhancer binding factor 3
KLF16	1.27	0.00031	Transcription Factors	Kruppel-like factor 16
DBP	1.27	0.0269	Transcription Factors	D site of albumin promoter (albumin D-box) binding protein
ZNF184	1.27	0.00036	Transcription Factors	zinc finger protein 184
SMARCAL	1.26	0.00091	Transcription Factors	SWI/SNF related, matrix associated, actin dependent regulator of chromatin, subfamily a-like 1
PURA	1.26	0.00507	Transcription Factors	purine-rich element binding protein A
SMAD1	1.26	0.03102	Transcription Factors	SMAD family member 1
TFAM	1.26	0.03351	Transcription Factors	transcription factor A, mitochondrial
ZNF18	1.26	0.00122	Transcription Factors	zinc finger protein 18
ZFAND6	1.26	0.00034	Transcription Factors	zinc finger, AN1-type domain 6
PER3	1.25	0.01307	Transcription Factors	period circadian clock 3
SMARCAL	1.25	0.03439	Transcription Factors	SWI/SNF-related, matrix-associated actin-dependent regulator of chromatin, subfamily a, containing DEAD/H box 1
TAF9	1.24	0.01279	Transcription Factors	---
TFAP2D	1.24	0.00957	Transcription Factors	transcription factor AP-2 delta (activating enhancer binding protein 2 delta)
UBR4	1.24	0.00508	Transcription Factors	ubiquitin protein ligase E3 component n-recognin 4
GTF3C1	1.24	0.01733	Transcription Factors	general transcription factor IIIC subunit 1
RELA	1.24	0.00483	Transcription Factors	v-rel avian reticuloendotheliosis viral oncogene homolog A
ZNF16	1.24	0.0095	Transcription Factors	zinc finger protein 16
BATF3	1.24	0.00112	Transcription Factors	basic leucine zipper transcription factor, ATF-like 3
POU3F1	1.24	0.01602	Homeodomain Proteins	POU class 3 homeobox 1
ZNF324	1.23	0.00324	Transcription Factors	zinc finger protein 324
SMARCA2	1.23	0.04082	Transcription Factors	SWI/SNF related, matrix associated, actin dependent regulator of chromatin, subfamily a, member 2
E2F3	1.23	0.00548	Transcription Factors	E2F transcription factor 3
CBX1	1.23	0.01635	Transcription Factors	chromobox homolog 1
ATXN2	1.23	0.00099	Transcription Factors	ataxin 2
ZBTB32	1.23	0.01236	Transcription Factors	zinc finger and BTB domain containing 32
YY1	1.23	0.00723	Transcription Factors	YY1 transcription factor
GTF2F2	1.22	0.00765	Transcription Factors	general transcription factor IIF subunit 2
CHD1	1.21	0.00428	Transcription Factors	chromodomain helicase DNA binding protein 1
SRCAP	1.21	0.00242	Transcription Factors	Snf2-related CREBBP activator protein
PAX1	1.21	0.04162	Transcription Factors	paired box 1
ID1	1.21	0.00088	Transcription Factors	inhibitor of DNA binding 1, dominant negative helix-loop-helix protein
PHRF1	1.20	0.01917	Transcription Factors	PHD and ring finger domains 1
ATN1	1.20	0.02546	Transcription Factors	atrophin 1
SF1	1.20	0.0074	Transcription Factors	splicing factor 1
IKZF5	1.20	0.01581	Transcription Factors	IKAROS family zinc finger 5
NFKBIE	1.20	0.0034	Transcription Factors	nuclear factor of kappa light polypeptide gene enhancer in B-cells inhibitor, epsilon
SART3	1.20	0.013	Transcription Factors	squamous cell carcinoma antigen recognized by T-cells 3
MBNL2	1.20	0.01789	Transcription Factors	muscleblind-like splicing regulator 2
EP400	1.20	0.00155	Transcription Factors	E1A binding protein p400
ZNF2	1.20	0.02431	Transcription Factors	zinc finger protein 2
ZNF219	1.20	0.00441	Transcription Factors	zinc finger protein 219
BRF1	1.20	0.00204	Transcription Factors	BRF1, RNA polymerase III transcription initiation factor 90 kDa subunit
EZH1	1.20	0.00036	Transcription Factors	enhancer of zeste 1 polycomb repressive complex 2 subunit
SALL1	1.19	0.01024	Transcription Factors	spalt-like transcription factor 1
LMO7	1.19	0.03311	Transcription Factors	LIM domain 7
ZNF460	1.19	0.00385	Transcription Factors	zinc finger protein 460
FOXB1	1.19	0.0413	Transcription Factors	forkhead box B1
GTF2H2	1.19	0.0022	Transcription Factors	general transcription factor IIH subunit 2 /// general transcription factor IIH subunit 2B (pseudogene) /// GTF2H2 family member C /// GTF2H2 family member C, copy 2
ZNF408	1.19	4.6E-05	Transcription Factors	zinc finger protein 408
ARFGAP2	1.19	0.00186	Transcription Factors	ADP-ribosylation factor GTPase activating protein 2
MED26	1.19	0.01045	Transcription Factors	mediator complex subunit 26
ZNF133	1.19	0.00679	Transcription Factors	zinc finger protein 133
GTF3C4	1.19	0.00029	Transcription Factors	general transcription factor IIIC subunit 4
ZNF281	1.19	0.00851	Transcription Factors	zinc finger protein 281
NOBOX	1.19	0.00821	Homeodomain Proteins	NOBOX oogenesis homeobox
ZNF189	1.18	0.00445	Transcription Factors	zinc finger protein 189
RXRB	1.18	0.0046	Transcription Factors	retinoid X receptor beta
NFE2L1	1.18	0.00143	Transcription Factors	nuclear factor, erythroid 2-like 1
ISL2	1.18	0.01028	Homeodomain Proteins	ISL LIM homeobox 2
TAF10	1.18	0.00976	Transcription Factors	TAF10 RNA polymerase II, TATA box binding protein (TBP)-associated factor, 30kDa
BTF3P11	1.17	0.00194	Transcription Factors	basic transcription factor 3 pseudogene 11
MTA1	1.17	0.02118	Transcription Factors	metastasis associated 1
CBX6	1.16	0.00057	Transcription Factors	chromobox homolog 6
ZNF22	1.16	0.00161	Transcription Factors	zinc finger protein 22
TP53I13	1.16	0.00584	Transcription Factors	tumor protein p53 inducible protein 13
SUPT6H	1.16	0.02187	Transcription Factors	SPT6 homolog, histone chaperone
ZNF605	1.15	0.02308	Transcription Factors	zinc finger protein 605
ZNF416	1.15	0.00514	Transcription Factors	zinc finger protein 416
ZRANB2	1.15	0.00293	Transcription Factors	zinc finger, RAN-binding domain containing 2
ZBTB6	1.15	0.04154	Transcription Factors	zinc finger and BTB domain containing 6
ZNF81	1.15	0.00384	Transcription Factors	zinc finger protein 81
AFF1	1.15	0.00242	Oncogenes / Translocated Cancer Genes	AF4/FMR2 family, member 1
PAX7	1.15	0.01312	Oncogenes / Translocated Cancer Genes / Homeodomain Proteins	paired box 7
BAZ1A	1.15	0.03951	Transcription Factors	bromodomain adjacent to zinc finger domain 1A
MEIS3	1.15	0.02079	Homeodomain Proteins	Meis homeobox 3
ZMYM5	1.14	0.00826	Transcription Factors	zinc finger, MYM-type 5
SOX13	1.14	0.03786	Transcription Factors	SRY box 13
ZNF169	1.14	0.01876	Transcription Factors	zinc finger protein 169
RBBP9	1.14	0.03461	Transcription Factors	retinoblastoma binding protein 9
ZNF263	1.14	0.00856	Transcription Factors	zinc finger protein 263
RFX1	1.13	0.00853	Transcription Factors	regulatory factor X, 1 (influences HLA class II expression)
LIMA1	1.13	0.01569	Transcription Factors	LIM domain and actin binding 1
ZNF25	1.12	0.03747	Transcription Factors	zinc finger protein 25
ZSCAN22	1.12	0.02015	Transcription Factors	zinc finger and SCAN domain containing 22
TAF4B	1.11	0.00889	Transcription Factors	TAF4b RNA polymerase II, TATA box binding protein (TBP)-associated factor, 105kDa
FOXD1	1.11	0.01586	Transcription Factors	forkhead box D1
ZFAND5	1.11	0.0102	Transcription Factors	zinc finger, AN1-type domain 5
FOXN2	1.11	0.00122	Transcription Factors	forkhead box N2
ZNF207	1.11	0.0086	Transcription Factors	zinc finger protein 207
SMARCA4	1.10	0.00465	Tumor Suppressors.	SWI/SNF related, matrix associated, actin dependent regulator of chromatin, subfamily a, member 4
TFEB	1.10	0.02639	Oncogenes / Translocated Cancer Genes	transcription factor EB
BTF3	1.10	0.04272	Transcription Factors	basic transcription factor 3
CAND1	1.10	0.00454	Transcription Factors	cullin-associated and neddylation-dissociated 1
ARHGAP3	1.09	0.01447	Transcription Factors	Rho GTPase activating protein 35
CBX5	1.09	0.04289	Transcription Factors	chromobox homolog 5
PPP1R13L	1.09	0.02165	Transcription Factors	protein phosphatase 1, regulatory subunit 13 like
FOXJ3	1.09	0.01216	Transcription Factors	forkhead box J3
MYCBP	1.09	0.01409	Transcription Factors	---
NCOA6	1.08	0.01326	Transcription Factors	nuclear receptor coactivator 6
ZBTB48	1.08	0.01991	Transcription Factors	zinc finger and BTB domain containing 48
GABPA	1.07	0.03491	Transcription Factors	GA binding protein transcription factor alpha subunit
SIX5	1.05	0.02568	Homeodomain Proteins	SIX homeobox 5

Supplementary Table 5. The differentially expressed genes in apoptotic signaling pathway between ATRX-NC and ATRX-KO cells and their protein-protein interaction with parp1.

Gene	Fold Change	P	Interaction with PARP1	Gene Title	Alignments	Chromosomal Location	Gene Ontology Molecular Function
TNFRSF9	30.37	6.10E-06	---	tumor necrosis factor recep	chr1:7980382-8000887 (-) // 78.79	chr1p36	---
CLU	12.94	7.81E-05	---	clusterin	chr8:27471094-27472453 (-) // 46.3	chr8p21-p12	---
TGFB1	7.21	2.04E-05	---	transforming growth factor	chr19:41836227-41859788 (-) // 77	chr19q13.1	Cell cycle KEGG // GenMAPP /// TGF Beta Signaling Pathway // GenMAPP
IL1B	6.30	2.07E-04	---	interleukin 1 beta	chr2:113587336-113594352 (-) // 9	chr2q14	Smooth muscle contraction // GenMAPP
TP63	6.09	1.25E-03	---	tumor protein p63	chr3:189507589-189597967 (+) // 1	chr3q28	---
CYP1B1	5.28	5.21E-03	---	cytochrome P450, family 1	chr2:38294651-38303710 (-) // 98.6	chr2p22.2	---
IFI6	3.98	2.79E-03	---	interferon, alpha-inducible	chr1:27992580-27998708 (-) // 76.4	chr1p35	---
MLLT11	3.87	2.61E-05	---	myeloid/lymphoid or mixed	chr1:151039692-151040972 (+) // 9	chr1q21	---
FAS	3.68	1.24E-03	0.66	Fas cell surface death recep	chr10:90750326-90775541 (+) // 95	chr10q24.1	Apoptosis // GenMAPP /// Apoptosis GenMAPP // GenMAPP /// Apoptosis KEGG // GenMAPP
TGFB2	3.49	1.81E-04	---	transforming growth factor	chr1:218519493-218615478 (+) // 9	chr1q41	---
CAV1	3.19	7.61E-04	---	caveolin 1	chr7:116165091-116199607 (+) // 9	chr7q31.1	Integrin-mediated cell adhesion KEGG // GenMAPP
EDA2R	2.97	1.85E-03	---	ectodysplasin A2 receptor	chrX:65819325-65835862 (-) // 99.8	chrXq12	---
TNFRSF12A	2.90	1.10E-03	---	tumor necrosis factor recep	chr16:3070359-3072381 (+) // 96.69	chr16p13.3	---
CASP4	2.88	1.95E-03	---	caspase 4	chr11:104813624-104839300 (-) //	chr11q22.2-q22.3	Apoptosis // GenMAPP /// Apoptosis KEGG // GenMAPP
BID	2.83	2.69E-04	---	BH3 interacting domain de	chr22:18217977-18257226 (-) // 96	chr22q11.1	Apoptosis // GenMAPP /// Apoptosis GenMAPP // GenMAPP
TNFSF10	2.69	1.53E-02	0.707	tumor necrosis factor (ligan	chr3:172224175-172241264 (-) // 9	chr3q26	Apoptosis // GenMAPP /// Apoptosis GenMAPP // GenMAPP /// Apoptosis GenMAPP // GenMAPP
TLR3	2.54	1.18E-03	---	toll-like receptor 3	chr4:186990350-187006249 (+) // 9	chr4q35	---
LY96	2.46	1.44E-02	---	lymphocyte antigen 96	chr8:74903589-74941305 (+) // 93.9	chr8q21.11	---
PIK3R1	2.38	4.23E-05	---	phosphoinositide-3-kinase	chr5:67522116-67597647 (+) // 94.1	chr5q13.1	---
CDKN1A	2.28	6.64E-03	0.752	cyclin-dependent kinase in	chr6:36646505-36655108 (+) // 92.9	chr6p21.2	Cell cycle KEGG // GenMAPP /// G1 to S cell cycle Reactome // GenMAPP
IL1A	2.19	1.83E-03	---	interleukin 1 alpha	chr2:113531637-113542047 (-) // 6	chr2q14	Hypertrophy model // GenMAPP
CIB1	2.18	1.57E-03	---	calcium and integrin bindin	chr15:90773477-90777160 (-) // 94	chr15q25.3-q26	---
JUN	2.11	2.36E-02	---	jun proto-oncogene	chr1:59246365-59249733 (-) // 98.8	chr1p32-p31	Apoptosis // GenMAPP /// Apoptosis GenMAPP // GenMAPP /// MAPK Cascade // GenMAPP /// Smooth muscle contraction // GenMAPP /// TGF Beta Signaling Pathway // GenMAPP /// Wnt signaling // GenMAPP
TNFRSF21	2.01	8.57E-04	---	tumor necrosis factor recep	chr6:47200012-47277641 (-) // 91.6	chr6p21.1	Apoptosis // GenMAPP
SGPL1	1.92	4.41E-02	---	sphingosine-1-phosphate	chr10:72575787-72637339 (+) // 97	chr10q21	SPHINGOLIPID-SYN-PWY // HumanCyc
FADD	1.92	4.27E-03	0.658	Fas (TNFRSF6)-associated	chr11:70049442-70053461 (+) // 87	chr11q13.3	Apoptosis // GenMAPP /// Apoptosis GenMAPP // GenMAPP /// Apoptosis KEGG // GenMAPP
SCN2A	1.88	1.23E-02	---	sodium channel, voltage g	chr2:166150591-166246539 (+) // 9	chr2q24.3	---
CASP8	1.88	4.69E-04	0.893	caspase 8, apoptosis-relate	chr2:202098188-202146638 (+) // 9	chr2q33-q34	Apoptosis // GenMAPP /// Apoptosis GenMAPP // GenMAPP /// Apoptosis KEGG // GenMAPP
GPX1	1.87	5.07E-03	---	glutathione peroxidase 1	chr3:49394611-49396030 (-) // 98.1	chr3p21.3	PWY-4081 // HumanCyc
PML	1.85	2.41E-02	---	promyelocytic leukemia	chr15:74287013-74335678 (+) // 99	chr15q22	---
GABARAP	1.82	3.13E-04	---	GABA(A) receptor-associat	chr17:7143739-7145753 (-) // 96.43	chr17p13.1	---
CD24	1.82	2.42E-02	---	CD24 molecule	chrY:21152512-21154677 (-) // 75.6	chr6q21	---
APOPT1	1.81	2.79E-02	---	apoptogenic 1, mitochond	chr14:104029315-104056864 (+) //	chr14q32.33	---
TNFRSF11B	1.80	2.30E-03	---	tumor necrosis factor recep	chr8:119935802-119964189 (-) // 9	chr8q24	---
HIC1	1.75	1.79E-02	---	hypermethylated in cancer	chr17:1959964-1962129 (+) // 93.76	chr17p13.3	---
BAD	1.74	8.35E-03	---	BCL2-associated agonist o	chr11:64037301-64051883 (-) // 97	chr11q13.1	Apoptosis // GenMAPP /// Apoptosis KEGG // GenMAPP
TP73	1.74	3.34E-03	---	tumor protein p73	chr1:3607235-3649856 (+) // 99.95	chr1p36.3	Apoptosis // GenMAPP
DAP3	1.72	8.34E-04	---	death associated protein 3	chr1:155658873-155708317 (+) // 9	chr1q22	---
IL33	1.69	2.18E-02	---	interleukin 33	chr9:6241681-6257982 (+) // 99.81	chr9p24.1	---
IKBKE	1.67	1.74E-02	---	inhibitor of kappa light pol	chr1:206643837-206670222 (+) // 9	chr1q32.1	Apoptosis KEGG // GenMAPP
BLOC1S2	1.67	4.60E-02	---	biogenesis of lysosomal or	chr10:102034456-102046433 (-) //	chr10q24.31	---
CRH	1.66	6.13E-03	---	corticotropin releasing hor	chr8:67089083-67090691 (-) // 88.8	chr8q13	Smooth muscle contraction // GenMAPP
SMAD3	1.65	1.57E-02	0.916	SMAD family member 3	chr15:67358426-67485584 (+) // 98	chr15q22.33	---
SRGN	1.65	4.80E-02	---	serglycin	chr10:70847862-70848929 (+) // 91	chr10q22.1	---
ANXA6	1.65	9.82E-03	---	annexin A6	chr5:150480669-150537392 (-) // 9	chr5q33.1	Calcium regulation in cardiac cells // GenMAPP /// Prostaglandin synthesis regulation // GenMAPP
MKNK2	1.65	3.35E-02	---	MAP kinase interacting ser	chr19:2037469-2050872 (-) // 98.77	chr19p13.3	---
BCL3	1.60	3.15E-02	---	B-cell CLL/lymphoma 3	chr19:45251803-45263301 (+) // 81	chr19q13.1-q13.2	---
KITLG	1.58	5.45E-04	---	KIT ligand	chr12:88890596-88974238 (-) // 99	chr12q22	---
TFPT	1.58	1.26E-02	---	TCF3 (E2A) fusion partner	chr19:54610329-54619036 (-) // 99	chr19q13	---
DIABLO	1.56	1.14E-03	---	diablo, IAP-binding mitoch	chr12:122692315-122710580 (-) //	chr12q24.31	---
FIS1	1.56	1.97E-02	---	fission, mitochondrial 1	chr7:100882892-100888366 (-) // 9	chr7q22.1	---
BAG6	1.56	1.22E-03	---	BCL2 associated athanoge	chr6:31606817-31620412 (-) // 99.8	chr6p21.3	---
RIPK1	1.53	1.39E-02	---	receptor (TNFRSF)-interact	chr6:3077057-3114174 (+) // 94.76	chr6p25.2	Apoptosis // GenMAPP /// Apoptosis GenMAPP // GenMAPP /// Apoptosis KEGG // GenMAPP
SELK	1.53	7.24E-03	---	selenoprotein K	chr3:53919226-53925944 (-) // 91.4	chr3p21.31	---
NDUFA13	1.53	4.14E-03	---	NADH dehydrogenase (ub	chr19:19627051-19639006 (+) // 65	chr19p13.2	PWY-5083 // HumanCyc
ITGAV	1.51	1.14E-02	---	integrin alpha V	chr2:187454789-187545625 (+) // 9	chr2q31-q32	Integrin-mediated cell adhesion KEGG // GenMAPP
CD3E	1.51	6.50E-03	---	CD3e molecule, epsilon (CD	chr11:118175613-118186886 (+) //	chr11q23	---
TNFRSF10C	1.49	4.35E-03	---	tumor necrosis factor recep	chr8:22960498-22974950 (+) // 96.7	chr8p22-p21	---
G0S2	1.47	9.43E-03	---	G0/G1 switch 2	chr1:209848757-209849734 (+) // 9	chr1q32.2	Circadian Exercise // GenMAPP
DNAJC10	1.47	3.69E-02	---	DnaJ (Hsp40) homolog, su	chr2:183604842-183643836 (+) // 9	chr2q32.1	---
BCL2L1	1.47	1.67E-02	0.827	BCL2-like 1	chr20:30253663-30310155 (-) // 99	chr20q11.21	Apoptosis // GenMAPP /// Apoptosis GenMAPP // GenMAPP /// Apoptosis KEGG // GenMAPP
MLH1	1.45	4.91E-02	---	mutL homolog 1	chr3:37035017-37092336 (+) // 100	chr3p21.3	Ovarian Infertility Genes // GenMAPP
HRAS	1.44	7.68E-03	0.551	Harvey rat sarcoma viral on	chr11:532242-535538 (-) // 89.18 //	chr11p15.5	G Protein Signaling // GenMAPP /// MAPK Cascade // GenMAPP /// TGF Beta Signaling Pathway // GenMAPP
STK24	1.43	2.76E-03	---	serine/threonine kinase 24	chr13:99097406-99100098 (-) // 77	chr13q31.2-q32.3	---
ITPR1	1.43	2.36E-02	---	inositol 1,4,5-trisphosphate	chr3:4535135-4889081 (+) // 99.77	chr3p26.1	Calcium regulation in cardiac cells // GenMAPP /// G Protein Signaling // GenMAPP /// Smooth muscle contraction // GenMAPP
EP300	1.42	1.86E-02	0.624	E1A binding protein p300	chr22:41487789-41576081 (+) // 95	chr22q13.2	Cell cycle KEGG // GenMAPP /// TGF Beta Signaling Pathway // GenMAPP
ABL1	1.34	8.57E-03	---	ABL proto-oncogene 1, no	chr9:133710452-133763060 (+) // 9	chr9q34.1	Cell cycle KEGG // GenMAPP
PERP	1.34	2.08E-02	---	PERP, TP53 apoptosis effec	chr6:138416384-138419852 (-) // 7	chr6q24	---
MAPK9	1.33	1.22E-02	---	mitogen-activated protein	chr5:179661794-179707610 (-) // 9	chr5q35	TGF Beta Signaling Pathway // GenMAPP /// Wnt signaling // GenMAPP
ERN1	1.31	2.98E-02	---	endoplasmic reticulum to n	chr17:62120759-62207476 (-) // 97	chr17q24.2	---
HINT1	1.30	3.32E-02	---	histidine triad nucleotide b	chr5:130494977-130497736 (-) // 5	chr5q31.2	---
HIPK1	1.30	4.59E-03	---	homeodomain interacting	chr1:114471995-114514680 (+) // 9	chr1p13.2	---
NGF	1.29	2.04E-02	---	nerve growth factor (beta	chr1:115828538-115880857 (-) // 9	chr1p13.1	Apoptosis KEGG // GenMAPP
NGFRAP1	1.29	1.27E-02	---	nerve growth factor recept	chrX:102632108-102632999 (+) // 9	chrXq22.2	---
CUL2	1.27	4.58E-02	---	cullin 2	chr10:35297478-35379237 (-) // 89	chr10p11.21	---
SHISA5	1.27	3.70E-02	---	shisa family member 5	chr3:48509275-48514558 (-) // 96.8	chr3p21.31	---
RNF41	1.26	4.55E-02	---	ring finger protein 41, E3 u	chr12:56598284-56615704 (-) // 98	chr12q13.13	---
IFI16	1.20	4.09E-02	---	interferon, gamma-inducib	chr1:158979707-159024938 (+) // 9	chr1q22	---
TMBIM6	1.13	2.81E-02	---	transmembrane BAX inhib	chr12:50135362-50156784 (+) // 98	chr12q13.12	---
DPF2	1.11	1.98E-03	---	D4, zinc and double PHD f	chr11:65101354-65120451 (+) // 99	chr11q13	---
ADORA1	0.95	4.57E-02	---	adenosine A1 receptor	chr1:203096954-203136532 (+) // 9	chr1q32.1	GPCRDB Class A Rhodopsin-like // GenMAPP /// Nucleotide GPCRs // GenMAPP
ST20	0.92	1.15E-02	---	suppressor of tumorigenici	chr15:80191246-80216044 (-) // 84	chr15q25.1	PWY-2201 // HumanCyc
TP53BP2	0.85	1.54E-02	---	tumor protein p53 binding	chr1:223967790-224033619 (-) // 9	chr1q41	---
MSH6	0.81	3.84E-02	0.674	mutS homolog 6	chr2:48010285-48034036 (+) // 98.6	chr2p16	---
ZMAT3	0.81	2.74E-03	---	zinc finger, matrin-type 3	chr3:178742721-178789570 (-) // 9	chr3q26.32	---
ERCC6	0.77	1.02E-02	---	excision repair cross-comp	chr10:50666708-50747072 (-) // 98	chr10q11.23	---
GGCT	0.76	2.93E-02	---	gamma-glutamylcyclotran	chr7:30536238-30540064 (-) // 76.2	chr7p15-p14	---
ATF4	0.74	2.37E-03	---	activating transcription fac	chr22:39916568-39918685 (+) // 98	chr22q13.1	Smooth muscle contraction // GenMAPP
PRKDC	0.74	1.61E-02	0.882	protein kinase, DNA-activa	chr8:48685668-48872743 (-) // 99.9	chr8q11	Cell cycle KEGG // GenMAPP
FNIP2	0.74	2.61E-02	---	folliculin interacting protei	chr4:159791419-159793881 (+) // 7	chr4q32.1	---
FOXO3	0.72	4.71E-02	0.445	forkhead box O3	chr6:108881487-109001968 (+) // 9	chr6q21	---
IL6R	0.69	1.22E-02	---	interleukin 6 receptor	chr1:154377668-154439327 (+) // 8	chr1q21	---
INHBA	0.68	4.11E-03	---	inhibin beta A	chr7:41728795-41740059 (-) // 89.7	chr7p15-p13	TGF Beta Signaling Pathway // GenMAPP
SORT1	0.66	2.46E-03	---	sortilin 1	chr1:109855074-109940551 (-) // 9	chr1p13.3|1p21.3-p13.1	---
BRCA2	0.64	2.15E-02	---	breast cancer 2, early onse	chr13:32889616-32973805 (+) // 98	chr13q12.3	---
CHEK2	0.64	1.49E-02	---	checkpoint kinase 2	chr22:29083837-29130716 (-) // 96	chr22q12.1	Cell cycle KEGG // GenMAPP
HIPK2	0.62	6.85E-04	---	homeodomain interacting	chr7:139248031-139250382 (-) // 9	chr7q34	---
CUL4A	0.62	2.40E-02	---	cullin 4A	chr13:113864286-113919392 (+) //	chr13q34	---
DYRK2	0.57	8.54E-03	---	dual specificity tyrosine-(Y	chr12:68042753-68059186 (+) // 96	chr12q15	---
PDK1	0.55	1.98E-03	---	pyruvate dehydrogenase k	chr2:173420827-173460830 (+) // 9	chr2q31.1	Krebs-TCA Cycle // GenMAPP
BCL2L11	0.50	7.14E-04	---	BCL2-like 11 (apoptosis fac	chr2:111881322-111921808 (+) // 1	chr2q13	Apoptosis // GenMAPP
ATM	0.47	1.20E-03	0.792	ATM serine/threonine kina	chr11:108157903-108161365 (+) //	chr11q22-q23	Cell cycle KEGG // GenMAPP /// G1 to S cell cycle Reactome // GenMAPP /// Ovarian Infertility Genes // GenMAPP
P2RX7	0.41	2.00E-02	---	purinergic receptor P2X, lig	chr12:121570751-121622638 (+) //	chr12q24	---
UACA	0.40	3.22E-03	---	uveal autoantigen with coi	chr15:70949272-70954035 (-) // 57	chr15q22-q24	---
ERBB3	0.39	3.44E-04	---	erb-b2 receptor tyrosine k	chr12:56487110-56487771 (+) // 94	chr12q13	---
DCC	0.30	5.77E-03	---	DCC netrin 1 receptor	chr18:49867157-51057288 (+) // 99	chr18q21.3	---
ZNF385D	0.27	6.94E-05	---	zinc finger protein 385D	chr3:21462649-21792816 (-) // 95.3	chr3p24.3	---
PRKCD	0.18	3.03E-05	---	protein kinase C, delta	chr3:53195405-53226297 (+) // 99.2	chr3p21.31	Calcium regulation in cardiac cells // GenMAPP /// G Protein Signaling // GenMAPP /// Smooth muscle contraction // GenMAPP /// Wnt signaling // GenMAPP
MAP3K5	2.12	8.10E-02	0.441	mitogen-activated protein	chr6:136878765-137113564 (-) // 9	chr6q22.33	---
SFN	1.89	5.46E-02	---	stratifin	chr1:27189652-27190947 (+) // 95.3	chr1p36.11	Calcium regulation in cardiac cells // GenMAPP /// Smooth muscle contraction // GenMAPP
BRCA1	1.68	6.52E-02	---	breast cancer 1, early onse	chr17:41196317-41277131 (-) // 92	chr17q21	---
PYCARD	1.65	6.89E-02	---	PYD and CARD domain co	chr16:31212808-31214251 (-) // 97	chr16p11.2	---
FASTK	1.53	1.17E-01	---	Fas-activated serine/threo	chr7:150773719-150777874 (-) // 9	chr7q35	---
IFI27	1.51	1.58E-01	---	interferon, alpha-inducible	chr14:94577975-94583032 (+) // 96	chr14q32	---
TNFRSF10A	1.50	9.75E-02	0.596	tumor necrosis factor recep	chr8:22968801-23082639 (-) // 84.2	chr8p21	---
CASP9	1.49	1.22E-01	0.977	caspase 9	chr1:15818770-15850740 (-) // 88.5	chr1p36.21	Apoptosis // GenMAPP /// Apoptosis GenMAPP // GenMAPP /// Apoptosis KEGG // GenMAPP
SOD2	1.43	8.44E-02	---	superoxide dismutase 2, m	chr6:160100148-160100389 (+) // 9	chr6q25.3	---
DDIT3	1.43	6.94E-02	---	DNA-damage-inducible tr	chr12:57910376-57914294 (-) // 93	chr12q13.1-q13.2	mRNA processing Reactome // GenMAPP
P2RX4	1.40	1.98E-01	---	purinergic receptor P2X, lig	chr12:121647941-121671547 (+) //	chr12q24.32	---
SART1	1.39	9.48E-02	---	squamous cell carcinoma a	chr11:65729213-65746565 (+) // 91	chr11q13.1	---
NFATC4	1.38	5.82E-02	---	nuclear factor of activated	chr14:24837405-24846941 (+) // 98	chr14q11.2	---
SIVA1	1.37	1.75E-01	---	SIVA1, apoptosis-inducing	chr14:105219501-105225990 (+) //	chr14q32.33	---
TRADD	1.36	6.54E-02	0.443	TNFRSF1A-associated via d	chr16:67188103-67193780 (-) // 88	chr16q22	Apoptosis // GenMAPP /// Apoptosis GenMAPP // GenMAPP /// Apoptosis KEGG // GenMAPP
NOL3	1.36	1.84E-01	---	nucleolar protein 3 (apopto	chr16:67204450-67206877 (+) // 59	chr16q22.1	---
BAX	1.35	5.87E-02	0.83	BCL2-associated X protein	chr19:49458803-49464354 (+) // 86	chr19q13.3-q13.4	Apoptosis // GenMAPP /// Apoptosis GenMAPP // GenMAPP /// Apoptosis KEGG // GenMAPP
BCL2	1.34	2.74E-01	0.952	B-cell CLL/lymphoma 2	chr18:60792945-60987361 (-) // 94	chr18q21.3	Apoptosis // GenMAPP /// Apoptosis GenMAPP // GenMAPP /// Apoptosis KEGG // GenMAPP
ZMAT1	1.33	9.40E-02	---	zinc finger, matrin-type 1	chrX:101137262-101142053 (-) // 8	chrXq21	---
MELK	1.31	7.79E-02	---	maternal embryonic leucin	chr9:36572872-36677678 (+) // 99.9	chr9p13.2	---
TNFRSF1A	1.31	8.92E-02	0.491	tumor necrosis factor recep	chr12:6437939-6451149 (-) // 88.57	chr12p13.2	Apoptosis // GenMAPP /// Apoptosis GenMAPP // GenMAPP /// Apoptosis KEGG // GenMAPP /// Inflammatory Response Pathway // GenMAPP
IL12A	1.30	1.54E-01	---	interleukin 12A	chr3:159706783-159713543 (+) // 9	chr3q25.33	---
CD27	1.30	2.33E-01	---	CD27 molecule	chr12:6554161-6560879 (+) // 99.83	chr12p13	---
GSK3A	1.30	7.84E-02	---	glycogen synthase kinase 3	chr19:42734345-42746731 (-) // 96	chr19q13.2	Glycogen Metabolism // GenMAPP
DDX5	1.30	1.94E-01	---	DEAD (Asp-Glu-Ala-Asp)	chr17:62495739-62502407 (-) // 99	chr17q21	---
SSTR3	1.28	8.59E-02	---	somatostatin receptor 3	chr22:37602585-37608535 (-) // 87	chr22q13.1	GPCRDB Class A Rhodopsin-like // GenMAPP /// Peptide GPCRs // GenMAPP
ZNF622	1.28	1.61E-01	---	zinc finger protein 622	chr5:16451628-16465896 (-) // 97.9	chr5p15.1	---
PAWR	1.27	1.55E-01	---	PRKC, apoptosis, WT1, regu	---	chr12q21	---
RHOT2	1.25	7.81E-02	---	ras homolog family membe	chr16:718179-724169 (+) // 98.01 /	chr16p13.3	---
PDPK1	1.24	1.48E-01	---	3-phosphoinositide depen	chr16:2588033-2647908 (+) // 99.95	chr16p13.3	Integrin-mediated cell adhesion KEGG // GenMAPP
DEDD2	1.24	1.21E-01	---	death effector domain con	chr19:42702749-42721874 (-) // 92	chr19q13.2	---
CASP12	1.23	3.34E-01	---	caspase 12 (gene/pseudoc	chr11:104757416-104769397 (-) //	chr11q22.3	---
DAPK3	1.23	1.09E-01	---	death-associated protein k	chr19:3958451-3969828 (-) // 96.65	chr19p13.3	---
PPP1R15A	1.22	2.53E-01	---	protein phosphatase 1, reg	chr19:49375677-49379314 (+) // 99	chr19q13.2	---
CASP3	1.22	2.06E-01	0.997	caspase 3	chr4:185548850-185570576 (-) // 9	chr4q34	Apoptosis // GenMAPP /// Apoptosis GenMAPP // GenMAPP /// Apoptosis KEGG // GenMAPP
TNFRSF4	1.21	2.26E-01	---	tumor necrosis factor recep	chr1:1146871-1149512 (-) // 65.62	chr1p36	---
CASP10	1.21	4.20E-01	0.584	caspase 10	chr2:202047891-202084803 (+) // 7	chr2q33-q34	Apoptosis // GenMAPP /// Apoptosis KEGG // GenMAPP
TNFRSF18	1.21	2.96E-01	---	tumor necrosis factor recep	chr1:1138888-1141951 (-) // 91.79	chr1p36.3	---
ZNF346	1.19	2.42E-01	---	zinc finger protein 346	chr5:176449707-176492984 (+) // 9	chr5q35.2	---
PRODH	1.19	3.75E-01	---	proline dehydrogenase 1, m	chr22:18900207-18909087 (-) // 82	chr22q11.21	CITRULBIO-PWY // HumanCyc /// PROLINE-DEG2-PWY // HumanCyc /// PROUT-PWY // HumanCyc
BTK	1.19	2.72E-01	---	Bruton agammaglobuliner	chrX:100604441-100641182 (-) // 9	chrXq21.33-q22	---
TNFRSF1B	1.18	4.85E-01	---	tumor necrosis factor recep	chr1:12227059-12269276 (+) // 86.5	chr1p36.22	Apoptosis // GenMAPP /// Apoptosis GenMAPP // GenMAPP /// Apoptosis KEGG // GenMAPP /// Inflammatory Response Pathway // GenMAPP
TNFRSF10B	1.18	3.38E-01	0.625	tumor necrosis factor recep	chr8:22863895-22876848 (-) // 96.9	chr8p22-p21	Apoptosis // GenMAPP
TNFRSF8	1.17	5.09E-01	---	tumor necrosis factor recep	chr1:12123433-12204199 (+) // 99.6	chr1p36	---
IFNG	1.17	2.68E-01	---	interferon, gamma	chr12:68548773-68553522 (-) // 93	chr12q14	Hypertrophy model // GenMAPP /// Inflammatory Response Pathway // GenMAPP /// Proteasome Degradation // GenMAPP /// TGF Beta Signaling Pathway // GenMAPP
BAK1	1.17	1.00E-01	0.719	BCL2-antagonist/killer 1	chr20:31276725-31278806 (-) // 97	chr6p21.3	Apoptosis // GenMAPP /// Apoptosis GenMAPP // GenMAPP
CD28	1.17	5.63E-01	---	CD28 molecule	chr2:204571197-204602556 (+) // 8	chr2q33	Inflammatory Response Pathway // GenMAPP
CD70	1.16	4.39E-01	---	CD70 molecule	chr19:6585866-6591150 (-) // 96.06	chr19p13	---
PDCD6	1.16	1.64E-01	---	programmed cell death 6	chr5:271804-314916 (+) // 99.89 //	chr5p15.33	---
BCL2L2	1.16	5.84E-01	0.462	BCL2-like 2	chr14:23776411-23778835 (+) // 92	chr14q11.2-q12	Apoptosis KEGG // GenMAPP
GSK3B	1.15	1.94E-01	---	glycogen synthase kinase 3	chr3:119545547-119812513 (-) // 9	chr3q13.3	Cell cycle KEGG // GenMAPP /// Glycogen Metabolism // GenMAPP /// Wnt signaling // GenMAPP
E2F1	1.15	4.82E-01	0.606	E2F transcription factor 1	chr20:32263488-32274197 (-) // 93	chr20q11.2	Cell cycle KEGG // GenMAPP /// G1 to S cell cycle Reactome // GenMAPP
CRIP1	1.15	4.25E-01	---	cysteine-rich protein 1 (int	chr14:105953596-105955027 (+) //	chr14q32.33	---
TRAF2	1.15	1.56E-01	---	TNF receptor-associated fa	chr9:139793163-139821059 (+) // 9	chr9q34	Apoptosis // GenMAPP /// Apoptosis GenMAPP // GenMAPP /// Apoptosis KEGG // GenMAPP
BNIP3	1.15	1.01E-01	---	BCL2/adenovirus E1B 19kD	chr10:133781942-133795382 (-) //	chr10q26.3	---
SHH	1.14	1.48E-01	---	sonic hedgehog	chr7:155595558-155604967 (-) // 9	chr7q36	---
BBC3	1.14	3.48E-01	---	BCL2 binding component 3	chr19:47724083-47734451 (-) // 87	chr19q13.3-q13.4	---
SPN	1.13	3.77E-01	---	sialophorin	chr16:29674299-29676837 (-) // 84	chr16p11.2	---
PMAIP1	1.12	2.95E-01	0.598	phorbol-12-myristate-13-	chr18:57567191-57571537 (+) // 98	chr18q21.32	---
CD14	1.12	4.57E-01	---	CD14 molecule	chr5:140011320-140012772 (-) // 9	chr5q31.1	Electron Transport Chain // GenMAPP
SIRT1	1.12	3.26E-01	0.642	sirtuin 1	chr10:69644426-69678141 (+) // 96	chr10q21.3	---
NBN	1.12	2.49E-01	0.949	nibrin	chr8:90945755-90996926 (-) // 77.9	chr8q21	---
MYBBP1A	1.11	7.11E-01	---	MYB binding protein (P160	chr17:4442200-4458644 (-) // 97.55	chr17p13.3	---
TNFRSF10D	1.11	1.45E-01	0.408	tumor necrosis factor recep	chr8:22995289-23021527 (-) // 99.9	chr8p21	---
TOPORS	1.11	1.77E-01	0.427	topoisomerase I binding, a	chr9:32540823-32550819 (-) // 97.9	chr9p21	---
E2F2	1.11	6.15E-01	---	E2F transcription factor 2	chr1:23836347-23857712 (-) // 99.9	chr1p36	Cell cycle KEGG // GenMAPP /// G1 to S cell cycle Reactome // GenMAPP
CASP2	1.10	6.62E-02	0.763	caspase 2	chr7:142986689-143002915 (+) // 9	chr7q34-q35	Apoptosis // GenMAPP /// Apoptosis GenMAPP // GenMAPP /// Apoptosis KEGG // GenMAPP
ATP2A1	1.09	2.45E-01	---	ATPase, Ca++ transporting	chr16:28889992-28915560 (+) // 99	chr16p12.1	---
BCL2L10	1.09	3.15E-01	---	BCL2-like 10 (apoptosis fac	chr15:52402044-52404923 (-) // 10	chr15q21	---
CD40	1.09	4.30E-01	---	CD40 molecule, TNF recep	chr20:44746935-44757752 (+) // 94	chr20q12-q13.2	Apoptosis KEGG // GenMAPP /// Inflammatory Response Pathway // GenMAPP
RRP8	1.09	2.05E-01	---	ribosomal RNA processing	chr11:6613310-6616293 (-) // 38.51	chr11p15.4	---
RELT	1.09	6.36E-01	---	RELT tumor necrosis factor	chr11:73105302-73108518 (+) // 74	chr11q13.4	---
PTGIS	1.08	5.72E-01	---	prostaglandin I2 (prostacyc	chr20:48120413-48184707 (-) // 67	chr20q13.13	Eicosanoid Synthesis // GenMAPP /// Prostaglandin synthesis regulation // GenMAPP /// PWY66-374 // MetaCyc
BRSK2	1.08	3.66E-01	---	BR serine/threonine kinase	chr11:1411487-1481862 (+) // 97.74	chr11p15.5	---
TNFRSF14	1.08	6.63E-01	---	tumor necrosis factor recep	chr1:2487837-2495188 (+) // 96.67	chr1p36.32	---
CUL3	1.08	1.54E-01	---	cullin 3	chr2:225334660-225450097 (-) // 9	chr2q36.2	---
DAXX	1.08	6.39E-01	---	death-domain associated p	chr6:33286402-33290724 (-) // 95.0	chr6p21.3	Apoptosis KEGG // GenMAPP
CIDEB	1.07	6.02E-01	---	cell death-inducing DFFA-	chr14:24774397-24780595 (-) // 90	chr14q12	GPCRDB Other // GenMAPP
FASLG	1.07	6.21E-01	---	Fas ligand (TNF superfamil	chr1:172628157-172636014 (+) // 9	chr1q23	Apoptosis // GenMAPP /// Apoptosis GenMAPP // GenMAPP /// Apoptosis KEGG // GenMAPP
DNM1L	1.07	5.99E-01	---	dynamin 1-like	chr12:32832223-32896543 (+) // 99	chr12p11.21	---
UBE4B	1.07	1.30E-01	---	ubiquitination factor E4B	chr1:10093027-10241294 (+) // 99.0	chr1p36.3	---
RPS27L	1.06	5.40E-01	---	ribosomal protein S27-like	chr15:63446054-63449663 (-) // 97	chr15q22.2	---
PTH	1.06	8.04E-01	---	parathyroid hormone	chr11:13513601-13517526 (-) // 99	chr11p15.3-p15.1	---
C22orf29	1.05	5.96E-01	0.48	chromosome 22 open read	chr22:19837763-19839671 (-) // 89	chr22q11.21	---
DAPL1	1.05	7.35E-01	---	death associated protein lik	chr2:159651838-159672501 (+) // 9	chr2q24.1	---
EPHA2	1.05	6.02E-01	---	EPH receptor A2	chr1:16450832-16482540 (-) // 99.0	chr1p36	---
HIP1	1.04	7.14E-01	---	huntingtin interacting prot	chr7:75283612-75368287 (-) // 76.9	chr7q11.23	---
PPP1R13B	1.04	8.83E-01	---	protein phosphatase 1, reg	chr14:104200089-104212825 (-) //	chr14q32.33	---
NF1	1.04	6.41E-01	---	neurofibromin 1	chr17:29559160-29592293 (+) // 96	chr17q11.2	---
PARP2	1.03	6.35E-01	0.942	poly(ADP-ribose) polymera	chr14:20811798-20826062 (+) // 98	chr14q11.2	---
AIFM1	1.03	6.16E-01	0.948	apoptosis-inducing factor,	chrX:129263509-129299672 (-) // 9	chrXq26.1	---
HMOX1	1.03	8.02E-01	---	heme oxygenase 1	chr22:35777086-35790194 (+) // 99	chr22q13.1	---
BNIP3L	1.03	5.20E-01	---	BCL2/adenovirus E1B 19kD	chr8:26240544-26270640 (+) // 96.5	chr8p21	Apoptosis // GenMAPP
DEDD	1.03	8.46E-01	---	death effector domain con	chr1:161091814-161094302 (-) // 1	chr1q23.3	---
CYCS	1.02	6.34E-01	0.828	cytochrome c, somatic	chr7:25162450-25164877 (-) // 97.4	chr7p15.3	Apoptosis // GenMAPP /// Apoptosis GenMAPP // GenMAPP /// Apoptosis KEGG // GenMAPP /// Electron Transport Chain // GenMAPP
PPM1F	1.02	8.53E-01	---	protein phosphatase, Mg2	chr22:22279289-22300481 (-) // 76	chr22q11.22	---
MCL1	1.02	4.74E-01	---	myeloid cell leukemia 1	chr1:150547032-150552247 (-) // 9	chr1q21	Apoptosis GenMAPP // GenMAPP /// Apoptosis KEGG // GenMAPP
TP53	1.01	8.70E-01	0.941	tumor protein p53	chr17:7571738-7590917 (-) // 83.38	chr17p13.1	Apoptosis // GenMAPP /// Apoptosis GenMAPP // GenMAPP /// Cell cycle KEGG // GenMAPP /// G1 to S cell cycle Reactome // GenMAPP
CCK	1.01	9.10E-01	---	cholecystokinin	chr3:42299315-42306249 (-) // 98.5	chr3p22.1	---
CRADD	1.01	8.70E-01	---	CASP2 and RIPK1 domain	chr12:94071166-94244295 (+) // 98	chr12q21.33-q23.1	Apoptosis KEGG // GenMAPP
POLB	1.00	8.79E-01	0.983	polymerase (DNA directed	chr8:42196029-42229313 (+) // 100	chr8p11.2	Nucleotide Metabolism // GenMAPP
TNFSF12	1.00	8.84E-01	---	tumor necrosis factor (ligan	chr17:7452453-7461205 (+) // 99.69	chr17p13	---
NUPR1	1.00	9.76E-01	---	nuclear protein 1, transcrip	chr16:28548681-28550283 (-) // 90	chr16p11.2	---
BOK	1.00	9.98E-01	---	BCL2-related ovarian killer	chr2:242498305-242513533 (+) // 9	chr2q37.3	Apoptosis KEGG // GenMAPP
XBP1	1.00	9.98E-01	---	X-box binding protein 1	chr22:28795305-29196524 (-) // 97	chr22q12.1|22q12	---
DDX3X	1.00	7.87E-01	---	DEAD (Asp-Glu-Ala-Asp)	chrX:41193414-41197878 (+) // 89.3	chrXp11.3-p11.23	---
RIPK3	1.00	9.14E-01	---	receptor-interacting serine	chr14:24805230-24809186 (-) // 98	chr14q11.2	---
CEBPB	0.99	9.78E-01	---	CCAAT/enhancer binding	chr20:48807124-48809209 (+) // 75	chr20q13.1	Circadian Exercise // GenMAPP /// Ovarian Infertility Genes // GenMAPP
KRT8	0.99	9.28E-01	---	keratin 8, type II	chr8:62490762-62492299 (+) // 97.1	chr12q13	---
SENP1	0.99	8.93E-01	---	SUMO1/sentrin specific pe	chr12:48439104-48491908 (-) // 99	chr12q13.1	---
HTRA2	0.99	8.68E-01	---	HtrA serine peptidase 2	chr2:74757116-74760300 (+) // 97.4	chr2p12	---
PPARD	0.99	8.89E-01	---	peroxisome proliferator-ac	chr6:35310403-35395955 (+) // 93.9	chr6p21.2	Nuclear Receptors // GenMAPP
CUL1	0.99	9.62E-01	---	cullin 1	chr7:148427094-148497799 (+) // 9	chr7q36.1	---
ARL6IP5	0.98	7.08E-01	---	ADP-ribosylation factor lik	chr3:69134093-69155217 (+) // 96.4	chr3p14	---
SGPP1	0.98	9.71E-01	---	sphingosine-1-phosphate	chr14:64151922-64194693 (-) // 95	chr14q23.2	---
WWOX	0.98	7.45E-01	---	WW domain containing ox	chr16:78198123-79246376 (+) // 98	chr16q23	---
LAMP1	0.98	8.01E-01	---	lysosomal-associated mem	chr13:113960857-113976816 (+) //	chr13q34	---
BMF	0.98	9.96E-01	---	Bcl2 modifying factor	chr15:40380090-40398649 (-) // 92	chr15q14	---
CD5	0.98	8.81E-01	---	CD5 molecule	chr11:60869960-60894518 (+) // 99	chr11q13	---
PPP2R5C	0.97	4.19E-01	---	protein phosphatase 2, reg	chr14:102276233-102372983 (+) //	chr14q32.31	Glycogen Metabolism // GenMAPP /// Wnt signaling // GenMAPP
TLR4	0.97	7.75E-01	---	toll-like receptor 4	chr9:120466583-120477220 (+) // 9	chr9q33.1	---
STK25	0.96	4.43E-01	---	serine/threonine kinase 25	chr2:242434514-242447578 (-) // 9	chr2q37.3	---
PGAP2	0.96	5.84E-01	---	post-GPI attachment to pr	chr11:3842564-3847580 (+) // 93.3	chr11p15.5	---
NGFR	0.94	6.96E-01	---	nerve growth factor recept	chr17:47572666-47592361 (+) // 94	chr17q21-q22	Apoptosis KEGG // GenMAPP

LGALS12	0.94	4.39E-01	---	lectin, galactoside-binding	chr11:63275859-63284246 (+) // 10	chr11q13	---
STK11	0.94	6.90E-01	0.602	serine/threonine kinase 11	chr19:1206574-1228093 (+) // 99.9	chr19p13.3	---
PHLDA3	0.94	5.83E-01	---	pleckstrin homology-like d	chr1:201434622-201438233 (-) // 9	chr1q31	---
APAF1	0.94	6.00E-01	---	apoptotic peptidase activat	chr12:99039077-99129210 (+) // 88	chr12q23	Apoptosis // GenMAPP /// Apoptosis GenMAPP // GenMAPP /// Apoptosis KEGG // GenMAPP
ZMAT4	0.94	6.94E-01	---	zinc finger, matrin-type 4	chr8:40388112-40755343 (-) // 99.9	chr8p11.21	---
FHIT	0.93	2.20E-01	---	fragile histidine triad	chr3:59737946-61237124 (-) // 69.4	chr3p14.2	---
MOAP1	0.93	5.88E-01	---	modulator of apoptosis 1	chr14:93648540-93651241 (-) // 43	chr14q32	---
MAEL	0.93	9.01E-01	---	maelstrom spermatogenic	chr1:166958519-166991455 (+) // 9	chr1q24.1	---
CHAC1	0.93	9.51E-01	---	ChaC glutathione-specific	chr15:41245690-41248709 (+) // 95	chr15q15.1	---
PDK2	0.93	2.46E-01	---	pyruvate dehydrogenase k	chr17:48172685-48188732 (+) // 98	chr17q21.33	Krebs-TCA_Cycle // GenMAPP
KRT18	0.92	4.79E-01	---	keratin 18, type I	chr12:53342910-53346694 (+) // 99	chr12q13	---
TICAM1	0.91	6.48E-01	---	toll-like receptor adaptor r	chr19:4815943-4817980 (-) // 95.2	chr19p13.3	---
USP28	0.91	2.89E-01	---	ubiquitin specific peptidase	chr11:113669577-113746250 (-) //	chr11q23	---
SNW1	0.91	5.54E-01	---	SNW domain containing 1	chr14:78183945-78227496 (-) // 98	chr14q24.3	---
DIDO1	0.91	1.42E-01	---	death inducer-obliterator	chr20:61518565-61542416 (-) // 98	chr20q13.33	---
APPL1	0.91	2.18E-01	---	adaptor protein, phosphot	chr3:57261853-57307496 (+) // 93.7	chr3p14.3	---
RHOT1	0.90	3.66E-01	---	ras homolog family memb	chr17:30509843-30552745 (+) // 99	chr17q11.2	---
SST	0.90	2.75E-01	---	somatostatin	chr3:187386699-187388202 (-) // 9	chr3q28	---
DAP	0.90	3.34E-01	---	death-associated protein	chr5:10679405-10761339 (-) // 98.4	chr5p15.2	---
PDCD10	0.89	5.34E-01	---	programmed cell death 10	chr3:167401696-167452594 (-) // 9	chr3q26.1	---
BAG3	0.89	4.46E-01	---	BCL2-associated athanoge	chr10:121410881-121437296 (+) //	chr10q25.2-q26.2	---
EYA2	0.89	2.82E-01	---	EYA transcriptional coactiv	chr20:45618647-45817490 (+) // 98	chr20q13.1	---
ZNF385C	0.89	1.64E-01	---	zinc finger protein 385C	chr17:40190249-40190702 (-) // 10	chr17q21.2	---
CASP8AP2	0.89	1.66E-01	---	caspase 8 associated prote	chr6:90572736-90584148 (+) // 92.7	chr6q15	---
CD38	0.88	3.67E-01	---	CD38 molecule	chr4:15779974-15850486 (+) // 99.2	chr4p15	PYRIDNUCSAL-PWY // HumanCyc /// PYRIDNUCSAL-PWY // MetaCyc
MFF	0.88	1.06E-01	---	mitochondrial fission facto	chr2:228193473-228222543 (+) // 9	chr2q36.3	---
UBE2K	0.87	1.39E-01	---	ubiquitin conjugating enzy	chr4:39699870-39781582 (+) // 96.6	chr4p14	---
MAPT	0.87	1.42E-01	---	microtubule associated pro	chr17:43971829-44103471 (+) // 93	chr17q21.1	---
DAB2IP	0.85	4.59E-01	---	DAB2 interacting protein	chr9:124522495-124547809 (+) // 9	chr9q33.1-q33.3	---
PRKCA	0.85	3.41E-01	---	protein kinase C, alpha	chr17:64802385-64803325 (+) // 98	chr17q22-q23.2	Calcium regulation in cardiac cells // GenMAPP /// G Protein Signaling // GenMAPP /// Smooth muscle contraction // GenMAPP /// Wnt signaling // GenMAPP
IL2	0.84	4.24E-01	0.4	interleukin 2	chr4:123372624-123377642 (-) // 9	chr4q26-q27	Inflammatory Response Pathway // GenMAPP
TMEM109	0.83	1.05E-01	---	transmembrane protein 10	chr11:60681676-60690913 (+) // 99	chr11q12.2	---
ACVR1B	0.83	2.97E-01	---	activin A receptor type IB	chr12:52345501-52389473 (+) // 98	chr12q13	---
JAK2	0.83	5.14E-01	---	Janus kinase 2	chr9:5065804-5068158 (+) // 78.23	chr9p24	---
RAF1	0.82	1.23E-01	0.421	Raf-1 proto-oncogene, se	chr3:12629484-12631279 (-) // 13.7	chr3p25	MAPK Cascade // GenMAPP
TM2D1	0.79	3.55E-01	---	TM2 domain containing 1	chr1:62163035-62165517 (-) // 32.4	chr1p31.3	---
CUL5	0.79	6.94E-02	---	cullin 5	chr11:107879646-107978739 (+) //	chr11q22.3	---
TNFRSF11A	0.78	8.56E-02	---	tumor necrosis factor rece	chr18:59992547-60053502 (+) // 88	chr18q22.1	---
IL4	0.77	2.22E-01	---	interleukin 4	chr5:132009677-132018370 (+) // 9	chr5q31.1	Inflammatory Response Pathway // GenMAPP
XPA	0.76	9.32E-02	---	xeroderma pigmentosum,	chr9:100437191-100459600 (-) // 1	chr9q22.3	---
TIMM50	0.76	1.79E-01	---	translocase of inner mitoch	chr19:39982607-39983501 (+) // 14	chr19q13.2	---
DAPK1	0.76	1.58E-01	---	death-associated protein k	chr9:90112795-90323543 (+) // 98.7	chr9q21.33	---
DDIT4	0.75	1.91E-01	---	DNA damage inducible tra	chr10:74033681-74035793 (+) // 98	chr10q22.1	---
KIAA0141	0.74	9.59E-02	---	KIAA0141	chr5:141303396-141319717 (+) // 7	chr5q31.3	---
TNF	0.73	5.01E-02	---	tumor necrosis factor	chr6:2887602-2888080 (+) // 21.82	chr6p21.3	Apoptosis // GenMAPP /// Apoptosis GenMAPP // GenMAPP /// Apoptosis KEGG // GenMAPP /// Matrix Metalloproteinases // GenMAPP /// TGF Beta Signaling Pathway // GenMAPP
FGFR3	0.73	1.23E-01	---	fibroblast growth factor re	chr4:1795622-1810598 (+) // 95.75	chr4p16.3	GLUCONSUPER-PWY // HumanCyc
TRIB3	0.70	1.03E-01	---	tribbles pseudokinase 3	chr20:361674-377379 (+) // 95.29 /	chr20p13-p12.2	---
BCL2A1	0.69	1.10E-01	---	BCL2-related protein A1	chr15:80253234-80263538 (-) // 99	chr15q24.3	Apoptosis KEGG // GenMAPP
ZNF385B	0.68	7.26E-02	---	zinc finger protein 385B	chr2:180307296-180440995 (-) // 8	chr2q31.2-q31.3	---
TNFRSF25	0.68	1.51E-01	---	tumor necrosis factor rece	chr1:6521211-6526235 (-) // 97.05 /	chr1p36.2	Apoptosis // GenMAPP
AEN	0.65	6.75E-02	---	apoptosis enhancing nucle	chr15:89169755-89175512 (+) // 91	chr15q26.1	---

Supplementary Table 6. The Pearson correlation among ATRX and genes in apoptotic signaling pathway.

	R	P value	P value summary
ATRX vs. ATM	0.41	<0.0001	****
ATRX vs. BCL2L1	-0.30	<0.0001	****
ATRX vs. CASP8	-0.03	0.7512	ns
ATRX vs. CDKN1A	-0.16	0.038	*
ATRX vs. EP300	0.42	<0.0001	****
ATRX vs. FADD	-0.47	<0.0001	****
ATRX vs. FAS	-0.13	0.0961	ns
ATRX vs. FOXO3	0.44	<0.0001	****
ATRX vs. HRAS	-0.40	<0.0001	****
ATRX vs. MSH6	0.32	<0.0001	****
ATRX vs. PRKDC	0.55	<0.0001	****
ATRX vs. SMAD3	0.33	<0.0001	****
ATRX vs. TNFSF10	-0.11	0.1653	ns
